# Modified sini powder for the management of postoperative depression in non-small cell lung cancer patients: a multicenter, randomized, double-blind, placebo-controlled trial protocol

**DOI:** 10.3389/fphar.2026.1805554

**Published:** 2026-06-19

**Authors:** Yuyang Jin, Jing Wang, Huakang Li, Ming Fan, Shuo Zhang, Bing Lin, Yuanzhen Mi, Cuicui Gong, Pengxuan Gu, Ke Xu, Biao Zhao, Jinyi Lang, Meihua Chen

**Affiliations:** 1 School of Sports Medicine and Health, Chengdu Sports University, Chengdu, China; 2 School of Clinical Medicine, Southwest Medical University, Luzhou, Sichuan, China; 3 Department of Radiation Oncology, Precision Radiation in Oncology Key Laboratory of Sichuan Province, Sichuan Clinical Research Center for Cancer, Sichuan Cancer Hospital and Institute, Sichuan Cancer Center, University of Electronic Science and Technology of China, Chengdu, China; 4 School of Clinical Medicine, Chengdu University of Traditional Chinese Medicine, Chengdu, Sichuan, China; 5 Health Management Center, Hospital of Chengdu University of Traditional Chinese Medicine, Chengdu, Sichuan, China; 6 Department of Integrative Chinese and Western Medicine, Sichuan Clinical Research Center for Cancer, Sichuan Cancer Hospital and Institute, Sichuan Cancer Center, University of Electronic Science and Technology of China, Chengdu, China

**Keywords:** non-small cell lung cancer, postoperative depression, randomized controlled trial, traditional Chinese medicine, trial protocol

## Abstract

**Background:**

Postoperative depression is a common complication following non-small cell lung cancer (NSCLC) resection, which adversely affects quality of life and may impede long-term survival. Modified Sini Powder (MSNP), a modified traditional Chinese medicine formula derived from classical Sini Powder, has been used in clinical practice to alleviate depressive symptoms. However, comprehensive evidence from well-designed clinical trials remains insufficient. This protocol describes a study designed to evaluate the efficacy and safety of MSNP in managing postoperative mild-to-moderate depression in patients with NSCLC and to characterize its biological mechanisms.

**Methods:**

In this multicenter, randomized, double-blind, placebo-controlled trial, 468 postoperative patients with NSCLC and mild-to-moderate depression will be recruited across nine tertiary medical centers. Participants will be stratified by concomitant paroxetine use and randomized in a 1:1 ratio within each stratum to receive an MSNP-containing intervention or the corresponding placebo control for 6 weeks. The primary outcome is the total effective rate at the end of treatment, defined as a ≥25% reduction in the 17-item Hamilton Depression Rating Scale score from baseline. Secondary outcomes include measures of anxiety, sleep quality, fear of cancer recurrence, quality of life, and survival. Safety will be monitored through adverse event reporting and laboratory examinations. Mechanistic explorations will integrate analyses of inflammatory cytokines, hypothalamic-pituitary-adrenal axis hormones, neurotransmitters, brain-derived neurotrophic factor, serum metabolomics, and gut microbiota profiles.

**Discussion:**

This trial aims to provide robust clinical evidence for the integration of MSNP into the management of postoperative mild-to-moderate depression in patients with NSCLC. Furthermore, multi-omics analyses are expected to elucidate the multi-target and multi-pathway mechanisms of MSNP, offering insights into personalized and integrative therapeutic strategies.

**Trial Registration:**

Registered on September 1, 2025, at the International Traditional Medicine Clinical Trial Registration Center (https://itmctr.ccebtcm.org.cn/mgt/project/view/1962361959055753216; Identifier: ITMCTR2025001642).

## 1 Background

Lung cancer remains the leading cause of cancer-related mortality worldwide, with non-small cell lung cancer (NSCLC) accounting for approximately 80%–85% of all lung cancer cases (Abdelaziz et al., 2019). For early- and middle-stage NSCLC, surgical resection is considered the most effective and potentially curative treatment strategy. However, even after complete resection, nearly half of patients with stage I–IIIA NSCLC experience recurrence and eventually die (Imai et al., 2021). This grim prognosis, compounded by common postoperative physical discomfort, functional impairment, and economic burden, creates multiple psychological stressors for NSCLC patients. Previous studies have shown that postoperative NSCLC patients frequently suffer from psychological distress, primarily manifesting as depression, anxiety, insomnia, fear of recurrence, shame, and guilt (Cao et al., 2025).

Depression, as one of the most prevalent psychological disorders post-surgery, significantly impacts patients’ quality of life and survival prognosis. A prospective cohort study involving 350 postoperative NSCLC patients and 100 healthy controls reported a depression prevalence rate of 29.7% in the patient group, which was significantly higher than the 5.0% in the control group. Further survival analysis revealed a strong association between depression and shorter disease-free survival and overall survival (OS) (Deng and Chen, 2023). The authoritative National Comprehensive Cancer Network guidelines also explicitly recommend the inclusion of psychological distress screening and intervention, including depression, as part of standard cancer supportive care (Sanft et al., 2023).

Psychotherapy and oral antidepressants are the primary interventions for depression. The American Society of Clinical Oncology guidelines recommend psychotherapy as a first-line intervention for managing depression in cancer patients (Andersen et al., 2023). A series of evidence-based psychotherapeutic strategies, including cognitive behavioral therapy (CBT), behavioral activation, interpersonal therapy, mindfulness-based stress reduction, and acceptance and commitment therapy, have been supported by research (Andersen et al., 2023). Among these approaches, CBT is currently one of the most widely recommended and well-established interventions. However, CBT usually requires multiple structured sessions over several weeks or months. For postoperative patients with NSCLC, impaired physical function, fatigue, pain, and frequent follow-up visits may hinder their ability to complete a full course of psychotherapy. Notably, previous research has shown that the treatment gap for psychological interventions exceeds 90% in low- and middle-income countries and remains above 50% even in high-income countries (Thornicroft et al., 2017). Major factors contributing to this gap include a shortage of trained mental health professionals, which often leads to prolonged waiting times before patients can receive psychotherapy; insufficient public resources or financial support for specialized mental health services, which may impose a considerable economic burden on patients; and social stigma, which may discourage patients from seeking psychological care because of shame or embarrassment (Atif et al., 2022). Taken together, these factors restrict the accessibility, timeliness, and sustained engagement with psychotherapy in real-world clinical practice, particularly among postoperative patients with NSCLC.

In most clinical settings in China, pharmacological treatments remain the most common approach. Selective serotonin reuptake inhibitors (SSRIs) are the most widely used first-line antidepressants, with representatives such as paroxetine, fluoxetine, sertraline, citalopram, and escitalopram. SSRIs work by selectively blocking the serotonin transporter on the presynaptic membrane, thereby increasing serotonin levels in the synaptic cleft and improving depressive symptoms (Seiger et al., 2021). Notably, some studies have suggested that SSRIs may possess potential antitumor properties, which has attracted interest in their use among cancer patients (Özkaya Gül and Aydemir, 2025). However, treatment response to SSRIs remains incomplete. When a 50% reduction in the 17-item Hamilton Depression Rating Scale (HAMD-17) is used as the response criterion, the response rate to SSRIs is approximately 60% (Zhao et al., 2019). In addition, common adverse effects of SSRIs, including gastrointestinal discomfort, dizziness, sleep disturbance, weight gain, and sexual dysfunction, may compromise treatment adherence and tolerability (Edinoff et al., 2022).

Against this background, traditional Chinese medicine (TCM) has gained increasing attention as a safe, cost-effective, low-toxicity, and multi-target intervention for cancer-related depression. According to TCM theory, liver qi stagnation is key to the pathogenesis of depression, and the treatment principle focuses on soothing the liver and regulating qi (Chen et al., 2021). A meta-analysis has shown that TCM formulas based on this therapeutic principle, whether used alone or in combination with antidepressants or psychotherapy, can help alleviate depressive symptoms in cancer patients (Wang et al., 2019). However, the analysis also highlighted significant methodological flaws in the included trials, such as insufficient reporting, high risk of bias, and substantial heterogeneity, suggesting the need for further high-quality studies to validate these findings.

Sini Powder (SNP), a classic TCM formula originating from *Shanghan Lun*, consists of four botanical drugs: Chaihu [*Bupleurum chinense* DC. (Apiaceae; *Bupleuri Radix*)], Baishao [*Paeonia lactiflora* Pall. (Paeoniaceae; *Paeoniae Radix Alba*)], Zhishi [*Citrus × aurantium* f. *aurantium*. (Rutaceae; *Aurantii Fructus Immaturus*)], and Gancao [*Glycyrrhiza uralensis* Fisch. ex DC. (Fabaceae; Glycyrrhizae Radix et Rhizoma)], and is known for its ability to soothe the liver and regulate qi. Preliminary evidence from small-scale clinical trials, animal studies, and *in vitro* experiments supports its antidepressant effects (He et al., 2022). Modified Sini Powder (MSNP) is an improved version of the classical Sini Powder (SNP), developed by adding Fuling [*Poria cocos* (Schw.) Wolf. (Polyporaceae; *Poria*)] and Suanzaoren [*Ziziphus jujuba* Mill. (Rhamnaceae; *Ziziphi Spinosae Semen*)] to improve sleep (Deng et al., 2022), as well as Hehuanpi [*Albizia julibrissin* Durazz. (Fabaceae; *Albiziae Cortex*)] to relieve anxiety (Lu et al., 2023), aiming to address the commonly co-occurring symptoms of anxiety and insomnia in clinical depression and to enhance patients’ quality of life from multiple dimensions.

This study plans to conduct a nationwide, multicenter, randomized, double-blind, placebo-controlled clinical trial, with stratification based on the concomitant use of paroxetine, to comprehensively assess the efficacy and safety of MSNP granules for the treatment of postoperative depression in NSCLC patients. Furthermore, considering the multi-target and multi-pathway characteristics of TCM, this study will integrate various omics analyses, including inflammatory markers, hormones associated with the hypothalamic-pituitary-adrenal (HPA) axis, neurotransmitters, brain-derived neurotrophic factor (BDNF), serum metabolomics profiles, and gut microbiota composition, to explore the potential mechanisms of MSNP and build a comprehensive evidence base.

## 2 Methods

### 2.1 Study design

**FIGURE 1:**
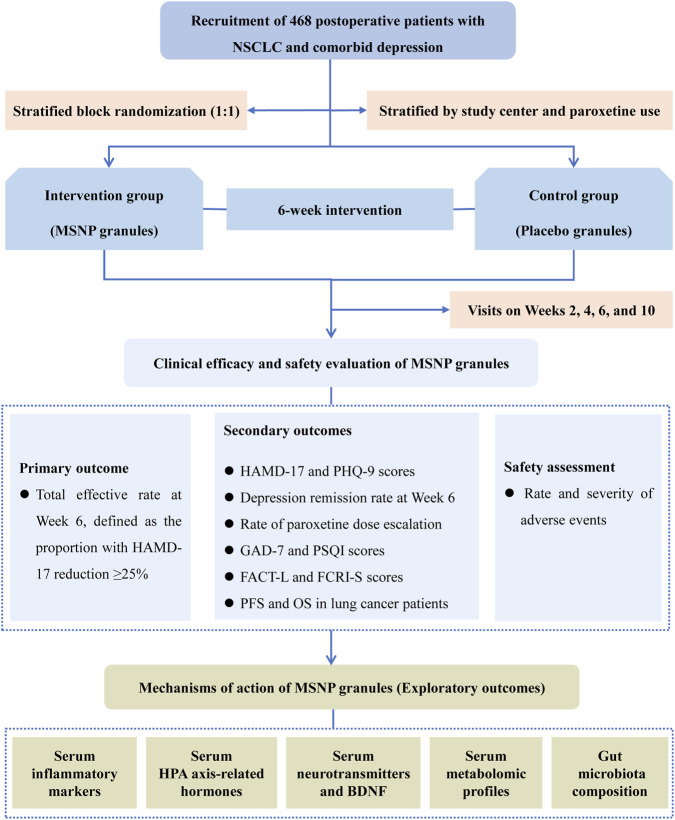
Study flowchart. BDNF, brain-derived neurotrophic factor; FACT-L, Functional Assessment of Cancer Therapy–Lung; FCRI-S, Fear of Cancer Recurrence Inventory–Severity Subscale; GAD-7, Generalized Anxiety Disorder-7; HAMD-17, 17-item Hamilton Depression Rating Scale; HPA, hypothalamic-pituitary-adrenal; MSNP, modified Sini Powder; NSCLC, non-small cell lung cancer; OS, overall survival; PHQ-9, Patient Health Questionnaire-9; PFS, progression-free survival; PSQI, Pittsburgh Sleep Quality Index.

This study is a nationwide, multicenter, randomized, double-blind, placebo-controlled clinical trial employing a parallel-group design. A total of 468 postoperative patients with NSCLC and comorbid depression will be enrolled from nine tertiary medical centers across nine provinces in China. Eligible participants will be stratified according to concomitant paroxetine use and randomly assigned in a 1:1 ratio within each stratum to the MSNP-containing intervention group or the corresponding placebo control group. The study protocol was developed and reported in compliance with the Standard Protocol Items: Recommendations for Interventional Trials (SPIRIT) 2025 guidelines (Supplementary Material 1). A detailed study flowchart is presented in Figure 1.

### 2.2 Trial setting

Participant recruitment will be conducted at nine tertiary medical centers across nine provinces in China: Sichuan Cancer Hospital; Guang’anmen Hospital, China Academy of Chinese Medical Sciences; First Teaching Hospital of Tianjin University of Traditional Chinese Medicine; Liaoning Cancer Hospital; Shanxi Cancer Hospital; Yueyang Hospital of Integrated Traditional Chinese and Western Medicine, Shanghai University of Traditional Chinese Medicine; Guangdong Provincial Hospital of Chinese Medicine; Zhejiang Provincial Hospital of Chinese Medicine; and Jiangsu Provincial Hospital of Chinese Medicine. These study sites include oncology-specialized hospitals as well as hospitals specializing in traditional Chinese medicine or integrated traditional Chinese and Western medicine. Their clinical resources, research infrastructure, and experience in multicenter trials will support participant recruitment, intervention delivery, outcome assessment, and follow-up.

### 2.3 Eligibility criteria

#### 2.3.1 Inclusion criteria

Participants meeting all of the following criteria will be eligible for enrollment:

1. Aged between 18 and 80 years, regardless of gender;
2. Diagnosis of depression according to the Diagnostic and Statistical Manual of Mental Disorders, Fifth Edition criteria, confirmed by licensed psychiatrists at participating centers;
3. Mild to moderate depression, defined as a HAMD-17 score of 8–23;
4. Meeting the traditional Chinese medicine syndrome diagnosis of liver qi stagnation according to the Guidelines for the Diagnosis and Treatment of Depressive Disorders by Integrating Chinese and Western Medicine (Liu et al., 2025), as assessed by licensed traditional Chinese medicine practitioners;
5. Histologically confirmed NSCLC, classified as stage I-IIIA according to the 8th edition of the American Joint Committee on Cancer staging system;
6. Having undergone radical lung cancer resection, with no more than 6 months elapsed since surgery;
7. Expected survival time ≥6 months as assessed by the treating oncologist;
8. Karnofsky Performance Status (KPS) score ≥70;
9. No previous use of antidepressant medication before enrollment.

#### 2.3.2 Exclusion criteria

Participants meeting any of the following criteria will be excluded from enrollment:

1. Pregnant or breastfeeding women;
2. Meeting DSM-5 diagnostic criteria, as confirmed by licensed psychiatrists, for psychiatric disorders other than depressive disorder that may interfere with trial participation, treatment adherence, or outcome assessment, such as schizophrenia, bipolar disorder, or substance- or alcohol-related disorders (Ju et al., 2025);
3. Current presence of severe suicidal ideation or history of suicide attempts;
4. History of organic brain disorders;
5. Presence of serious physical illnesses requiring active treatment;
6. Abnormal results in routine blood tests and biochemical parameters (hepatic function, renal function, and electrolytes), exceeding the normal laboratory reference ranges;
7. Use of antibiotics or probiotics within the past 12 weeks (which may affect gut microbiota analysis);
8. Currently receiving or expected to receive postoperative cancer therapies during the study period, including but not limited to radiotherapy, chemotherapy, immunotherapy, or targeted therapy;
9. Known allergy to any botanical drug in MSNP.

### 2.4 Interventions

All participants enrolled in this trial will be antidepressant-naïve before enrollment. In China, traditional Chinese medicine is commonly used either alone or in combination with antidepressants for the treatment of depression. Therefore, this trial will adopt a stratified design according to whether paroxetine is initiated after enrollment. Before randomization, qualified psychiatrists at each study center will determine whether paroxetine is clinically indicated, based on clinical assessment and discussion with the participant. This decision will not be influenced by subsequent allocation to MSNP or placebo. Within each stratum, participants will be randomly assigned in a 1:1 ratio to receive either MSNP granules or placebo granules. Accordingly, this design will generate four treatment conditions: MSNP alone, placebo alone, MSNP plus paroxetine, and placebo plus paroxetine.

Participants in the combination-therapy stratum will receive paroxetine for 6 weeks. The dosing regimen will be 10 mg once daily on Days 1–2, followed by 20 mg once daily from Day 3 onward, taken orally as a whole tablet after breakfast. Dose adjustment will be allowed according to clinical response and tolerability, with a maximum daily dose of 50 mg. Paroxetine tablets will be supplied by Zhejiang Huahai Pharmaceutical Co., Ltd., with the National Medical Products Administration approval number H20031106.

Participants in the intervention group will receive MSNP granules, whereas those in the control group will receive placebo granules. The placebo granules will contain 5% MSNP granule material blended with pharmacologically inert excipients to mimic the appearance, odor, taste, and texture of MSNP. Both MSNP and placebo granules will be manufactured by Sichuan Neo-Green Pharmaceutical Technology Development Co., Ltd. In accordance with Good Manufacturing Practice requirements and relevant National Medical Products Administration regulations. The two formulations will be indistinguishable in appearance, texture, odor, and taste.

MSNP or placebo granules will be administered orally for 6 weeks, one sachet twice daily, 30 min after breakfast and 30 min before bedtime, dissolved in warm water. During the study period, except for protocol-specified paroxetine, the use of other antidepressants, psychotherapy, acupuncture, other traditional Chinese medicine prescriptions for depression, or over-the-counter products with potential antidepressant effects will be prohibited. Concomitant medications required for other medical conditions will be permitted when clinically necessary and recorded in detail.

The allocated intervention may be discontinued or modified in cases of withdrawal of informed consent, serious adverse events, marked worsening of depressive symptoms, severe suicidal ideation or suicidal behavior, pregnancy, use of prohibited treatments, poor adherence, or any condition that, in the investigator’s judgment, makes continued treatment unsafe. Adherence will be promoted and assessed through standardized medication instructions, regular reminders, medication diaries, and return and counting of unused medication. Participants who discontinue the intervention will be encouraged to complete follow-up assessments unless they withdraw consent.

### 2.5 Composition and quality control of MSNP

MSNP consists of seven botanical drugs. All botanical materials are sourced from qualified suppliers adhering to the Quality Management Guidelines for Cultivation and Collection of Medicinal Plants in China. None of the species used are listed in the Appendices of the Convention on International Trade in Endangered Species of Wild Fauna and Flora (CITES), and their sourcing complies with ethical and sustainable harvesting standards. Sichuan Neo-Green Pharmaceutical Technology Development Co., Ltd. Processes these raw materials into granules in accordance with the Chinese Pharmacopoeia national standards. Detailed information on these standards can be found on the official website of the Chinese Pharmacopoeia Commission (https://www.chp.org.cn/#/newsDetail?id=15980) by using the standard number. The specific granule standards are as follows: Chaihu [*Bupleurum chinense* DC. (Apiaceae; *Bupleuri Radix*)] granules follow standard number YBZ-PFKL-2021030, with a crude drug-to-extract ratio of 3.5:1; Baishao [*Paeonia lactiflora* Pall. (Paeoniaceae; *Paeoniae Radix Alba*)] granules follow standard number YBZ-PFKL-2021002, with a crude drug-to-extract ratio of 4.5:1; Zhishi [*Citrus × aurantium* f. *aurantium*. (Rutaceae; *Aurantii Fructus Immaturus*)] granules follow standard number YBZ-PFKL-2021152, with a crude drug-to-extract ratio of 3.3:1; Gancao [*Glycyrrhiza uralensis* Fisch. ex DC. (Fabaceae; Glycyrrhizae Radix et Rhizoma)] granules follow standard number YBZ-PFKL-2021049, with a crude drug-to-extract ratio of 3:1; Fuling [*Poria cocos* (Schw.) Wolf. (Polyporaceae; *Poria*)] granules follow standard number YBZ-PFKL-2021241, with a crude drug-to-extract ratio of 12.5:1; Suanzaoren [*Ziziphus jujuba* Mill. (Rhamnaceae; *Ziziphi Spinosae Semen*)] granules follow standard number YBZ-PFKL-2021191, with a crude drug-to-extract ratio of 4:1; Hehuanpi [*Albizia julibrissin* Durazz. (Fabaceae; *Albiziae Cortex*)] granules follow standard number YBZ-PFKL-2021056, with a crude drug-to-extract ratio of 10:1. Each sachet of MSNP is composed of the aforementioned individual granules, mixed in accordance with the doses shown in Table 1.

**TABLE 1:** Composition of modified sini san powdered granules.

Chinese name	Medicinal part	Extract dose (Equivalent to crude drug)
Chaihu [*Bupleurum chinense* DC. (Apiaceae; *Bupleuri Radix*)]	Rhizome	1.71 g (6 g)
Baishao [*Paeonia lactiflora* Pall. (Paeoniaceae; *Paeoniae Radix Alba*)]	Rhizome	1.33 g (6 g)
Zhishi [*Citrus × aurantium* f. *aurantium*. (Rutaceae; *Aurantii Fructus Immaturus*)]	Fruit	1.82 g (6 g)
Gancao [*Glycyrrhiza uralensis* Fisch. ex DC. (Fabaceae; *Glycyrrhizae Radix et Rhizoma*)]	Rhizome	2 g (6 g)
Fuling [*Poria cocos* (Schw.) Wolf. (Polyporaceae; *Poria*)]	Sclerotium	0.48 g (6 g)
Suanzaoren [*Ziziphus jujuba* Mill. (Rhamnaceae; *Ziziphi Spinosae Semen*)]	Seed	1 g (4 g)
Hehuanpi [*Albizia julibrissin* Durazz. (Fabaceae; *Albiziae Cortex*)]	Bark	0.4 g (4 g)

The botanical drugs and their extracts used in MSNP meet the definition of “A-type extracts,” as they are included in national or regional pharmacopoeias and are used as active ingredients in phytopharmaceuticals with regulated medical use, including licensed, listed, or registered medicines. MSNP is a compound formula prepared from the aforementioned seven botanical drugs and processed into granules using modern pharmaceutical procedures, including decoction, extraction, concentration, and drying. In accordance with the Consensus statement on the Phytochemical Characterisation of Medicinal Plant Extracts (ConPhyMP) guidelines, quality control analysis of MSNP was performed using ultra-performance liquid chromatography (UPLC) and high-performance liquid chromatography (HPLC) (Supplementary Material 2). To evaluate the batch-to-batch consistency of MSNP granules, five batches of MSNP granules (S1–S5) were subjected to UPLC fingerprint analysis and HPLC-based quantification of representative marker compounds. UPLC fingerprint similarity was assessed using the Similarity Evaluation System for Chromatographic Fingerprint of Traditional Chinese Medicine (version 2012.130,723). The similarity values between each batch and the reference fingerprint ranged from 0.986 to 0.996, and all pairwise batch similarities were above 0.95, indicating good batch-to-batch consistency. In addition, four representative marker compounds, including synephrine, paeoniflorin, glycyrrhizic acid, and saikosaponin B2, were quantified by HPLC. The analytical method was validated for linearity, precision, repeatability, recovery, stability, and specificity. These results support the batch-to-batch chemical consistency and quality control of MSNP granules. Furthermore, microbial limit testing and tests for heavy metals (lead, cadmium, mercury, arsenic, copper) and pesticide residues all complied with the relevant requirements of the Chinese Pharmacopoeia (Supplementary Material 3). Based on the legal standards for source medicinal materials, their long-standing clinical use, and the rigorous production and quality control measures described above, the safety risks of MSNP for the target population are considered manageable.

**FIGURE 2:**
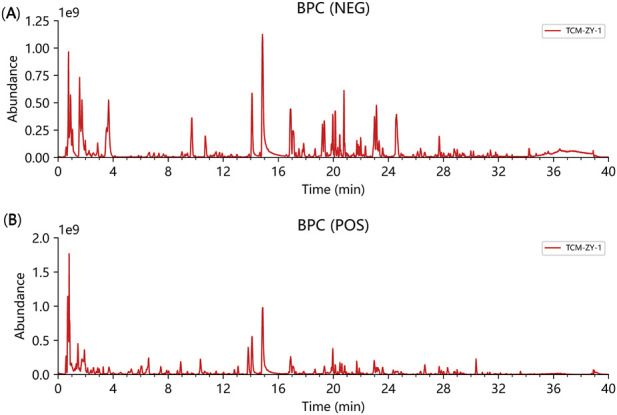
Base-peak chromatogram (BPC) of MSNP. **(A)** Negative-ion scan. **(B)** Positive-ion scan.

To characterize the chemical profile of MSNP, ultra-performance liquid chromatography coupled with Q-Exactive Orbitrap mass spectrometry (UPLC-QE-Orbitrap-MS) was employed. Representative base peak chromatograms (BPC) acquired in positive and negative ionization modes are displayed in Figure 2. Comparative analysis of accurate mass-to-charge ratios (*m/z*), MS/MS fragmentation patterns, and isotopic distributions against the LuMet-TCM reference database led to the identification of 1,671 metabolites in MSNP (Supplementary Material 4). The identified metabolites are primarily classified into the following categories: flavonoids (358 metabolites, 21.42%), phenylpropanoids (265 metabolites, 15.86%), terpenes (243 metabolites, 14.54%), carbohydrates and glycosides (142 metabolites, 8.50%), organoheterocyclic compounds (81 metabolites, 4.85%), lipids and lipid-like molecules (65 metabolites, 3.89%), alkaloids (57 metabolites, 3.41%), fatty acyls (47 metabolites, 2.81%), and steroids (26 metabolites, 1.56%), along with additional minor categories.

### 2.6 Outcomes

#### 2.6.1 Primary outcome

The primary outcome of this trial is the total effective rate at the end of the 6-week treatment period, defined as the proportion of participants achieving a ≥25% reduction in their HAMD-17 total score from baseline (Wang et al., 2019). The HAMD-17 is an observer-rated instrument designed to assess depressive symptoms in individuals with a clinical diagnosis of depression. According to standard thresholds, a HAMD-17 score of 0–7 indicates no depression, 8–16 indicates mild depression, 17–23 indicates moderate depression, and ≥24 indicates severe depression (Zimmerman et al., 2013). Treatment response is categorized into four groups based on the percentage reduction in HAMD-17 scores from baseline: recovery (≥75% reduction), markedly effective (≥50% reduction), improved (≥25% reduction), and ineffective (<25% reduction) (Wang et al., 2019). The total effective rate is defined as the proportion of participants classified as recovered, markedly effective, or improved.

#### 2.6.2 Secondary outcomes

1. Change from baseline in HAMD-17 total scores. Assessments will be performed at baseline, during the treatment phase (Weeks 2, 4, and 6), and at the Week 10 follow-up visit.
2. Depression remission rate at the end of the 6-week treatment period, defined as the proportion of participants achieving a HAMD-17 total score of ≤7 (Zhao et al., 2019).
3. Change from baseline in Patient Health Questionnaire-9 (PHQ-9) total scores. The PHQ-9 is a self-administered tool used to assess the severity of depressive symptoms over the past 2 weeks. It consists of 9 items, each scored from 0 to 3, with total scores ranging from 0 to 27. Scores of 0–4 are considered normal; 5–9 indicate mild depression; 10–14, moderate depression; 15–19, moderately severe depression; and 20–27, severe depression (Sun et al., 2020). Assessments will be conducted at baseline and at Weeks 2, 4, 6, and 10.
4. Proportion of participants requiring an increased dosage of paroxetine during the 6-week treatment period.
5. Change from baseline in Generalized Anxiety Disorder-7 (GAD-7) total scores. The GAD-7 is a self-reported screening and assessment tool for anxiety symptoms over the past 2 weeks. It consists of 7 items scored from 0 to 3, with total scores ranging from 0 to 21. Higher scores indicate greater anxiety severity, with a score ≥10 is generally considered clinically significant (Aktürk et al., 2025). Assessments will be performed at baseline, and at Weeks 2, 4, 6, and 10.
6. Change from baseline in Pittsburgh Sleep Quality Index (PSQI) total scores. The PSQI assesses sleep quality over the past month across seven domains, each scored from 0 to 3, with total scores ranging from 0 to 21. Scores of 0–5 indicate very good sleep quality; 6–10, good; 11–15, average; and >15, poor sleep quality (Wang et al., 2024). Assessments will be performed at baseline, and at Weeks 6 and 10.
7. Change from baseline in Functional Assessment of Cancer Therapy–Lung (FACT-L) total scores. The FACT-L is a validated instrument assessing health-related quality of life in patients with lung cancer. It consists of 36 items scored from 0 to 4, with total scores ranging from 0 to 144. The scale includes a general cancer subscale (27 items) and a lung cancer-specific subscale (9 items) (Cella et al., 1995). Higher scores indicate better quality of life. Assessments will be performed at baseline, and at Weeks 6 and 10.
8. Change from baseline in Fear of Cancer Recurrence Inventory–Severity Subscale (FCRI-S) total scores. The FCRI-S assesses the severity of fear of cancer recurrence over the past month (Savard et al., 2023). It consists of 9 items scored from 0 to 4, with total scores ranging from 0 to 36. Higher scores indicate greater fear of cancer recurrence severity, with a score ≥13 generally considered clinically significant (Simard and Savard, 2015). Assessments will be performed at baseline, and at Weeks 6 and 10.
9. Progression-free survival (PFS) and OS in patients with lung cancer. PFS is defined as the time from randomization to the first documented disease progression, based on the Response Evaluation Criteria in Solid Tumors (RECIST) version 1.1 (Bogaerts et al., 2009), or death from any cause, whichever occurs first. OS is defined as the time from randomization to death from any cause.

#### 2.6.3 Exploratory outcomes

To systematically investigate the potential mechanisms of action of MSNP granules, biological samples will be collected for analysis. At each study center, the first 20 participants enrolled in each stratification group (with or without concomitant paroxetine use) will be selected, yielding a total of 40 participants per center for mechanistic assessments. Peripheral blood and fecal samples will be obtained at baseline and at Week 6. Fasting venous blood samples will be drawn from the antecubital vein between 8:00 and 10:00 a.m. After standing at room temperature for 1 h, the samples will be centrifuged at 3,000 rpm for 1 min to obtain the supernatant. This will be followed by a second centrifugation at 12,000 rpm for 10 min at 4 °C to isolate serum (Wang et al., 2022). Fecal samples will be collected immediately after morning defecation using sterile containers. All serum and fecal specimens will be stored at −80 °C until further analysis. The following mechanistic indicators will be assessed:

1. Serum inflammatory markers, including interleukin-1β (IL-1β), interleukin-6 (IL-6), C-reactive protein (CRP), and tumor necrosis factor-α (TNF-α), measured using enzyme-linked immunosorbent assay (ELISA).
2. Serum hormones associated with the HPA axis, including adrenocorticotropic hormone (ACTH), corticotropin-releasing hormone (CRH), and cortisol (CORT), also measured using ELISA.
3. Serum neurotransmitters, including dopamine (DA), norepinephrine (NE), and 5-hydroxytryptamine (5-HT), quantified using high-performance liquid chromatography with electrochemical detection.
4. Serum BDNF, measured using ELISA.
5. Serum metabolomic profiles, analyzed through non-targeted liquid chromatography–mass spectrometry.
6. Gut microbiota composition, assessed via 16S rRNA gene sequencing.

### 2.7 Safety assessment

Throughout the study period, adverse events (AEs) will be monitored, documented, and managed in accordance with the International Conference on Harmonization-Good Clinical Practice (ICH-GCP) guidelines (Zemsi et al., 2024). AEs are defined as any unfavorable medical occurrence in a participant during the study, including symptoms, signs, diseases, or abnormalities in laboratory test results. Laboratory safety assessments will include routine blood tests and biochemical parameters (hepatic function, renal function, and electrolytes), conducted at baseline and at Weeks 2, 4, 6, and 10. Twelve-lead electrocardiograms will be performed at baseline, Week 4, and Week 10.

All AEs will be coded using the Medical Dictionary for Regulatory Activities (MedDRA) and graded according to the Common Terminology Criteria for Adverse Events (CTCAE), version 5.0, issued by the National Cancer Institute (Spencer et al., 2022). Events of Grade 3 or higher will be classified as serious adverse events (SAEs). All SAEs will be reported to the principal investigator and the institutional review board within 24 h of initial awareness. Appropriate medical management will be provided as required, and all AEs will be followed until resolution, stabilization, or clinical insignificance. Investigators may modify the dosage or discontinue administration of the investigational product at their discretion, based on the severity of the AE and its assessed causality.

The causal relationship between AEs and the investigational product will be assessed using the World Health Organization–Uppsala Monitoring Centre (WHO-UMC) system (Behera et al., 2018). Particular attention will be paid to known adverse reactions associated with paroxetine, such as fatigue, sweating, nausea, decreased appetite, somnolence, dizziness, insomnia, tremor, nervousness, and sexual dysfunction. For MSNP granules, gastrointestinal adverse events including nausea, diarrhea, and abdominal discomfort will be closely monitored. All AEs will be comprehensively documented, including onset time, duration, severity, management, clinical outcome, and their assessed relationship to the investigational product.

### 2.8 Participant timeline

**FIGURE 3:**
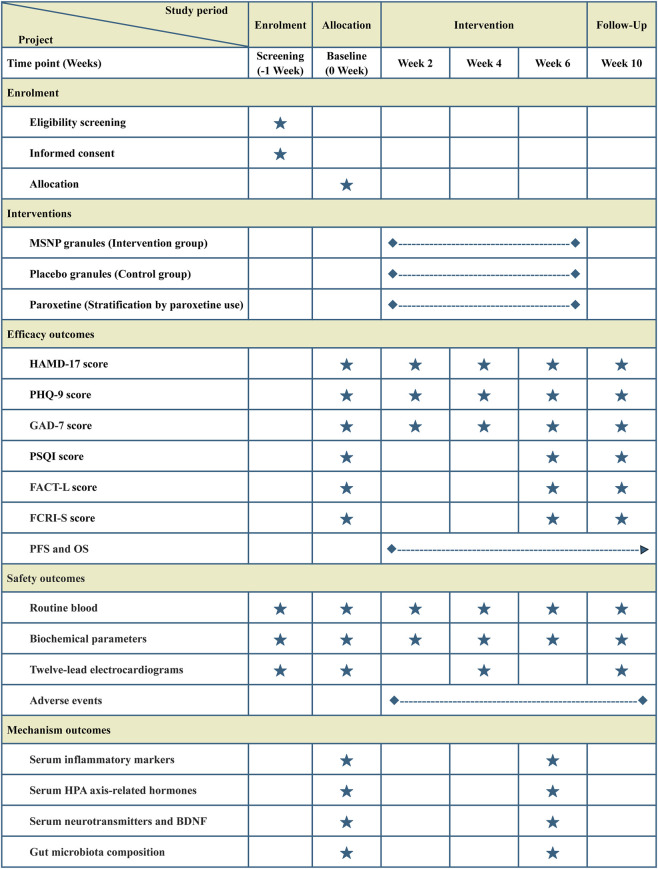
Schematic representation of participant inclusion, therapeutic interventions, and scheduled assessments. BDNF, brain-derived neurotrophic factor; FACT-L, Functional Assessment of Cancer Therapy–Lung; FCRI-S, Fear of Cancer Recurrence Inventory–Severity Subscale; GAD-7, Generalized Anxiety Disorder-7; HAMD-17, 17-item Hamilton Depression Rating Scale; HPA, hypothalamic-pituitary-adrenal; MSNP, modified Sini Powder; OS, overall survival; PHQ-9, Patient Health Questionnaire-9; PFS, progression-free survival; PSQI, Pittsburgh Sleep Quality Index.

The trial will last for 11 weeks, consisting of a 1-week screening period, a 6-week treatment phase, and a 4-week post-treatment follow-up. The timeline for participant enrollment, intervention delivery, and outcome measurements is outlined in Figure 3.

### 2.9 Sample size estimation

Previous clinical studies have shown that SNP, either alone or in combination with antidepressants, may help alleviate depressive symptoms. For example, a randomized, double-blind, placebo-controlled trial in breast cancer patients with mild to moderate depression showed that SNP significantly reduced HAMD-24 scores compared with placebo, with a greater reduction in the SNP group than in the placebo group (4.46 ± 4.403 vs. 0.66 ± 4.463, *P* < 0.001) (Hong et al., 2026). Another randomized trial, in which effectiveness was defined as a ≥25% reduction in the HAMD-17 total score from baseline, showed that SNP combined with fluoxetine improved the total effective rate in adolescents with depression compared with fluoxetine alone (95.12% vs. 77.50%, *P* < 0.05) (Zhang et al., 2023). These studies provide specific clinical evidence supporting the potential antidepressant effects of SNP.

The sample size was calculated based on the primary outcome, namely, the total effective rate at the end of the 6-week treatment period. Considering that concomitant use of antidepressants may affect treatment response, a stratified design was adopted, and sample sizes were estimated separately for the two strata: participants without paroxetine use and participants with paroxetine use. Because individual clinical trials differed from the present study in population characteristics, disease context, and intervention setting, the formal assumptions for sample size estimation were not directly derived from a single clinical trial. Instead, they were primarily based on the pooled effect estimates from a previous meta-analysis of traditional Chinese medicine for depression, which provided more robust parameter estimates (Wang et al., 2019).

According to this meta-analysis, in the non-paroxetine stratum, the expected total effective rates were 59.6% for MSNP alone and 37.0% for placebo alone, corresponding to a projected risk ratio of 1.61. In the paroxetine stratum, the expected total effective rates were 91.6% for MSNP plus paroxetine and 79.0% for placebo plus paroxetine, corresponding to a projected risk ratio of 1.16. Using the “Tests for Two Proportions” module in PASS version 21, with a two-sided alpha of 0.05, 80% power, equal allocation within each stratum, and an assumed dropout rate of 15%, the required sample sizes were 180 participants for the non-paroxetine stratum and 288 participants for the paroxetine stratum. Therefore, the final total sample size was set at 468 participants. In the non-paroxetine stratum, 90 participants will receive MSNP alone and 90 will receive placebo alone. In the paroxetine stratum, 144 participants will receive MSNP plus paroxetine and 144 will receive placebo plus paroxetine. Overall, 234 participants will receive MSNP-containing interventions (intervention group), and 234 participants will receive the corresponding placebo-containing control interventions (control group).

### 2.10 Recruitment strategies

Recruitment will follow a dual-pathway strategy combining proactive identification by the research team with voluntary engagement by potential participants. Investigators will identify potentially eligible patients through routine clinical pathways, including inpatient and outpatient oncology records, multidisciplinary tumor board documentation, and postoperative follow-up clinics. To improve recruitment efficiency, each study site will also conduct standardized online and offline recruitment activities through professional medical websites, hospital homepages, official social media accounts, recruitment posters, and informational brochures distributed in relevant departments such as thoracic surgery, oncology, and psychosomatic medicine. Potentially eligible individuals who express interest in participation will be contacted by trained research personnel and provided with detailed study information. After written informed consent is obtained, eligibility will be confirmed through standardized screening and baseline assessments. Recruitment progress will be regularly monitored across all sites, and additional site-level support or recruitment optimization measures will be implemented when necessary to ensure timely enrollment of the target sample size.

### 2.11 Randomization

A total of 468 participants will be enrolled from nine research centers, with 52 participants planned for each center. Randomization will be stratified by study center and by whether paroxetine is initiated after enrollment, with 20 participants in the non-paroxetine stratum and 32 participants in the paroxetine stratum at each center. Within each stratum, participants will be randomly assigned in a 1:1 ratio to receive either MSNP granules or placebo granules. The random allocation sequence will be generated by an independent statistician using computer software and restricted block randomization. Details of the block size and other restriction procedures will be kept in a separate confidential document unavailable to personnel responsible for enrollment or intervention assignment. The randomization sequence will correspond to sequentially numbered study medication codes. After eligibility confirmation and written informed consent, participants will receive the next available medication code according to their study center and paroxetine stratum.

### 2.12 Blinding

Trial participants, care providers, outcome assessors, and data analysts will remain blinded to treatment allocation throughout the study. Unblinding will only be permitted in cases of serious adverse events requiring urgent clinical intervention and must be approved by the principal investigator. In all other cases, treatment allocation will remain concealed until completion of the trial and finalization of data analysis. At the end of the 6-week treatment period and before unblinding, blinding assessment will be performed among participants, care providers, and outcome assessors. They will be asked to guess the treatment allocation as “MSNP,” “placebo,” or “uncertain” and to provide the reason for their guess where applicable. The results of the blinding assessment will be summarized descriptively to evaluate the success of blinding.

### 2.13 Data collection and management

Study data will be documented on customized, paper-based case report forms (CRFs) developed specifically for this trial. Information to be collected includes baseline demographic data and clinical characteristics (e.g., duration of depressive symptoms, depression severity, tumor stage, and time since surgery), treatment adherence, scheduled outcome evaluations, laboratory data, and safety-related events. At each site, trained research personnel will collect data in accordance with a standardized operating procedure manual to ensure procedural consistency. Prior to study initiation, all research staff will undergo centralized training covering CRF documentation, clinical scale scoring (e.g., HAMD-17, PHQ-9, GAD-7), adverse event logging, and source verification protocols. To encourage compliance and reduce missed follow-ups, participants will receive visit schedules in advance and be contacted regularly throughout the study.

Following data entry onto CRFs, trained staff will transcribe information into a centralized electronic data capture system with secure, role-based login credentials. The system features automated logic checks, completeness validation, timestamped audit trails, and alerts for anomalous or missing data. A dual-entry process will be implemented to minimize data discrepancies. Ongoing central monitoring will be conducted by the data management team, who will review data submissions, issue queries when necessary, and track resolution in accordance with pre-established data handling procedures. All data will be anonymized using coded identifiers, and identifying information will not be included in the analytical dataset. Original paper records will be retained in locked, access-restricted locations at each site. Encrypted digital data will be regularly backed up, and all trial records will be archived for at least 5 years post-completion. Data security and protocol compliance will be overseen by the institutional review boards of all participating centers.

### 2.14 Statistical analysis

All efficacy analyses will be performed according to the intention-to-treat (ITT) principle in all randomized participants. Safety analyses will be conducted in all participants who initiate protocol-defined treatment. Missing outcome data will be handled using multiple imputation under the missing-at-random assumption.

The primary outcome (total effective rate) will be analyzed using a log-binomial regression model to estimate the adjusted RR and corresponding 95% confidence interval (CI) between the intervention and control groups. The model will include study center and concomitant paroxetine use as covariates. An interaction term between treatment group and paroxetine use will be included to assess the potential moderating effect of antidepressant co-administration on the intervention effect. Sensitivity analyses will include a predefined per-protocol (PP) analysis to assess the robustness of the findings. The PP population will include participants who complete the entire treatment course without major protocol violations and have available primary outcome data. Prespecified subgroup analyses will be performed according to baseline sex (male vs. female), age (≤55 vs. >55 years), depression severity (mild vs. moderate), NSCLC stage (I vs. II vs. III), and KPS score (70–80 vs. 90–100).

For other outcomes, repeated-measures continuous variables such as HAMD-17, PHQ-9, and GAD-7 scores will be analyzed using linear mixed-effects models to evaluate longitudinal changes from baseline between groups. The models will include group, time, group-by-time interaction, and baseline score as fixed effects, and participant and study center as random effects. For single-timepoint continuous variables such as inflammatory markers and neurotransmitters, between-group comparisons will be conducted using independent-samples t-tests or Mann–Whitney U tests, depending on data distribution. For time-to-event outcomes including PFS and OS, Cox proportional hazards models will be used to estimate hazard ratios and 95% CIs, adjusting for study center and paroxetine use. For categorical outcomes such as AE incidence and depression remission rate, group comparisons will be conducted using the chi-square test or Fisher’s exact test, as appropriate.

All statistical analyses will be performed using SPSS version 26.0 and R version 4.3.1. A two-sided p-value ≤ 0.05 will be considered statistically significant for the primary outcome. No adjustment for multiplicity is planned for secondary outcomes, and these analyses will be regarded as exploratory. No interim analyses are planned for this trial.

### 2.15 Trial status

Participant recruitment for this clinical trial is anticipated to commence in June 2026.

### 2.16 Trial termination criteria

The trial may be suspended or terminated early if any of the following circumstances occur: (1) unexpected safety risks judged to be related to the study intervention and sufficient to affect participant safety; (2) an unexpectedly high incidence of serious AEs indicating that the overall risk–benefit profile is no longer acceptable; (3) major protocol violations or serious deficiencies in trial conduct that may compromise participant safety, data integrity, or the validity of trial results; (4) failure to achieve adequate recruitment or follow-up despite corrective measures, making the study objectives unattainable; or (5) a decision by the ethics committee, regulatory authority, or data monitoring committee indicating that continuation of the trial is inappropriate. Any decision to suspend or terminate the trial will be documented and reported to relevant parties, and appropriate safety follow-up will be provided for enrolled participants.

## 3 Discussion

This study is a nationwide, multicenter, randomized, double-blind, placebo-controlled clinical trial designed to systematically evaluate the efficacy, safety, and potential mechanisms of MSNP in the treatment of postoperative mild-to-moderate depression in patients with NSCLC. The trial is based on a rigorous methodological design, integrating multidimensional efficacy assessments and omics-based mechanistic analyses. It aims to address the current lack of high-quality clinical evidence for TCM interventions in depression.

Grounded in real-world clinical practice, this study adopts a design that ensures both scientific rigor and clinical applicability. First, it employs a strict randomized, double-blind, placebo-controlled design and is conducted collaboratively across nine research centers in different provinces of China, ensuring strong external validity and generalizability. Second, the study stratifies participants based on concomitant paroxetine use, with the aim of achieving two key objectives. The first is to evaluate the efficacy and safety of MSNP monotherapy as a viable alternative for patients who are unwilling or unable to tolerate antidepressants. The second is to explore the potential synergistic effects or mitigation of side effects when MSNP is combined with standard pharmacotherapy. This dual-scenario design enables a comprehensive appraisal of MSNP’s clinical value in personalized treatment strategies. In addition, the study includes multidimensional efficacy endpoints covering depression, anxiety, sleep disturbance, fear of recurrence, quality of life, and cancer survival. Multiple timepoint assessments are used to capture the psychological and physiological effects of MSNP in a comprehensive manner.

The pathogenesis of depression is a highly complex biological process involving several key mechanisms, including inflammation, HPA axis dysregulation, neurotransmitter imbalance, reduced BDNF levels, gut microbiota disturbances, and metabolic abnormalities. TCM is characterized by multi-component, multi-target, and multi-pathway pharmacological properties, which align well with the multifactorial and systemic pathology of depression. MSNP may exert its effects by simultaneously regulating multiple biological pathways involved in the disease process (Li et al., 2020). Studies have shown that SNP may prevent reserpine-induced depression in rats by reducing IL-1β, IL-6, and TNF-α levels in serum and hippocampus, thereby alleviating inflammation (Zong et al., 2020). Other studies indicate that SNP may produce antidepressant effects by increasing the expression of 5-HT1A receptors and BDNF in the hippocampus (Cao et al., 2019). Chaihu-Shu-Gan-San (CSGS) is another classical antidepressant TCM formula with a composition similar to MSNP and sharing the treatment principle of soothing the liver and regulating qi. Multiple animal studies suggest that CSGS may alleviate depressive symptoms through several biological pathways (Yu et al., 2020; Ma et al., 2022; Han et al., 2021), such as modulating gut microbiota (e.g., increasing *Parabacteroides distasonis*), regulating blood metabolites (e.g., elevating bile acid levels), regulating neurotransmitters (e.g., increasing hippocampal levels of DA, NE, and 5-HT), suppressing HPA axis hyperactivity (e.g., reducing serum CORT), and attenuating inflammatory responses (e.g., lowering serum IL-1β and IL-6). Based on this preliminary evidence, the present study will apply multi-omics approaches to comprehensively investigate the biological mechanisms of MSNP, focusing on inflammation, neurotransmitters, HPA axis function, BDNF levels, gut microbiota composition, and serum metabolic profiles.

Several potential limitations should be noted. First, the study does not include a head-to-head comparison between MSNP and standard antidepressants. If the results are promising, future trials may consider such comparative studies. Second, this trial focuses on patients with postoperative depression after NSCLC surgery. Future studies could expand to include other cancer types or general depressive populations to further examine the generalizability and clinical applicability of MSNP. Third, current mechanistic investigations are primarily based on peripheral biomarkers. Future research may incorporate neuroimaging techniques, such as functional magnetic resonance imaging and positron emission tomography, to assess MSNP’s impact on brain network activity and further elucidate its central mechanisms of action.

In summary, by integrating a rigorously designed randomized controlled trial with systematic omics-based analyses, this study aims to construct a coherent evidence chain linking clinical efficacy with biological mechanisms. The findings are expected to support the clinical application of MSNP in the treatment of postoperative mild-to-moderate depression in NSCLC patients and contribute to the modernization and translational development of TCM interventions for cancer-related psychological disorders.
