# Supplementary material for: Modified sini powder for the management of postoperative depression in non-small cell lung cancer patients: a multicenter, randomized, double-blind, placebo-controlled trial protocol
Source: Front Pharmacol. 2026 Jun 19;17:1805554. doi: 10.3389/fphar.2026.1805554 (PMC13327883; doi:10.3389/fphar.2026.1805554)
Supplement: Supplementary file 1 [file DataSheet1.zip › Supplementary materials/Supplementary material 2.pdf]

Ultra-Performance Liquid Chromatography (UPLC) Fingerprint Analysis of Modified Sini Powder (MSNP) Granules

1. Materials

1.1 Sample Source: Five batches of MSNP granules (labeled S1, S2, S3, S4, and S5).

1.2 Reagents: Methanol (chromatographic grade, Sigma-WXBF6140V, Chengdu Zhongjinlong Technology Development Co., Ltd.), ammonium formate (chromatographic grade, 226467, Fisher Scientific), purified water (Wahaha).

1.3 Reference Standards: Supplied by Chengdu Gelipu Biotechnology Co., Ltd. Batch information: Synephrine (25020809, purity 99.97%), Paeoniflorin (25120505, purity 99.49%), Ammonium Glycyrrhizinate (25050611, expressed as glycyrrhizic acid, purity 97.20%), Saikosaponin B2 (23011306, purity 98.69%).

2. Instruments and Models

2.1 UPLC System: Waters Arc PREMIER (Waters Corporation, USA).

2.2 Analytical Balance: SQP-QUINTIX125D-1CN (Sartorius Scientific Instruments Co., Ltd., readability: 0.01 mg).

3. Instrumental Conditions

Chromatographic Column: ChromCore C18 (4.6 × 250 mm, 5 μm).

Mobile Phase: Methanol (A) and 10 mmol/L ammonium formate solution (B).

Detection Wavelength: 220 nm (0–11 min), 250 nm (11–60 min).

Column Temperature: 25 °C.

Flow Rate: 0.8 mL/min.

Gradient elution program as follows:

Time (min)	Mobile Phase A (%)	Mobile Phase B (%)
0-9	3	97
9-25	3→40	97→60
25-50	40→85	60→15
50-60	85	15

4. Preparation of Mixed Standard Solution

4.1.1 Stock Solutions of Reference Standards: Precisely weighed amounts of synephrine (10.44 mg), paeoniflorin (10.58 mg), and ammonium glycyrrhizinate (13.33 mg) were separately dissolved in 10 mL volumetric flasks; saikosaponin B2 (10.53 mg) was dissolved in a 25 mL volumetric flask. Each was diluted to volume with 10% methanol.

4.1.2 Mixed Reference Solution: Accurately transferred 1 mL of synephrine, 3 mL of paeoniflorin, 3 mL of ammonium glycyrrhizinate, and 0.2 mL of saikosaponin B2 stock solutions into a 10 mL volumetric flask, diluted to volume with 10% methanol, and mixed well.

4.1.3 Mixed Standard Solution: Accurately transferred 1.6 mL of the mixed reference solution into a 5 mL volumetric flask, diluted to volume with 10% methanol, and mixed thoroughly.

5. Preparation of Test Sample Solution

Approximately 0.5 g of MSNP granules was accurately weighed into a stoppered conical flask, and 50 mL of 10% methanol was precisely added. After sealing and

weighing, the mixture was sonicated for 30 min, cooled to room temperature, and re-weighed. The weight loss was compensated with 10% methanol. The solution was shaken well, filtered, and the subsequent filtrate was collected as the test sample solution.

6. Results

The HPLC fingerprints of five batches of MSNP granules were studied, and the relative retention time of each chromatographic peak was determined using a mixed standard solution as the reference (**Figure 1**). Similarity evaluation was conducted using the Similarity Evaluation System for Chromatographic Fingerprint of Traditional Chinese Medicine (version 2012.130723). The results indicated that, when compared with the reference fingerprint (R), all similarity values exceeded 0.9 (**Figure 2** and **Table 1**).

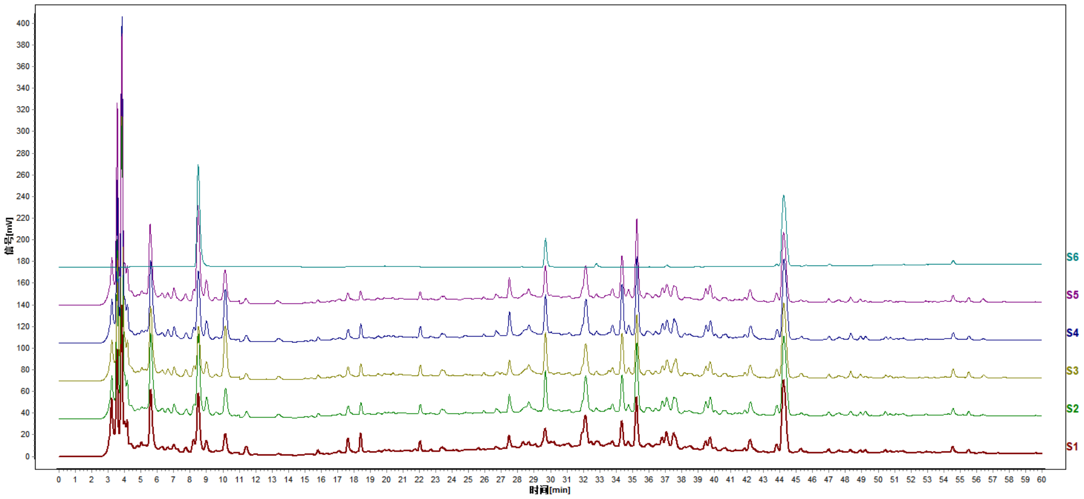

Figure 1. UPLC chromatograms of the mixed standard solution (S6) and five batches of MSNP granules (S1–S5).

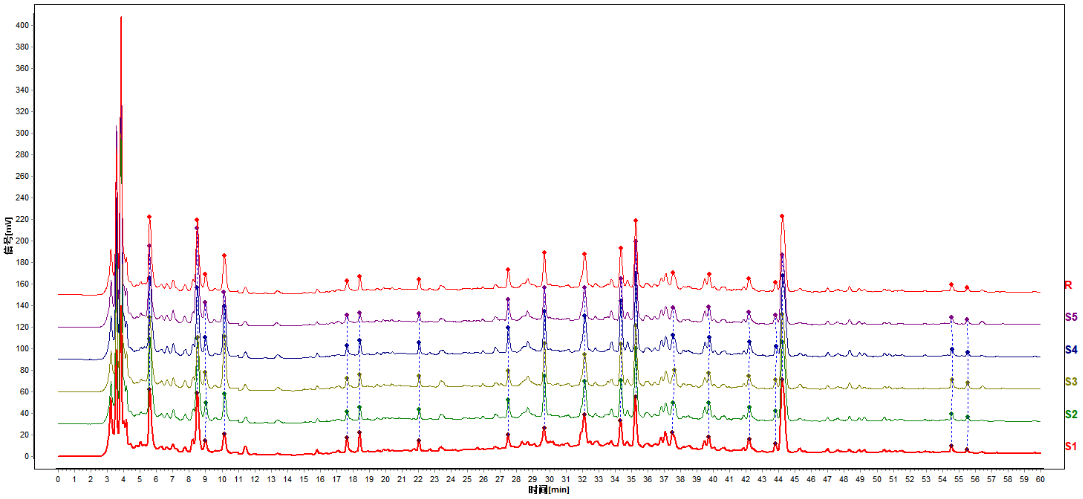

Figure 2. UPLC fingerprint chromatograms of the reference fingerprint (R) and five batches of MSNP granules (S1–S5).

Table 1. Similarity Matrix of Chromatographic Fingerprints

	S1	S2	S3	S4	S5	R
S1	1.000	0.992	0.968	0.979	0.969	0.990
S2	0.992	1.000	0.972	0.986	0.988	0.996
S3	0.968	0.972	1.000	0.995	0.954	0.986
S4	0.979	0.986	0.995	1.000	0.975	0.996
S5	0.969	0.988	0.954	0.975	1.000	0.987
R	0.990	0.996	0.986	0.996	0.987	1.000

Quantification of Marker Compounds in Modified Sini Powder (MSNP) Granules

1. Materials

1.1 **Sample Source:** Five batches of MSNP granules (labeled S1, S2, S3, S4, and S5).

1.2 **Reagents:** Methanol (chromatographic grade, Sigma-WXBF6140V, Chengdu Zhongjinlong Technology Development Co., Ltd.), ammonium formate (chromatographic grade, 226467, Fisher Scientific), purified water (Wahaha).

1.3 **Reference Standards:** Supplied by Chengdu Gelipu Biotechnology Co., Ltd. Batch information: Synephrine (25020809, purity 99.97%), Paeoniflorin (25120505, purity 99.49%), Ammonium Glycyrrhizinate (25050611, expressed as glycyrrhizic acid, purity 97.20%), Saikosaponin B2 (23011306, purity 98.69%).

2. Instruments and Models

2.1 **HPLC System:** Waters Arc PREMIER (Waters Corporation, USA).

2.2 **Analytical Balance:** SQP-QUINTIX125D-1CN (Sartorius Scientific Instruments Co., Ltd., readability: 0.01 mg).

3. Instrumental Conditions

Chromatographic Column: ChromCore C18 (4.6 × 250 mm, 5 μm).

Mobile Phase: Methanol (A) and 10 mmol/L ammonium formate solution (B).

Detection Wavelength: 220 nm (0–11 min), 250 nm (11–60 min).

Column Temperature: 25 °C.

Flow Rate: 0.8 mL/min.

Gradient elution program as follows:

Time (min)	Mobile Phase A (%)	Mobile Phase B (%)
0-9	3	97
9-25	3→40	97→60
25-50	40→85	60→15
50-60	85	15

4. Method Validation

4.1 **Preparation of Standard Solutions**

4.1.1 **Stock Solutions of Reference Standards:** Precisely weighed amounts of synephrine (10.44 mg), paeoniflorin (10.58 mg), and ammonium glycyrrhizinate (13.33 mg) were separately dissolved in 10 mL volumetric flasks; saikosaponin B2 (10.53 mg) was dissolved in a 25 mL volumetric flask. Each was diluted to volume with 10% methanol.

4.1.2 **Second Stock Solution:** Accurately weighed synephrine (26.07 mg) was dissolved in a 10 mL volumetric flask, and saikosaponin B2 (19.04 mg) was dissolved in a 20 mL volumetric flask. Both were diluted to volume with 10% methanol.

4.2 **Linearity and Range**

A mixed reference solution was prepared by accurately transferring 1 mL of synephrine, 3 mL of paeoniflorin, 3 mL of ammonium glycyrrhizinate, and 0.2 mL of saikosaponin B2 from Stock Solution 1 into a 10 mL volumetric flask and diluting to volume with 10% methanol.

A series of six working solutions with different concentrations were prepared by sequential dilution of the mixed reference solution. Each solution was injected (10 μL)

in triplicate under the chromatographic conditions described in Section 3. The peak area (y) was plotted against concentration (x, μg/mL), and linear regression analysis was performed. All compounds showed good linearity within their respective ranges (**Table 2**).

Table 2. Linear Regression Equations, Correlation Coefficients, and Linear Ranges

Analyte	Regression Equation	R^2	Linear Range (μg/mL)
Synephrine	y = 36619.2066 x - 1800.9713	0.9999	4.675716864~58.4464608
Paeoniflorin	y = 2716.1932 x + 3640.8122	0.9999	14.14700045~176.8375056
Glycyrrhizic Acid	y = 9921.2744 x + 12554.1833	1.0000	17.41388544~217.673568
Saikosaponin B2	y = 16617.6851 x + 3848.6999	0.9995	0.372451323~4.655641536

4.3 **Precision**

The intermediate precision was assessed by six consecutive injections of the same working solution (dilution 2 from Section 4.2). The relative standard deviation (RSD) of the peak areas was calculated (**Table 3**).

4.4 **Repeatability**

Six independent sample solutions were prepared from the same batch (S1) according to the procedure described in Section 5. Each solution was injected, and the content of

each marker compound was determined. The RSD of the contents was calculated (**Table 3**).

4.5 **Recovery**

Accurately weighed amounts of the sample (approximately 0.25 g, with known content) were spiked with known quantities of the four reference compounds. Six spiked samples were prepared and analyzed following the sample preparation procedure. The average recovery and RSD were calculated for each compound (**Table 3**).

4.6 **Stability**

A single test sample solution was stored at room temperature and analyzed at 0, 6, 12, 24, and 48 h. The RSD of the peak areas for each marker compound was calculated (**Table 3**).

Table 3. The precision, repeatability, recovery, and stability data for the four compounds

Analyte	Precision	Repeatability	Recovery rate		Stability
	RSD (%)	RSD (%)	Mean (%)	RSD (%)	RSD (%)
Synephrine	0.1	2.4	101	0.9	1.6
Paeoniflorin	0.1	1.1	97	0.9	0.6
Glycyrrhizic Acid	0.1	1.6	98	0.5	1.7
Saikosaponin B2	0.8	3.2	100	0.9	0.5

4.7 **Specificity**

Chromatograms of a blank solvent, the mixed standard solution, and the test sample solution were compared. No interfering peaks were observed at the retention times of the target analytes, confirming the method's specificity (**Figure 3**).

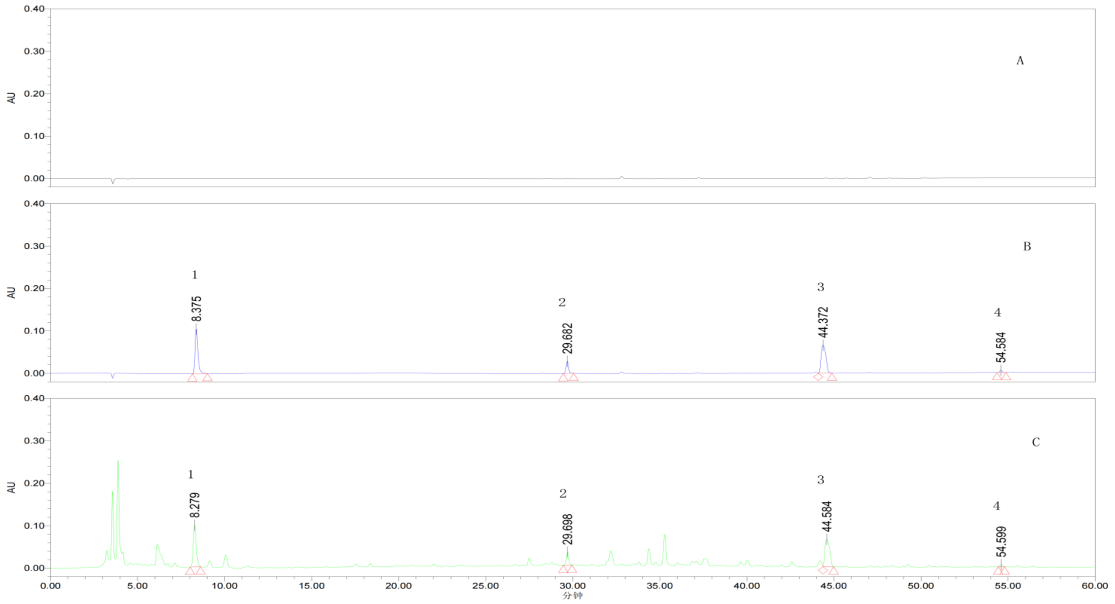

Figure 3. Representative HPLC Chromatograms. (A: Blank solvent; B: Mixed standard solution; C: Test sample solution;1: Synephrine; 2: Paeoniflorin; 3: Glycyrrhizic Acid; 4: Saikosaponin B2)

5. Preparation of Test Sample Solution

Approximately 0.5 g of MSNP granules was accurately weighed into a stoppered conical flask, and 50 mL of 10% methanol was precisely added. After sealing and weighing, the mixture was sonicated for 30 min, cooled to room temperature, and re-weighed. The weight loss was compensated with 10% methanol. The solution was shaken well, filtered, and the subsequent filtrate was collected as the test sample solution.

6. Quantitative Analysis of Samples

The test sample solutions of all five batches (S1–S5) were prepared in triplicate according to Section 5 and analyzed under the chromatographic conditions specified in Section 3. The content of each marker compound was calculated using the corresponding calibration curve. The results are presented in **Table 4**.

Table 4. Content of Marker Compounds in Five Batches of MSNP Granules

Batch	Content (mg/g)			
	Synephrine	Paeoniflorin	Glycyrrhizic Acid	Saikosaponin B2
S1	1.87	7.70	11.48	0.27
S2	2.71	13.39	13.17	0.28
S3	1.60	13.40	11.47	0.38
S4	2.23	13.14	13.38	0.29
S5	3.14	10.27	11.34	0.28
