# Supplementary material for: Modified sini powder for the management of postoperative depression in non-small cell lung cancer patients: a multicenter, randomized, double-blind, placebo-controlled trial protocol
Source: Front Pharmacol. 2026 Jun 19;17:1805554. doi: 10.3389/fphar.2026.1805554 (PMC13327883; doi:10.3389/fphar.2026.1805554)
Supplement: Supplementary file 1 [file DataSheet1.zip › Supplementary materials/Supplementary material 3.pdf]

检 验 报 告 书

文件编号：XLS-QC-MP-0007-OR01(00)

报告书编号： C70B003—25006

**品　名**	白芍配方颗粒	**规　格**	100g/袋（每1g配方颗粒相当于饮片4.5g）
**批　号**	25010366	**请验部门**	固体制剂车间
**代 表 量**	20179 袋	**收样日期**	2025年02月03日
**来　源**	包装工序	**报告日期**	2025年02月11日
**检验依据**	《白芍配方颗粒国家药品标准（YBZ-PFKL-2021002）》		

**检验项目**	**检验标准**	**检验结果**
**性　状**	本品应为黄白色至黄棕色的颗粒；气微，味苦、微酸。	符合规定
**薄层鉴别**	供试品色谱中，在与白芍对照药材和芍药苷对照品色谱相应的位置上，应显相同颜色的斑点。	符合规定
**特征图谱**	应符合规定	符合规定
**检　查**		
粒　度	不能通过一号筛与能通过五号筛的总和应≤15%	4.3%
水　分	≤8.0%	3.0%
溶化性	应全部溶化或轻微浑浊，不得有焦屑或异物	符合规定
装　量	平均装量应≥100g，每袋装量应≥97g	符合规定
硫熏检查	应符合规定	符合规定
**浸出物**	乙醇热浸出物≥35.0%	52.3%
**含量测定**	芍药苷应为65.0mg/g～137.0mg/g	79.4mg/g
重金属及有害元素（*）	铅≤5mg/kg	0.1mg/kg
	镉≤1mg/kg	0.03mg/kg
	砷≤2mg/kg	0.4mg/kg
	汞≤0.2mg/kg	低于0.01673mg/kg
	铜≤20mg/kg	6.4mg/kg
**微生物限度检查**		
需氧菌总数	≤10^3 CFU/克（最大可接受2000 CFU/克）	<20 CFU/克
霉菌和酵母菌总数	≤10^2 CFU/克（最大可接受200 CFU/克）	<20 CFU/克
大肠埃希菌	不得检出	未检出

备注：带（*）项目结果为引用结果。

备注：重金属 检测
ICP-MS检出限（单位mg/kg）：Pb 0.04197　Cd 0.0071　As 0.01279　Hg 0.01673　Cu 0.03591
当检测结果低于上述值时以低于上述数值出具报告

以下空白

结论：本品按《白芍配方颗粒国家药品标准（YBZ-PFKL-2021002）》检验，结果符合规定。

（印章：四川新绿色药业科技发展有限公司 质检专用章）

批准人：[signature] 2025-02-11　　复核人：[signature] 2025.02.11　　编制人：[signature] 2025-02-11

声明：1、本报告检验结果仅对送检样品负责；
2、对本报告若有异议，应于收到报告之日起七个工作日内向本中心提出，逾期将自动视为承认本检测报告。

检测中心地址：四川省彭州市致和镇东河东路279号　　电话号码：028-83888888

成品检验报告书

文件编号：XLS-QC-MP-0007-0R03(00)

报告书编号：C70Z067—25004

品　　名	枳实（酸橙）配方颗粒	规　　格	100g/袋（每1g配方颗粒相当于饮片3.3g）
批　　号	25080035	产　　地	湖南
代 表 量	10991 袋	收样日期	2025年08月20日
来　　源	包装工序	报告日期	2025年08月26日
检验依据	《枳实（酸橙）配方颗粒国家药品标准（YBZ-PFKL-2021152）》		

检验项目	检验标准	检验结果
性　状	本品应为浅黄色至浅棕色的颗粒；气微，味苦、涩。	符合规定
薄层鉴别	供试品色谱中，在与枳实（酸橙）对照药材色谱和辛弗林对照品色谱相应的位置上，应显相同颜色的斑点。	符合规定
特征图谱	应符合规定	符合规定
检　查		
粒　度	不能通过一号筛与能通过五号筛的总和应≤15%	1.1%
水　分	≤8.0%	3.3%
溶化性	应全部溶化或轻微浑浊，不得有焦屑或异物	符合规定
装　量	平均装量应≥100g，每袋装量应≥97g	符合规定
浸出物	乙醇热浸出物≥21.0%	53.0%
含量测定	辛弗林应为5.5mg/g～17.5mg/g	14.2mg/g
微生物限度检查		
需氧菌总数	≤10^3 CFU/克（最大可接受2000 CFU/克）	＜10 CFU/克
霉菌和酵母菌总数	≤10^2 CFU/克（最大可接受200 CFU/克）	＜10 CFU/克
大肠埃希菌	不得检出	未检出

以下空白

结论：本品按《枳实（酸橙）配方颗粒国家药品标准（YBZ-PFKL-2021152）》检验，结果符合规定。

批准人：（签名）2025.08.26　　复核人：（签名）2025.08.26　　编制人：（签名）2025.08.26

（印章：四川新绿色药业科技发展有限公司 质检专用章）

声明：1、本报告检验结果仅对送检样品负责；
2、对本报告若有异议，应于收到报告之日起七个工作日内向本中心提出，逾期将自动视为承认本检测报告。

检测中心地址：四川省彭州市致和镇东河东路279号　　电话号码：028-83888888

成品检验报告书

文件编号：XLS-QC-MP-0007-OR03(00)

报告书编号： C70Z040—25014

**品　　名**	酸枣仁配方颗粒	**规　　格**	100g/袋（每1g配方颗粒相当于饮片4g）
**批　　号**	25080138	**产　　地**	河北
**代 表 量**	537 袋	**收样日期**	2025年08月14日
**来　　源**	包装工序	**报告日期**	2025年08月26日
**检验依据**	《酸枣仁配方颗粒国家药品标准（YBZ-PFKL-2021191）》		

**检验项目**	**检验标准**	**检验结果**
**性　状**	本品应为浅棕黄色至黄棕色的颗粒；气微香，味微苦、微酸。	符合规定
**薄层鉴别**	供试品色谱中，在与酸枣仁对照药材色谱和取酸枣仁皂苷A对照品、酸枣仁皂苷B对照品色谱相应的位置上，应显相同颜色的斑点。	符合规定
**特征图谱**	应符合规定	符合规定
**检　查**		
粒　度	不能通过一号筛与能通过五号筛的总和应≤15%	3.0%
水　分	≤8.0%	3.5%
溶化性	应全部溶化或轻微浑浊，不得有焦屑或异物	符合规定
装　量	平均装量应≥100g，每袋装量应≥97g	符合规定
**浸出物**	乙醇热浸出物≥14.0%	44.7%
**含量测定**	酸枣仁皂苷A应为0.70mg/g～2.8mg/g	1.41mg/g
	斯皮诺素应为1.8mg/g～5.0mg/g	2.7mg/g
**微生物限度检查**		
需氧菌总数	≤10^3 CFU/克（最大可接受2000 CFU/克）	＜10 CFU/克
霉菌和酵母菌总数	≤10^2 CFU/克（最大可接受200 CFU/克）	＜10 CFU/克
大肠埃希菌	不得检出	未检出

以下空白

结论：本品按《酸枣仁配方颗粒国家药品标准（YBZ-PFKL-2021191）》检验，结果符合规定。

批准人：[illegible] 2025.08.26　　复核人：[illegible] 2025.08.26　　编制人：[illegible] 2025.08.26

声明：1、本报告检验结果仅对送检样品负责；
2、对本报告若有异议，应于收到报告之日起七个工作日内向本中心提出，逾期将自动视为承认本检测报告。

检测中心地址：四川省彭州市致和镇东河东路279号　　电话号码：028-83888888

成品检验报告书

文件编号：XLS-QC-MP-0007-OR03(00)

报告书编号： C70G068—25014

品　　名	甘草（甘草）配方颗粒	规　　格	100g/袋（每1g配方颗粒相当于饮片3g）
批　　号	25080150	产　　地	新疆
代表量	12134 袋	收样日期	2025年09月03日
来　　源	包装工序	报告日期	2025年09月22日
检验依据	《甘草（甘草）配方颗粒国家药品标准（YBZ-PFKL-2021049）》		

检验项目	检验标准	检验结果
性　状	本品应为黄色至棕黄色的颗粒；气微，味甜而特异。	符合规定
薄层鉴别	供试品色谱中，在与甘草（甘草）对照药材色谱和甘草酸铵对照品色谱相应的位置上，应显相同颜色的荧光斑点。	符合规定
特征图谱	应符合规定	符合规定
检　查		
粒　度	不能通过一号筛与能通过五号筛的总和应≤15%	2.3%
水　分	≤8.0%	2.1%
溶化性	应全部溶化或轻微浑浊，不得有焦屑或异物	符合规定
装　量	平均装量应≥100g，每袋装量应≥97g	符合规定
浸出物	乙醇热浸出物≥32.0%	40.8%
含量测定	甘草苷应为15.0mg/g～35.0mg/g	20.3mg/g
	甘草酸应为29.0mg/g～80.0mg/g	58.3mg/g
重金属及有害元素	铅≤5mg/kg	0.2mg/kg
	镉≤1mg/kg	0.007mg/kg
	砷≤2mg/kg	0.4mg/kg
	汞≤0.2mg/kg	低于0.01673mg/kg
	铜≤20mg/kg	4.5mg/kg
有机氯农药残留量	五氯硝基苯≤0.1mg/kg	低于0.000516mg/kg
微生物限度检查		
需氧菌总数	$\leq 10^{3}$ CFU/克（最大可接受2000 CFU/克）	<10 CFU/克
霉菌和酵母菌总数	$\leq 10^{2}$ CFU/克（最大可接受200 CFU/克）	<10 CFU/克
大肠埃希菌	不得检出	未检出

备注：重金属 检测
ICP-MS检出限（单位mg/kg）：Pb 0.04197　Cd 0.0071　As 0.01279　Hg 0.01673　Cu 0.03591
当检测结果低于上述值时以低于上述数值出具报告

备注：有机氯农药残 检测
有机氯农药残检出限（单位mg/kg）：五氯硝基苯0.000516
当检测结果低于上述值时以低于上述数值出具报告

以下空白

四川新绿色药业科技发展有限公司 质检专用章

结论：本品按《甘草（甘草）配方颗粒国家药品标准（YBZ-PFKL-2021049）》检验，结果符合规定。

批准人：[signature] 2025.09.22　　复核人：[signature] 2025.09.22　　编制人：[signature] 2025.09.22

声明：1、本报告检验结果仅对送检样品负责；
2、对本报告若有异议，应于收到报告之日起七个工作日内向本中心提出，逾期将自动视为承认本检测报告。

检测中心地址：四川省彭州市致和镇东河东路279号　　电话号码：028-83888888

成品检验报告书

文件编号：XLS-QC-MP-0007-OR03(00)

报告书编号： C70H016—25007

品　名	合欢皮配方颗粒	规　格	100g/袋（每1g配方颗粒相当于饮片10g）
批　号	25110046	产　地	河南
代表量	3755 袋	收样日期	2025年11月07日
来　源	包装工序	报告日期	2025年11月14日
检验依据	《合欢皮配方颗粒国家药品标准（YBZ-PFKL-2021056）》		

检验项目	检验标准	检验结果
性　状	本品应为黄色至黄棕色的颗粒；气微香，味淡、微涩、稍刺舌，而后喉头有不适感。	符合规定
薄层鉴别	供试品色谱中，在与合欢皮对照药材和（一）-丁香树脂酚-4-O-β-D-呋喃芹糖基-（1→2）-β-D-吡喃葡萄糖苷对照品色谱相应的位置上，应显相同颜色的斑点。	符合规定
特征图谱	应符合规定	符合规定
检　查		
粒　度	不能通过一号筛与能通过五号筛的总和应≤15%	2.0%
水　分	≤8.0%	4.4%
溶化性	应全部溶化或轻微浑浊，不得有焦屑或异物	符合规定
装　量	平均装量应≥100g，每袋装量应≥97g	符合规定
浸出物	乙醇热浸出物≥45.0%	57.6%
含量测定	（一）-丁香树脂酚-4-O-β-D-呋喃芹糖基-（1→2）-β-D-吡喃葡萄糖苷应为3.0mg/g～9.0mg/g	4.8mg/g
微生物限度检查		
需氧菌总数	≤10^3 CFU/克（最大可接受2000 CFU/克）	＜10 CFU/克
霉菌和酵母菌总数	≤10^2 CFU/克（最大可接受200 CFU/克）	＜10 CFU/克
大肠埃希菌	不得检出	未检出

以下空白

结论：本品按《合欢皮配方颗粒国家药品标准（YBZ-PFKL-2021056）》检验，结果符合规定。

四川新绿色药业科技发展有限公司 质检专用章

批准人：[signature] 2025.11.14　　复核人：[signature] 2025.11.14　　编制人：[signature] 2025.11.14

声明：1、本报告检验结果仅对送检样品负责；
2、对本报告若有异议，应于收到报告之日起七个工作日内向本中心提出，逾期将自动视为承认本检测报告。

检测中心地址：四川省彭州市致和镇东河东路279号　　电话号码： 028-83888888

成 品 检 验 报 告 书

文件编号：XLS-QC-MP-0007-0R03(00)

报告书编号： C70C087—25008

**品　　名**	醋北柴胡配方颗粒	**规　　格**	100g/袋（每1g配方颗粒相当于饮片3.5g）
**批　　号**	25110219	**产　　地**	山西
**代 表 量**	11475 袋	**收样日期**	2025年11月25日
**来　　源**	包装工序	**报告日期**	2025年12月14日
**检验依据**	《醋北柴胡配方颗粒国家药品标准（YBZ-PFKL-2021030）》		

**检验项目**	**检验标准**	**检验结果**
**性　状**	本品应为黄色至黄棕色颗粒；气微，味微苦。	符合规定
**薄层鉴别**	供试品色谱中，在与柴胡（北柴胡）对照药材和柴胡皂苷a对照品色谱相应位置上，分别应显相同颜色的斑点或荧光斑点。	符合规定
**特征图谱**	应符合规定	符合规定
**检　查**		
粒　度	不能通过一号筛与能通过五号筛的总和应≤15%	1.8%
水　分	≤8.0%	4.2%
溶化性	应全部溶化，允许有轻微浑浊，不得有焦屑等异物	符合规定
装　量	平均装量应≥100g，每袋装量应≥97g	符合规定
**浸出物**	乙醇热浸出物≥17.0%	34.6%
**含量测定**	柴胡皂苷a应为1.10mg/g～4.00mg/g	1.35mg/g
**微生物限度检查**		
需氧菌总数	≤10^3 CFU/克（最大可接受2000 CFU/克）	＜10 CFU/克
霉菌和酵母菌总数	≤10^2 CFU/克（最大可接受200 CFU/克）	＜10 CFU/克
大肠埃希菌	不得检出	未检出

以下空白

结论：本品按《醋北柴胡配方颗粒国家药品标准（YBZ-PFKL-2021030）》检验，结果符合规定。

批准人：[signature] 2025.12.14　　复核人：[signature] 2025.12.14　　编制人：[signature] 2025.12.14

四川新绿色药业科技发展有限公司 质检专用章

声明：1、本报告检验结果仅对送检样品负责；
2、对本报告若有异议，应于收到报告之日起七个工作日内向本中心提出，逾期将自动视为承认本检测报告。

检测中心地址：四川省彭州市致和镇东河东路279号　　电话号码： 028-83888888

成品检验报告书

文件编号：XLS-QC-MP-0007-OR03(00)

报告书编号： C90F003—25017

品　名	茯苓配方颗粒	规　格	100g/袋（每1g配方颗粒相当于饮片12.5g）
批　号	25110240	产　地	云南
代表量	11427 袋	收样日期	2025年11月24日
来　源	包装工序	报告日期	2025年11月30日
检验依据	《茯苓配方颗粒（SCYPBZ（PFKL）-2021241）》		

检验项目	检验标准	检验结果
性　状	本品应为浅灰色至灰黄色的颗粒；气微，味淡。	符合规定
薄层鉴别	供试品色谱中，在与茯苓酸A对照品色谱相应的位置上，应显相同颜色的荧光斑点。	符合规定
特征图谱	应符合规定	符合规定
检　查		
粒　度	不能通过一号筛与能通过五号筛的总和应≤15%	4.7%
水　分	≤8.0%	3.1%
溶化性	应全部溶化或轻微浑浊，不得有焦屑或异物	符合规定
装　量	平均装量应≥100g，每袋装量应≥97g	符合规定
浸出物	乙醇热浸出物≥15.0%	27.3%
含量测定	茯苓酸B应为0.10mg/g～0.70mg/g	0.28mg/g
	茯苓酸A应为0.09mg/g～0.60mg/g	0.32mg/g
微生物限度检查		
需氧菌总数	≤10^3 CFU/克（最大可接受2000 CFU/克）	<10 CFU/克
霉菌和酵母菌总数	≤10^2 CFU/克（最大可接受200 CFU/克）	<10 CFU/克
大肠埃希菌	不得检出	未检出

以下空白

结论：本品按《茯苓配方颗粒（SCYPBZ（PFKL）-2021241）》检验，结果符合规定。

批准人：[signature] 2025.11.30　　复核人：[signature] 2025.11.30　　编制人：[signature] 2025.11.30

四川新绿色药业科技发展有限公司 质检专用章

声明：1、本报告检验结果仅对送检样品负责；
2、对本报告若有异议，应于收到报告之日起七个工作日内向本中心提出，逾期将自动视为承认本检测报告。

检测中心地址：四川省彭州市致和镇东河东路279号　　电话号码：028-83888888
