# Supplementary material for: Modified sini powder for the management of postoperative depression in non-small cell lung cancer patients: a multicenter, randomized, double-blind, placebo-controlled trial protocol
Source: Front Pharmacol. 2026 Jun 19;17:1805554. doi: 10.3389/fphar.2026.1805554 (PMC13327883; doi:10.3389/fphar.2026.1805554)
Supplement: Supplementary file 1 [file DataSheet1.zip › Supplementary materials/Supplementary material 4.pdf]

Quantitative Analysis of Chemical Components in Modified Sini Powder (MSNP)

1. Materials and Methods

1.1. Reagents and Materials

High-performance liquid chromatography (HPLC)-grade methanol (Thermo Fisher Scientific, USA; Cat. No. A452-4) and acetonitrile (Thermo Fisher Scientific, USA; Cat. No. A998-4), as well as liquid chromatography-mass spectrometry (LC-MS)-grade formic acid (Thermo Fisher Scientific, USA; Cat. No. A117-50), were employed in this study. Deionized water was prepared using a Milli-Q purification system (Millipore, USA). A working solution of mixed internal standards was prepared as required.

1.2. Instruments and Equipment

The following instruments were used: an ultrasonic cleaner (Model F-060SD, Fuyang Technology, China), a vortex mixer (Model TYXH-I, Hanno Instruments, China), a refrigerated benchtop centrifuge (Model 5430R, Eppendorf, Germany), an ultra-performance liquid chromatography system (ACQUITY UPLC I-Class, Waters, USA), and a high-resolution hybrid quadrupole-Orbitrap mass spectrometer (Q Exactive HF, Thermo Fisher Scientific, USA). Chromatographic separation was achieved using an ACQUITY UPLC HSS T3 column (100 mm × 2.1 mm, 1.8 μm; Waters, USA).

1.3. Sample Preparation

Approximately 100 mg of finely ground MSNP sample was accurately weighed into a 1.5 mL microcentrifuge tube. Subsequently, 1.0 mL of ice-cold extraction solvent (70% aqueous methanol containing a mixed internal standard at 2 μg/mL) was added. The

mixture was vortexed vigorously for 1 minute. Following the addition of a stainless-steel grinding bead, the sample was equilibrated at -40°C for 2 minutes and then homogenized using a tissue lyser at 60 Hz for 2 minutes. Ultrasound-assisted extraction (UAE) was then performed in an ice-water bath for 60 minutes. The extract was centrifuged at 12,000 rpm and 4°C for 10 minutes. Finally, a 150 μL aliquot of the supernatant was transferred to an LC-MS vial equipped with an insert for subsequent analysis.

1.4. LC-MS/MS Analysis

LC-MS/MS analysis was conducted by Shanghai Oebiotech Co., Ltd. (Shanghai, China).

Chromatographic Conditions: Separation was performed on an ACQUITY UPLC HSS T3 column (100 mm × 2.1 mm, 1.8 μm) maintained at 45°C. The mobile phase consisted of (A) 0.1% (v/v) formic acid in water and (B) acetonitrile. The flow rate was set at 0.35 mL/min with an injection volume of 2 μL. The following gradient elution program was applied: 0-3 min, 0% B; 3-18.5 min, 0-20% B; 18.5-20 min, 20-35% B; 20-26 min, 35-40% B; 26-35 min, 40-95% B; 35-38 min, 95% B; 38-38.1 min, 95-0% B; 38.1-40 min, 0% B. The autosampler temperature was maintained at 4°C.

Mass Spectrometric Conditions: Detection was carried out using a Q Exactive HF mass spectrometer equipped with a heated electrospray ionization (HESI) source, operating in both positive and negative ion modes. The instrument was operated in data-dependent acquisition (DDA) mode. Full MS scans were acquired over a mass range of *m/z* 100-1500 at a resolution of 60,000. The top 10 most intense ions were selected

for higher-energy collisional dissociation (HCD) MS/MS scans (dd-MS2) at a resolution of 15,000, using stepped normalized collision energies (NCE) of 10, 20, and 40 eV. Key source parameters were as follows: spray voltage, 3800 V (positive) and -3200 V (negative); sheath gas flow rate, 35 arbitrary units (arb); auxiliary gas flow rate, 8 arb; capillary temperature, 320°C; auxiliary gas heater temperature, 350°C; S-lens RF level, 50.

1.5. Data Processing and Compound Identification

Raw LC-MS data were processed using XCMS software (version 4.5.1) for baseline filtering, peak detection, integration, retention time alignment, and normalization. Compound identification was primarily performed by matching the experimental data—including accurate mass (with a mass error tolerance < 5 ppm), isotopic distribution patterns, and MS/MS fragmentation spectra—against the proprietary LuMet-TCM (Luming Metabolite-Traditional Chinese Medicine) database (Oebiotech, China) and the public Herb database. The LuMet-TCM database comprises over 5,000 authenticated reference standards, with curated metadata encompassing retention time, accurate mass, MS/MS spectra, chemical classification, and botanical origin.

Metabolites with a total identification score exceeding 40 were retained for further analysis. Data acquired in positive and negative ionization modes were merged, deduplicated, and compiled into a final qualitative and quantitative data matrix. For relative quantification, the total ion peak area of all identified metabolites in this matrix was normalized to 100%.

To ensure rigorous identification, a tripartite criterion was applied: (1) retention time deviation from the database standard within ± 0.3 min; (2) precursor ion mass error within 5 ppm; and (3) high spectral similarity between experimental and reference MS/MS patterns. For compounds lacking MS/MS spectra, identification was based on criteria (1) and (2). Extracted ion chromatograms (EIC) and annotated MS/MS mirror plots were generated for each confidently identified compound. A pie chart was constructed to visualize the distribution of identified MSNP constituents across major chemical classes.

2. Results

Comprehensive analysis involving precise mass-to-charge ratio (m/z) matching, secondary fragment ion evaluation, and isotopic pattern comparison against the LuMet-TCM reference database resulted in the identification of 1,671 chemical components within the MSNP formulation (see table below).

The chemical compositions of Modified Sini Powder (MSNP).

No.	Identification	Formula	Adducts	Mass Error (ppm)	theoretical m/z	m/z	Retention time (min)	Ion mode
1	p-Hydroxybenzaldehyde	C7H6O2	M+H-H2O	0.095207	105.03408	105.03409	11.41	POS
2	Tropone	C7H6O	M+H	5.885148	107.04914	107.04977	9.17	POS
3	Pyrogallol	C6H6O3	M-H	-8.718297	125.02442	125.02333	3.342	NEG
4	(R)-pyrrolidine-2-carboxylic acid	C5H9NO2	M+H	4.82465	116.0706	116.07116	0.806	POS

5	DL-Norvaline	C5H11NO2	M+H	3.980142	118.08625	118.08672	0.78	POS
6	D-Allothreonine	C4H9NO3	M+H	2.748499	120.06552	120.06585	0.732	POS
7	L-Threonic acid	C4H8O5	M-H	-7.924171	135.0299	135.02883	0.766	NEG
8	Niacin	C6H5NO2	M+H	3.708502	124.0393	124.03976	1.534	POS
9	Pipecolinic acid	C6H11NO2	M+H	6.687871	130.08625	130.08712	1.23	POS
10	Protocatechuic acid	C7H6O4	M-H	-5.685556	153.01933	153.01846	6.984	NEG
11	Arabinitol	C5H12O5	M+H-H2O	-3.035558	135.06577	135.06536	0.78	POS
12	3-Methyladipic acid	C7H12O4	M-H	-5.092217	159.06628	159.06547	11.601	NEG
13	4-Methoxybenzaldehyde	C8H8O2	M+H	0.583687	137.05971	137.05979	20.071	POS
14	Trigonelline	C7H7NO2	M+H	1.086524	138.05495	138.0551	0.834	POS
15	Methyl protocatechuate	C8H8O4	M-H	-6.525579	167.03498	167.03389	14.257	NEG
16	4-Aminocinnamic Acid	C9H9NO2	M+H-H2O	-1.985477	146.06063	146.06034	9.499	POS
17	Glucose	C6H12O6	M-H	-4.076934	179.05611	179.05538	0.762	NEG
18	Dihydrocaffeic acid	C9H10O4	M-H	-5.026218	181.05063	181.04972	10.877	NEG
19	3-O-Methylgallic acid	C8H8O5	M-H	-5.736768	183.0299	183.02885	8.969	NEG
20	Synephrine	C9H13NO2	M+H-H2O	-2.331904	150.09193	150.09158	1.453	POS
21	2,3,4-Trihydroxybenzoic acid	C7H6O5	M+H-H2O	-1.633786	153.01882	153.01857	7.646	POS
22	Vanillin	C8H8O3	M+H	1.045378	153.05462	153.05478	13.701	POS
23	Noreugenin	C10H8O4	M-H	-3.873636	191.03498	191.03424	19.98	NEG

24	(E)-Ferulic acid	C10H10O4	M-H	-3.004393	193.05063	193.05005	15.986	NEG
25	Manosamine	C6H13NO5	M+H-H2O	-2.28287	162.07667	162.0763	0.755	POS
26	Levoglucosan	C6H10O5	M+H	0.858579	163.0601	163.06024	0.941	POS
27	Fraxetin	C10H8O5	M-H	-2.41511	207.0299	207.0294	13.832	NEG
28	Polygonatine A	C9H11NO2	M+H	1.565452	166.08625	166.08651	9.808	POS
29	3-Hydroxydodecanoic acid	C12H24O3	M-H	-1.859036	215.16527	215.16487	29.147	NEG
30	D-Pantothenic acid (hemicalcium salt)	C9H17NO5	M-H	-1.192095	218.1034	218.10314	7.827	NEG
31	Gallic acid	C7H6O5	M+H	1.052454	171.0288	171.02898	3.679	POS
32	(Z)-Aconitic acid	C6H6O6	M+H	0	175.02371	175.02371	1.574	POS
33	Demethylsuberosin	C14H14O3	M-H	-1.658758	229.08702	229.08664	27.268	NEG
34	Armillarisin A	C12H10O5	M-H	-0.12873	233.04555	233.04552	16.256	NEG
35	5,7-Dihydroxychromone	C9H6O4	M+H	2.234214	179.03388	179.03428	16.63	POS
36	Coniferaldehyde	C10H10O3	M+H	0.83766	179.07027	179.07042	17.966	POS
37	Fusaric acid	C10H13NO2	M+H	1.499151	180.1019	180.10217	13.867	POS
38	4',5-Dihydroxyflavone	C15H10O4	M-H	-1.501676	253.05063	253.05025	25.896	NEG
39	7,4'-Dihydroxyflavone	C15H10O4	M-H	0.039518	253.05063	253.05064	20.566	NEG
40	Aceglutamide	C7H12N2O4	M+H	0.264429	189.08698	189.08703	1.453	POS
41	Vasicine	C11H12N2O	M+H	0.951866	189.10224	189.10242	7.91	POS

42	Laminine	C9H21N2O2+	M+	-0.158596	189.15975	189.15972	0.693	POS
43	N-acetyldopamine	C10H13NO3	M+H	1.27488	196.09682	196.09707	9.397	POS
44	Allopurinol riboside	C10H12N4O5	M-H	1.160729	267.07349	267.0738	5.529	NEG
45	Apigenin	C15H10O5	M-H	2.081432	269.04555	269.04611	21.907	NEG
46	Sulfuretin	C15H10O5	M-H	-0.297347	269.04555	269.04547	20.736	NEG
47	Dihydrokaempferol	C15H12O6	M-H2O-H	2.564627	269.045	269.04569	19.498	NEG
48	Naringenin	C15H12O5	M-H	0.516489	271.0612	271.06134	21.785	NEG
49	Butin	C15H12O5	M-H	-1.401897	271.0612	271.06082	19.351	NEG
50	Vestitol	C16H16O4	M-H	-0.295097	271.09758	271.0975	23.614	NEG
51	2-Hydroxypalmitic acid	C16H32O3	M-H	1.695991	271.22787	271.22833	33.88	NEG
52	3,4,8,9,10-Pentahydroxy Urolithin	C13H8O7	M-H	-0.872665	275.01973	275.01949	14.53	NEG
53	N-Acetyl-D-glucosamine	C8H15NO6	M+H-H2O	-1.175968	204.08724	204.087	0.796	POS
54	Crotonoside	C10H13N5O5	M-H	-0.638107	282.08439	282.08421	5.571	NEG
55	Eugenin	C11H10O4	M+H	1.400525	207.06518	207.06547	23.704	POS
56	Licochalcone B	C16H14O5	M-H	-0.526174	285.07685	285.0767	20.736	NEG
57	Eriodictyol	C15H12O6	M-H	0.557382	287.05611	287.05627	20.818	NEG
58	Sinapaldehyde	C11H12O4	M+H	0.621769	209.08083	209.08096	18.577	POS
59	Sarracenin	C11H14O5	M+H-H2O	-0.478283	209.08142	209.08132	19.938	POS
60	Harmine	C13H12N2O	M+H	2.205514	213.10224	213.10271	16.819	POS
61	Tectorigenin	C16H12O6	M-H	-0.367824	299.05611	299.056	21.973	NEG

62	Hydroxygenkwanin	C16H12O6	M-H	-0.601894	299.05611	299.05593	23.508	NEG
63	Gallocatechin	C15H14O7	M-H	1.442308	305.06668	305.06712	7.786	NEG
64	Flazin	C17H12N2O4	M-H	-0.358222	307.07243	307.07232	21.155	NEG
65	Alismoxide	C15H26O2	M+H-H2O	-2.124864	221.19058	221.19011	21.753	POS
66	Vanillic acid 4-beta-D-glucopyranoside	C14H18O9	M-H	-1.610512	329.08781	329.08728	8.356	NEG
67	Salicin	C13H18O7	M+FA-H	0	331.10345	331.10345	9.232	NEG
68	(-)-Sparteine	C15H26N2	M+H	0.510167	235.21687	235.21699	8.427	POS
69	Dimethylfraxetin	C12H12O5	M+H	2.78392	237.07575	237.07641	21.044	POS
70	Rhombifoline	C15H20N2O	M+H	0.7342	245.16484	245.16502	6.525	POS
71	Isovalerylcarnitine	C12H23NO4	M+H	1.624893	246.16998	246.17038	10.954	POS
72	Glycyrrhisoflavone	C20H18O6	M-H	-1.61426	353.10306	353.10249	28.373	NEG
73	Licoflavonol	C20H18O6	M-H	0.679688	353.10306	353.1033	29.919	NEG
74	2,3-Dehydrokievitone	C20H18O6	M-H	1.132814	353.10306	353.10346	27.506	NEG
75	Albanin A	C20H18O6	M-H	-1.217775	353.10306	353.10263	26.939	NEG
76	Sanggenone H	C20H18O6	M-H	0.283203	353.10306	353.10316	28.79	NEG
77	Isolicoflavonol	C20H18O6	M-H	1.812502	353.10306	353.1037	29.168	NEG
78	Isopimpinellin	C13H10O5	M+H	2.954747	247.0601	247.06083	23.558	POS
79	(±)-Abscisic acid	C15H20O4	M+H-H2O	0.687887	247.13346	247.13363	20.853	POS
80	Glyasperin C	C21H24O5	M-H	-1.435992	355.1551	355.15459	27.993	NEG
81	Matrine	C15H24N2O	M+H	0.521677	249.19614	249.19627	7.277	POS

82	Cordycepin	C10H13N5O3	M+H	-0.356988	252.10912	252.10903	5.825	POS
83	Neoglycyrol	C21H18O6	M-H	-0.684738	365.10306	365.10281	29.085	NEG
84	Daidzein	C15H10O4	M+H	1.45061	255.06518	255.06555	20.711	POS
85	Nicotinic acid riboside	C11H13NO6	M+H	1.601052	256.08156	256.08197	1.132	POS
86	Isoicaritin	C21H20O6	M-H	-0.32687	367.11871	367.11859	25.373	NEG
87	Icaritin	C21H20O6	M-H	-0.245152	367.11871	367.11862	31.609	NEG
88	Glycycoumarin	C21H20O6	M-H	-0.027239	367.11871	367.1187	27.686	NEG
89	Liquiritigenin	C15H12O4	M+H	1.12805	257.08083	257.08112	20.402	POS
90	Isoliquiritigenin	C15H12O4	M+H	-0.700169	257.08083	257.08065	23.122	POS
91	(±)-Liquiritigenin	C15H12O4	M+H	1.478134	257.08083	257.08121	20.782	POS
92	Skimmin	C15H16O8	M+FA-H	-0.081283	369.08272	369.08269	10.565	NEG
93	Fraxin	C16H18O10	M-H	-0.054188	369.08272	369.0827	12.893	NEG
94	Meranzin	C15H16O4	M+H	-7.659545	261.11213	261.11013	23.495	POS
95	Isomerazin	C15H16O4	M+H	0.995741	261.11213	261.11239	24.337	POS
96	Micromarin F	C15H16O4	M+H	0.84255	261.11213	261.11235	22.437	POS
97	Feruloylputrescine	C14H20N2O3	M+H	1.395412	265.15467	265.15504	11.056	POS
98	Licoricone	C22H22O6	M-H	0.498512	381.13436	381.13455	28.98	NEG
99	S-Marmesin	C14H14O4	M+Na	5.723238	269.07843	269.07997	20.479	POS
100	Dalbergin	C16H12O4	M+H	2.19265	269.08083	269.08142	23.586	POS
101	3,7,4'-Trihydroxyflavone	C15H10O5	M+H	0.959197	271.0601	271.06036	20.981	POS
102	Medicarpin	C16H14O4	M+H	0.553309	271.09648	271.09663	25.381	POS

103	Gentiopicroside	C16H20O9	M+FA-H	-0.373963	401.10893	401.10878	13.166	NEG
104	Dimethoxydaidzein	C17H14O4	M+H	-0.035324	283.09648	283.09647	28.604	POS
105	3'-Methoxydaidzein	C16H12O5	M+H	0.841881	285.07575	285.07599	20.937	POS
106	Calycosin	C16H12O5	M+H	0.701568	285.07575	285.07595	21.128	POS
107	Texasin	C16H12O5	M+H	0	285.07575	285.07575	22.022	POS
108	Acacetin	C16H12O5	M+H	0.175392	285.07575	285.0758	26.27	POS
109	Genkwanin	C16H12O5	M+H	0.035078	285.07575	285.07576	26.629	POS
110	Wogonin	C16H12O5	M+H	0.140314	285.07575	285.07579	25.764	POS
111	Negletein	C16H12O5	M+H	0.596333	285.07575	285.07592	24.563	POS
112	α,α-Trehalose 6-phosphate	C12H23O14P	M-H	-1.424923	421.07527	421.07467	0.728	NEG
113	Brevifolincarboxylic acid	C13H8O8	M+H	0	293.02919	293.02919	12.038	POS
114	Guaijaverin	C20H18O11	M-H	0.023091	433.07764	433.07765	18.471	NEG
115	Phlorizin	C21H24O10	M-H	0.459633	435.12967	435.12987	20.085	NEG
116	Hamaudol	C15H16O5	M+Na	8.291847	299.08899	299.09147	24.082	POS
117	Astragalin	C21H20O11	M-H	1.543302	447.09329	447.09398	19.314	NEG
118	Pratensein	C16H12O6	M+H	2.192176	301.07066	301.07132	22.688	POS
119	Diosmetin	C16H12O6	M+H	1.195733	301.07066	301.07102	22.332	POS
120	Eriodictyol-7-O-glucoside	C21H22O11	M-H	0.133598	449.10894	449.109	17.021	NEG
121	Hesperetin	C16H14O6	M+H	1.418738	303.08631	303.08674	22.332	POS
122	Phenylethyl-beta-D-galactoside	C14H20O6	M+Na	0.097683	307.11521	307.11524	14.697	POS
123	Tectoridin	C22H22O11	M-H	3.144593	461.10894	461.11039	18.535	NEG

124	Hesperetin 7-O-glucoside	C22H24O11	M-H	0.345479	463.12459	463.12475	20.189	NEG
125	Methylarbutin	C13H18O7	M+Na	-1.488218	309.09447	309.09401	10.353	POS
126	Sinapine	C16H24NO5+	M+	-0.515855	310.1649	310.16474	12.99	POS
127	bakkenolide A	C15H22O2	2M-H	1.604907	467.31668	467.31743	31.882	NEG
128	23-Hydroxybetulinic acid	C30H48O4	M-H	0.530394	471.34798	471.34823	30.572	NEG
129	Polyporenic acid C	C31H46O4	M-H	0.893354	481.33233	481.33276	32.492	NEG
130	Isorhamnetin	C16H12O7	M+H	0.78848	317.06558	317.06583	22.437	POS
131	Poricoic acid B	C30H44O5	M-H	0.248287	483.3116	483.31172	31.223	NEG
132	Ilexgenin A	C30H46O6	M-H2O-H	1.34489	483.31105	483.3117	30.11	NEG
133	Glycitin	C22H22O10	M+FA-H	1.547485	491.1195	491.12026	16.089	NEG
134	Licoflavone A	C20H18O4	M+H	0.49516	323.12779	323.12795	26.354	POS
135	Laricitrin 3-O-glucoside	C22H22O13	M-H	1.196515	493.09876	493.09935	17.503	NEG
136	A-D-Glucopyranoside	C21H36O10	M+FA-H	2.797889	493.22905	493.23043	20.778	NEG
137	Apiopaeonoside	C20H28O12	M+FA-H	1.662852	505.15628	505.15712	14.001	NEG
138	Paeonolide	C20H28O12	M+FA-H	1.306526	505.15628	505.15694	13.396	NEG
139	Sinomenine	C19H23NO4	M+H	2.059545	330.16998	330.17066	11.098	POS
140	Lactiflorin	C23H26O10	M+FA-H	0.709848	507.1508	507.15116	20.251	NEG
141	Rosiridin	C16H28O7	M+H	-0.540231	333.19078	333.1906	17.779	POS
142	3-O-Acetyl-16alpha-hydroxydehydrotrametenolic acid	C32H48O5	M-H	-1.349388	511.3429	511.34221	33.584	NEG

143	3-O-Acetyl-16alpha-hydroxytrametenolic acid	C32H50O5	M-H	1.090856	513.35855	513.35911	33.855	NEG
144	Deoxyandrographolide	C20H30O4	M+H	-6.980455	335.22169	335.21935	24.688	POS
145	1,3-Dicaffeoylquinic acid	C25H24O12	M-H	2.581925	515.1195	515.12083	19.02	NEG
146	1,4-Dicaffeoylquinic acid	C25H24O12	M-H	-0.310608	515.1195	515.11934	18.6	NEG
147	18beta-Glycyrrhetinic acid	C30H46O4	M+FA-H	0.388095	515.33781	515.33801	32.571	NEG
148	Alpinumisoflavone	C20H16O5	M+H	0.889925	337.10705	337.10735	30.934	POS
149	Maslinic acid	C30H48O4	M+FA-H	0.173962	517.35346	517.35355	31.65	NEG
150	Licoflavone C	C20H18O5	M+H	0.471806	339.1227	339.12286	28.095	POS
151	9,10-Dihydroxystearic acid	C18H36O4	M+Na	1.709651	339.25058	339.25116	30.502	POS
152	Esculin	C15H16O9	M+H	-1.700447	341.08671	341.08613	10.373	POS
153	8-Prenylnaringenin	C20H20O5	M+H	-0.908722	341.13835	341.13804	27.99	POS
154	Magnoflorine	C20H24NO4+	M+	1.198235	342.16998	342.17039	14.263	POS
155	Dehydropachymic acid	C33H50O5	M-H	-0.095173	525.35855	525.3585	33.616	NEG
156	Turanose	C12H22O11	M+H	0.174864	343.12349	343.12355	0.827	POS
157	Viscidulin III	C17H14O8	M+H	-1.296545	347.07614	347.07569	20.208	POS
158	Paeoniflorin sulfite	C23H28O13S	M-H	0.405069	543.11779	543.11801	10.708	NEG
159	Palmatine	C21H22NO4+	M+	0.08519	352.15433	352.15436	20.709	POS
160	Poricoic acid A	C31H46O5	M+FA-H	-0.883437	543.33272	543.33224	31.779	NEG

161	Protopine	C20H19NO5	M+H	0.8189	354.1336	354.13389	18.09	POS
162	Tetramethylkaempferol	C19H18O6	M+Na	-0.657355	365.09956	365.09932	27.444	POS
163	6-Demethoxytangeretin	C19H18O6	M+Na	-0.273898	365.09956	365.09946	24.772	POS
164	Scutellarein tetramethyl ether	C19H18O6	M+Na	-1.28732	365.09956	365.09909	26.693	POS
165	Procyanidin B3	C30H26O12	M-H	0.502482	577.13515	577.13544	10.926	NEG
166	Rhoifolin	C27H30O14	M-H	0.675727	577.15628	577.15667	19.707	NEG
167	Isorhoifolin	C27H30O14	M-H	0.693053	577.15628	577.15668	19.167	NEG
168	Benzoylpaeoniflorin	C30H32O12	M-H	-0.034295	583.1821	583.18208	21.7	NEG
169	Wushanicaritin	C21H22O7	M+H-H2O	-0.893985	369.13385	369.13352	28.306	POS
170	Glicoricone	C21H20O6	M+H	0.216724	369.13326	369.13334	24.919	POS
171	Poncirin	C28H34O14	M-H	2.309556	593.18758	593.18895	20.946	NEG
172	Tangeretin	C20H20O7	M+H	-1.23282	373.12818	373.12772	28.286	POS
173	Skullcapflavone II	C19H18O8	M+H	0.533181	375.10744	375.10764	26.8	POS
174	Rutin	C27H30O16	M-H	1.592393	609.14611	609.14708	17.212	NEG
175	Apiin	C26H28O14	M+FA-H	1.214815	609.1461	609.14684	19.56	NEG
176	Neohesperidin	C28H34O15	M-H	-1.001342	609.18249	609.18188	20.128	NEG
177	Neochlorogenic acid	C16H18O9	M+Na	-0.026519	377.0843	377.08429	9.232	POS
178	Cryptochlorogenic acid	C16H18O9	M+Na	0	377.0843	377.0843	11.911	POS
179	Trans-ferulic acid-4-beta-glucoside	C16H20O9	M+Na	-1.266157	379.09995	379.09947	11.159	POS
180	Matairesinol	C20H22O6	M+Na	-0.026238	381.13086	381.13085	22.106	POS

181	Galloylpaeoniflorin	C30H32O15	M-H	0.839715	631.16684	631.16737	17.819	NEG
182	6'-O-galloylalbiflorin	C30H32O15	M-H	-0.522841	631.16684	631.16651	20.387	NEG
183	1,3,6-Tri-O-galloyl-beta-D-glucose	C27H24O18	M-H	1.590328	635.08899	635.09	14.28	NEG
184	Genistein 7,4'-di-O-beta-D-glucopyranoside	C27H30O15	M+FA-H	2.049576	639.15667	639.15798	13.543	NEG
185	Kobusin	C21H22O6	M+NH4	-1.133508	388.17546	388.17502	28.183	POS
186	3'-Demethylnobiletin	C20H20O8	M+H	-0.950856	389.12309	389.12272	23.64	POS
187	Arctigenin	C21H24O6	M+NH4	2.101534	390.19111	390.19193	23.787	POS
188	Cyclocurcumin	C21H20O6	M+Na	-0.690334	391.11521	391.11494	23.059	POS
189	Nortrachelogenin	C20H22O7	M+NH4	2.447916	392.17038	392.17134	20.981	POS
190	Glabrol	C25H28O4	M+H	0.025432	393.20604	393.20605	30.764	POS
191	Prosaikogenin G	C36H58O8	M+FA-H	2.562513	663.41137	663.41307	30.302	NEG
192	Prosaikogenin D	C36H58O8	M+FA-H	3.828695	663.41137	663.41391	28.181	NEG
193	Isomaltotetraose	C24H42O21	M-H	2.660796	665.21458	665.21635	1.296	NEG
194	Syringaresinol	C22H26O8	M+H-H2O	-2.467843	401.16007	401.15908	21.326	POS
195	Nobiletin	C21H22O8	M+H	0.396886	403.13874	403.1389	26.629	POS
196	TMC-58B	C25H26N2O3	M+H	0.49603	403.20162	403.20182	25.785	POS
197	Schisanhenol	C23H30O6	M+H	0.049602	403.21151	403.21153	31.491	POS
198	Glycyrrhetic acid 3-O-beta-D-glucuronide	C36H54O10	M+FA-H	2.299782	691.3699	691.37149	29.251	NEG

199	Roseoside	C19H30O8	M+NH4	-0.470032	404.22789	404.2277	13.721	POS
200	5-O-Demethylnobiletin	C20H20O8	M+Na	-2.018949	411.10504	411.10421	29.297	POS
201	Puerarin	C21H20O9	M+H	-0.191792	417.11801	417.11793	13.553	POS
202	Daidzin	C21H20O9	M+H	0.407559	417.11801	417.11818	15.318	POS
203	Diphyllin	C21H16O7	M+K	-1.240894	419.05276	419.05224	25.298	POS
204	Natsudaidain	C21H22O9	M+H	-1.741688	419.13366	419.13293	28.35	POS
205	Neoisoliquiritin	C21H22O9	M+H	1.741688	419.13366	419.13439	20.531	POS
206	Liquiritin	C21H22O9	M+H	0.262446	419.13366	419.13377	17.067	POS
207	Neoliquiritin	C21H22O9	M+H	1.717829	419.13366	419.13438	16.589	POS
208	Schisandrin B	C23H28O6	M+Na	-1.157906	423.17781	423.17732	33.284	POS
209	Broussoflavonol F	C25H26O6	M+H	0.921593	423.18021	423.1806	31.686	POS
210	Quercetagetin 3,5,6,7,3',4'-hexamethyl ether	C21H22O8	M+Na	0.564546	425.12069	425.12093	25.997	POS
211	Syringaresinol-di-O-glucoside	C34H46O18	M+FA-H	2.222881	787.26661	787.26836	16.53	NEG
212	Ononin	C22H22O9	M+H	0.440699	431.13366	431.13385	20.479	POS
213	Saikosaponin E	C42H68O12	M+FA-H	1.976604	809.46928	809.47088	28.373	NEG
214	Genistin	C21H20O10	M+H	0.184709	433.11292	433.113	17.904	POS
215	Vitexin	C21H20O10	M+H	0.34633	433.11292	433.11307	17.046	POS
216	Saikosaponin B2	C42H68O13	M+Cl	2.93095	815.43539	815.43778	26.648	NEG
217	3,5,6,7,8,3',4'-Heptemthoxyflavone	C22H24O9	M+H	0.392474	433.14931	433.14948	27.855	POS

218	5-MethoxyPinocembroside	C22H24O9	M+H	0.507908	433.14931	433.14953	19.903	POS
219	Schisandrol B	C23H28O7	M+NH4	-1.128467	434.21733	434.21684	28.855	POS
220	glycyrrhizinate	C42H62O16	M-H	3.372306	821.39651	821.39928	24.599	NEG
221	Saikosaponin D	C42H68O13	M+FA-H	0.823779	825.46419	825.46487	29.168	NEG
222	Morusinol	C25H26O7	M+H	-2.231456	439.17513	439.17415	28.981	POS
223	Schisandrin A	C24H32O6	M+Na	0.432596	439.20911	439.2093	32.746	POS
224	Zingibroside R1	C42H66O14	M+FA-H	3.561884	839.44346	839.44645	28.728	NEG
225	Tibesaikosaponin V	C42H68O15	M+FA-H	4.396737	857.45402	857.45779	21.595	NEG
226	Saikosaponin B4	C43H72O14	M+FA-H	1.644333	857.49041	857.49182	24.805	NEG
227	6''-O-Acetylsaikosaponin A	C44H70O14	M+FA-H	4.518863	867.47476	867.47868	28.54	NEG
228	Soysaponin II	C47H76O17	M-H	-0.756993	911.50097	911.50028	27.359	NEG
229	Catechin gallate	C22H18O10	M+NH4	1.803862	460.12382	460.12465	17.633	POS
230	Soyasaponin Bb	C48H78O18	M-H	2.665926	941.51154	941.51405	26.419	NEG
231	Swertisin	C22H22O10	M+Na	0.532923	469.11051	469.11076	17.883	POS
232	Saikosaponin H	C48H78O17	M+Cl	1.996894	961.4933	961.49522	23.845	NEG
233	Saikosaponin F	C48H80O17	M+FA-H	1.828383	973.53775	973.53953	23.401	NEG
234	Orcinol gentiobioside	C19H28O12	M+Na	1.294712	471.14729	471.1479	8.964	POS
235	Saikosaponin S	C48H78O18	M+FA-H	0.911377	987.51701	987.51791	21.7	NEG
236	Aurantiamide acetic acid	C27H28N2O4	M+K	1.13832	483.16807	483.16862	29.131	POS
237	Jujuboside B	C52H84O21	M+FA-H	-0.541509	1089.54871	1089.54812	24.32	NEG
238	Paeoniflorin	C23H28O11	M+NH4	1.184271	498.19698	498.19757	14.853	POS

239	Hesperidin	C28H34O15	2M-H	3.034348	1219.37226	1219.37596	19.855	NEG
240	Albiflorin	C23H28O11	M+Na	-2.166342	503.15238	503.15129	19.943	POS
241	Oxypaeoniflorin	C23H28O12	M+Na	1.367627	519.14729	519.148	11.637	POS
242	Angeloylgomisin O	C28H34O8	M+Na	1.285459	521.21459	521.21526	33.821	POS
243	1-Kestose	C18H32O16	M+Na	2.409144	527.15825	527.15952	1.285	POS
244	Tigloylgomisin P	C28H34O9	M+Na	-1.042424	537.2095	537.20894	31.277	POS
245	Licraside	C26H30O13	M+H	2.413023	551.17592	551.17725	20.312	POS
246	Nortracheloside	C26H32O12	M+Na	3.040174	559.17859	559.18029	17.841	POS
247	Schisantherin A	C30H32O9	M+Na	1.806172	559.19385	559.19486	31.151	POS
248	Liquiritigenin-7-o-apiosyl(1-2)-glucoside	C26H30O13	M+Na	0.157025	573.15786	573.15795	16.755	POS
249	Liquiritin apioside	C26H30O13	M+Na	0.191919	573.15786	573.15797	17.087	POS
250	Meloside A	C27H30O15	M+H	2.033047	595.16575	595.16696	16.589	POS
251	Vicenin 2	C27H30O15	M+H	1.579392	595.16575	595.16669	14.281	POS
252	Lyoniresinol 9'-O-glucoside	C28H38O13	M+NH4	0.566416	600.26506	600.2654	16.087	POS
253	Narirutin	C27H32O14	M+Na	0.812377	603.16842	603.16891	18.678	POS
254	Benzoylalbiflorin	C30H32O12	M+Na	3.129228	607.17859	607.18049	22.21	POS
255	Spinosin	C28H32O15	M+H	2.10118	609.1814	609.18268	17.275	POS
256	Neoeriocitrin	C27H32O15	M+NH4	-0.244217	614.20794	614.20779	17.507	POS
257	Nicotiflorin	C27H30O15	M+Na	-0.162036	617.14769	617.14759	18.8	POS
258	Narcissin	C28H32O16	M+H	1.391607	625.17631	625.17718	19.266	POS

259	4-O-galloylalbiflorin	C30H32O15	M+H	-0.552764	633.1814	633.18105	19.551	POS
260	(-)-Syringaresnol-4-O-β-D-apiofuranosyl-(1→2)-β-D-glucopyranoside	C33H44O17	M+Na	3.141801	735.24707	735.24938	19.634	POS
261	Pseudoginsenoside F11	C42H72O14	M+H-H2O	1.340158	783.48951	783.49056	22.063	POS
262	6-Feruloylspinosin	C38H40O18	M+H	4.941235	785.22874	785.23262	20.061	POS
263	Bupleuroside XIII	C42H70O14	M+H	-0.237653	799.48383	799.48364	21.36	POS
264	Saikosaponin B1	C42H68O13	M+Na	0.535189	803.45521	803.45564	27.82	POS
265	Saikosaponin A	C42H68O13	M+Na	0.49785	803.45521	803.45561	26.311	POS
266	1,2,3,6-Tetragalloylglucose	C34H28O22	M+NH4	-0.756691	806.14105	806.14044	16.917	POS
267	Licorice-saponin H2	C42H62O16	M+H	3.631236	823.41106	823.41405	26.101	POS
268	Licorice saponin G2	C42H62O17	M+H	2.608988	839.40598	839.40817	23.306	POS
269	Saikosaponin C	C48H78O17	M+H-H2O	-0.087958	909.5212	909.52112	23.079	POS
270	Buddlejasaponin IVb	C48H78O18	M+H	2.119708	943.52609	943.52809	25.512	POS
271	Nepasaikosaponin K	C48H80O18	M+H	1.586392	945.54174	945.54324	22.063	POS
272	Pentagalloylglucose	C41H32O26	M+NH4	1.262848	958.152	958.15321	18.87	POS
273	Jujuboside A	C58H94O26	M+Na	1.219916	1229.59255	1229.59405	22.749	POS
274	Maltol	C6H6O3	M+H-H2O	0.550312	109.02899	109.02905	7.666	POS
275	p-Aminophenol	C6H7NO	M+H	4.542975	110.06004	110.06054	0.871	POS

276	3-Hydroxy-4-aminopyridine	C5H6N2O	M+H	4.412217	111.05529	111.05578	0.78	POS
277	Cyclohexaneacetic acid	C8H14O2	M-H2O-H	-4.468602	123.08099	123.08044	19.187	NEG
278	Ketoisoleucine	C6H10O3	M-H	-8.058535	129.05572	129.05468	9.01	NEG
279	2-Methylsuccinic acid	C5H8O4	M-H	-8.852598	131.03498	131.03382	6.358	NEG
280	3-Methylbenzaldehyde	C8H8O	M+H	2.14761	121.06479	121.06505	21.877	POS
281	Vanillyl alcohol	C8H10O3	M-H2O-H	-4.220828	135.0446	135.04403	9.28	NEG
282	2'-Hydroxyacetophenone	C8H8O2	M-H	-7.47898	135.04515	135.04414	21.678	NEG
283	Threitol	C4H10O4	M+H	1.543897	123.06518	123.06537	0.796	POS
284	4-Hydroxybenzylamine	C7H9NO	M+H	4.110394	124.07569	124.0762	1.857	POS
285	5,6-Dihydropyridin-2(1H)-one	C5H7NO	M+FA-H	-6.898933	142.05096	142.04998	5.194	NEG
286	N-Hydroxypipecolic acid	C6H11NO3	M-H	-6.316522	144.06662	144.06571	2.114	NEG
287	6-Methyluracil	C5H6N2O2	M+H	2.754817	127.0502	127.05055	3.288	POS
288	Guvacine	C6H9NO2	M+H	2.498622	128.0706	128.07092	0.8	POS
289	Furaneol	C6H8O3	M+H	3.874329	129.05462	129.05512	8.345	POS
290	3,4-Dihydrocoumarin	C9H8O2	M-H	-6.120569	147.04515	147.04425	16.34	NEG
291	Mevalonic acid	C6H12O4	M-H	-6.595666	147.06628	147.06531	2.952	NEG
292	NSC 16590	C4H9NO2	M+FA-H	-8.037199	148.06153	148.06034	0.685	NEG
293	Methyl Salicylate	C8H8O3	M-H	-6.753175	151.04007	151.03905	13.689	NEG

294	3-Amino-4-methylpentanoic acid	C6H13NO2	M+H	2.119576	132.1019	132.10218	2.384	POS
295	3,4,5-Trihydroxybenzaldehyde	C7H6O4	M-H	-5.489503	153.01933	153.01849	6.225	NEG
296	Iminodiacetic acid	C4H7NO4	M+H	0.969825	134.04478	134.04491	0.714	POS
297	2-Methylcyclohexanone	C7H12O	M+FA-H	-4.201493	157.08701	157.08635	20.356	NEG
298	gamma-Hexalactone	C6H10O2	M+FA-H	-4.589282	159.06628	159.06555	11.998	NEG
299	2-Hydroxyoctanoic acid	C8H16O3	M-H	-6.473807	159.10267	159.10164	21.291	NEG
300	4-Hydroxyacetophenone	C8H8O2	M+H	2.480671	137.05971	137.06005	13.491	POS
301	Hydroxytyrosol	C8H10O3	M+H-H2O	-1.532172	137.06029	137.06008	7.91	POS
302	Methyl nicotinate	C7H7NO2	M+H	3.332007	138.05495	138.05541	9.675	POS
303	Umbelliferone	C9H6O3	M-H	-5.961829	161.02442	161.02346	14.991	NEG
304	7-Hydroxychromone	C9H6O3	M-H	-4.409269	161.02442	161.02371	12.768	NEG
305	p-Coumaric acid	C9H8O3	M-H	-6.992146	163.04007	163.03893	14.3	NEG
306	2',5'-dihydroxy-4'-methoxyacetophenone	C9H10O4	M-H2O-H	-2.330723	163.03952	163.03914	17.945	NEG
307	Isonicotinamide	C6H6N2O	M+NH4	1.213576	140.08184	140.08201	1.109	POS
308	DL-3-Phenyllactic acid	C9H10O3	M-H	-6.664416	165.05572	165.05462	21.175	NEG
309	Coumaran	C8H8O	M+FA-H	-6.603831	165.05571	165.05462	18.471	NEG
310	Tropolone	C7H6O2	M+FA-H	-3.8914	167.03498	167.03433	10.972	NEG

311	4-Hydroxymandelic acid	C8H8O4	M-H	-3.951268	167.03498	167.03432	2.554	NEG
312	2-Hydroxy-4-methoxybenzoic acid	C8H8O4	M-H	-5.627564	167.03498	167.03404	20.189	NEG
313	Demethylcantharidate	C8H10O5	M-H2O-H	-0.718415	167.03443	167.03431	7.766	NEG
314	Isovanillic acid	C8H8O4	M-H	-3.8914	167.03498	167.03433	12.602	NEG
315	Guvacoline	C7H11NO2	M+H	1.970634	142.08625	142.08653	2.084	POS
316	Arecaidine	C7H11NO2	M+H	1.618735	142.08625	142.08648	0.814	POS
317	Orcinol	C7H8O2	M+FA-H	-4.614002	169.05063	169.04985	11.001	NEG
318	5-Hydroxymethylfurfural	C6H6O3	M+FA-H	-4.443668	171.02989	171.02913	6.416	NEG
319	trans-4-Hydroxy-2-nonenoic acid	C9H16O3	M-H	-3.506666	171.10267	171.10207	20.967	NEG
320	Aminoadipic acid	C6H11NO4	M+H-H2O	-1.388252	144.06611	144.06591	5.077	POS
321	trans-Aconitic acid	C6H6O6	M-H	-3.814827	173.00916	173.0085	2.534	NEG
322	Cyclohexanecarboxylic acid	C7H12O2	M+FA-H	-4.102104	173.08193	173.08122	20.44	NEG
323	Hygric acid	C6H11NO2	M+FA-H	-5.399904	174.07718	174.07624	0.728	NEG
324	Indole-3-carboxaldehyde	C9H7NO	M+H	2.32781	146.06004	146.06038	16.63	POS
325	4-Hydroxyhygric acid	C6H11NO3	M+H	0.616096	146.08117	146.08126	0.748	POS
326	2-(1-Ethoxyethoxy)ethyl)benzene	C12H18O2	M-H2O-H	-2.341355	175.11229	175.11188	28.851	NEG
327	Coumarin	C9H6O2	M+H	0.816082	147.04406	147.04418	18.658	POS

328	4(3H)-Quinazolinone	C8H6N2O	M+H	0.544013	147.05529	147.05537	10.788	POS
329	α-L-Rhamnose	C6H12O5	M+H-H2O	-1.767916	147.06577	147.06551	0.748	POS
330	Jasmone	C11H16O	M+H-H2O	-4.41824	147.11742	147.11677	26.609	POS
331	Syringic acid	C9H10O5	M-H2O-H	-1.731511	179.03443	179.03412	12.81	NEG
332	Phthalide	C8H6O2	M+FA-H	-4.803531	179.03498	179.03412	14.32	NEG
333	Chelidonic acid	C7H4O6	M-H	-3.989213	182.99351	182.99278	1.445	NEG
334	Methyl gallate	C8H8O5	M-H	-3.168881	183.0299	183.02932	10.345	NEG
335	Isophorone	C9H14O	M+FA-H	-4.532976	183.10266	183.10183	21.155	NEG
336	2-Amino-4-methoxyphenol	C7H9NO2	M+FA-H	-4.78101	184.06153	184.06065	3.501	NEG
337	4'-Hydroxy-3'-methylacetophenone	C9H10O2	M+H	2.647685	151.07536	151.07576	18.931	POS
338	4-Allylcatechol	C9H10O2	M+H	0.397153	151.07536	151.07542	21.712	POS
339	3,4-Dimethylbenzoic acid	C9H10O2	M+H	2.449109	151.07536	151.07573	22.19	POS
340	Benzylurea	C8H10N2O	M+H	3.176986	151.08659	151.08707	11.223	POS
341	Guanine	C5H5N5O	M+H	-0.197295	152.05669	152.05666	1.372	POS
342	3-Hydroxycapric acid	C10H20O3	M-H	-5.023139	187.13397	187.13303	24.211	NEG
343	Hydroxycitric acid lactone	C6H6O7	M-H	1.005269	189.00408	189.00427	0.674	NEG
344	2,4,6-Trihydroxybenzoic acid	C7H6O5	M+H-H2O	-2.679409	153.01882	153.01841	6.402	POS
345	Isovanillin	C8H8O3	M+H	0.718698	153.05462	153.05473	14.093	POS

346	2-Acetonaphthone	C12H10O	M+H-H2O	-2.939823	153.07046	153.07001	26.344	POS
347	5,7-Dihydroxy-4-methylcoumarin	C10H8O4	M-H	-3.978329	191.03498	191.03422	17.819	NEG
348	Isolimonenol	C10H16O	M+H	1.697933	153.12739	153.12765	23.807	POS
349	trans,trans-2,4-Decadienal	C10H16O	M+H	0.653051	153.12739	153.12749	30.386	POS
350	1-Methyl-6-oxo-1,6-dihydropyridine-3-carboxylic acid	C7H7NO3	M+H	2.661476	154.04987	154.05028	7.13	POS
351	5-Aminosalicylic Acid	C7H7NO3	M+H	0.908797	154.04987	154.05001	1.594	POS
352	trans-Isoferulic acid	C10H10O4	M-H	-3.056193	193.05063	193.05004	17.062	NEG
353	Propyl gallate	C10H12O5	M-H2O-H	0.2072	193.05008	193.05012	19.288	NEG
354	Glucosamic acid	C6H13NO6	M-H	-3.400887	194.06701	194.06635	0.679	NEG
355	2-Carboxybenzaldehyde	C8H6O3	M+FA-H	-2.922629	195.02989	195.02932	11.146	NEG
356	3,4-O-Isopropylidene-shikimic acid	C10H14O5	M-H2O-H	0.922766	195.06573	195.06591	11.894	NEG
357	(E)-p-Coumaryl alcohol	C9H10O2	M+FA-H	-1.99932	195.06628	195.06589	13.332	NEG
358	4-vinylguaiacol	C9H10O2	M+FA-H	-1.845527	195.06628	195.06592	21.049	NEG
359	Umbellulone	C10H14O	M+FA-H	-4.202916	195.10266	195.10184	24.422	NEG
360	Cyclo(Gly-L-Pro)	C7H10N2O2	M+H	0.58034	155.0815	155.08159	5.496	POS

361	Methyl 3-O-methylgallate	C9H10O5	M-H	-1.979238	197.04555	197.04516	15.445	NEG
362	Vanillylmandelic acid	C9H10O5	M-H	-1.62399	197.04555	197.04523	4.554	NEG
363	Danshensu	C9H10O5	M-H	-2.029987	197.04555	197.04515	7.092	NEG
364	Carveol	C10H16O	M+FA-H	-2.079969	197.11831	197.1179	20.903	NEG
365	2,6-Dimethylhydroquinone	C8H10O2	M+NH4	1.985882	156.1019	156.10221	10.008	POS
366	D-(+)-Malic acid	C4H6O5	M+Na	2.165457	157.01074	157.01108	0.928	POS
367	Bergaptol	C11H6O4	M-H	-3.482252	201.01933	201.01863	20.778	NEG
368	Alanyleucine	C9H18N2O3	M-H	-1.591055	201.12447	201.12415	6.882	NEG
369	Indole-3-lactic acid	C11H11NO3	M-H	-1.862137	204.06662	204.06624	15.965	NEG
370	8-Aminooctanoic acid	C8H17NO2	M+H	2.18568	160.1332	160.13355	8.324	POS
371	delta-Valerobetaine	C8H17NO2	M+H	-1.124064	160.1332	160.13302	0.871	POS
372	Eugenol acetate	C12H14O3	M-H	-2.145431	205.08702	205.08658	27.993	NEG
373	2-Methylquinazolin-4-ol	C9H8N2O	M+H	1.490027	161.07094	161.07118	9.706	POS
374	Daminozide	C6H12N2O3	M+H	0.49661	161.09207	161.09215	1.756	POS
375	Indole-3-carboxylic acid	C9H7NO2	M+FA-H	-2.378111	206.04588	206.04539	16.737	NEG
376	Ethyl Caffeic acid	C11H12O4	M-H	-1.69028	207.06628	207.06593	21.217	NEG
377	1-Deoxymannojirimycin	C6H13NO4	M+H	2.193895	164.09173	164.09209	0.764	POS
378	Flopropione	C9H10O4	M+H-H2O	-0.848201	165.05521	165.05507	20.77	POS
379	(-)-Fucose	C6H12O5	M+H	2.362552	165.07575	165.07614	0.764	POS

380	D-altrofurano-heptulose-3	C7H14O7	M-H	-4.11352	209.06668	209.06582	0.844	NEG
381	Hinokitiol	C10H12O2	M+FA-H	-2.200094	209.08193	209.08147	22.333	NEG
382	4-Methyl-5,6,7,8-tetrahydroquinoline	C10H13N	M+NH4	0.968883	165.13862	165.13878	9.911	POS
383	3,4-Dimethoxybenzyl alcohol	C9H12O3	M+FA-H	-1.6426	213.07684	213.07649	14.196	NEG
384	Caffeoyl alcohol	C9H10O3	M+H	0.957681	167.07027	167.07043	10.704	POS
385	Methyl 4-hydroxyphenylacetate	C9H10O3	M+H	2.992753	167.07027	167.07077	17.36	POS
386	3-Methoxyphenylacetic acid	C9H10O3	M+H	1.137246	167.07027	167.07046	19.305	POS
387	(-)-3-Dehydroshikimic acid	C7H8O5	M+FA-H	0.645056	217.03537	217.03551	0.709	NEG
388	2,6-Dimethoxy-1,4-benzoquinone	C8H8O4	M+H	1.064777	169.04953	169.04971	11.451	POS
389	Purpurogallin	C11H8O5	M-H	-1.963202	219.0299	219.02947	20.189	NEG
390	Shikimic acid	C7H10O5	M+FA-H	-2.921694	219.05102	219.05038	0.863	NEG
391	D-N-Acetylgalactosamine	C8H15NO6	M-H	-4.180248	220.08266	220.08174	0.766	NEG
392	D-Glucuronic acid lactone	C6H8O6	M+FA-H	-3.121744	221.03029	221.0296	0.784	NEG
393	6-hydroxy-4-methylcoumarin	C10H8O3	M+FA-H	-0.588114	221.04554	221.04541	16.758	NEG
394	Methyl 3-(3’,4’-dimethoxyphenyl)propenoate	C12H14O4	M-H	-1.628356	221.08193	221.08157	23.845	NEG

395	Glycylglycine	C4H8N2O3	M+K	1.988111	171.01665	171.01699	0.693	POS
396	Sorbic acid	C6H8O2	2M-H	-0.627528	223.09758	223.09744	17.041	NEG
397	3,4-Dimethoxyphenol	C8H10O3	M+NH4	3.370196	172.09682	172.0974	12.887	POS
398	5-Acetylsalicylic acid	C9H8O4	M+FA-H	-0.888729	225.04046	225.04026	15.548	NEG
399	4-Vinylsyringol	C10H12O3	M+FA-H	-1.599454	225.07684	225.07648	21.554	NEG
400	Prolylglycine	C7H12N2O3	M+H	0.866591	173.09207	173.09222	0.814	POS
401	Osthenol	C14H14O3	M-H	-1.309546	229.08702	229.08672	24.51	NEG
402	D-Kavain	C14H14O3	M-H	-1.13494	229.08702	229.08676	25.142	NEG
403	Isoniazid	C6H7N3O	M+K	2.783742	176.02207	176.02256	0.605	POS
404	8-Hydroxybergapten	C12H8O5	M-H	-2.164222	231.0299	231.0294	20.839	NEG
405	Kifunensine	C8H12N2O6	M-H	2.639981	231.06226	231.06287	0.662	NEG
406	4-Methylumbelliferone	C10H8O3	M+H	1.355514	177.05462	177.05486	18.325	POS
407	10-Hydroxydecanoic acid	C10H20O3	M+FA-H	-1.458355	233.13944	233.1391	21.238	NEG
408	Herniarin	C10H8O3	M+H	2.993427	177.05462	177.05515	20.749	POS
409	4-Methylesculetin	C10H8O4	M+FA-H	-1.096859	237.04046	237.0402	15.002	NEG
410	5,7-Dihydroxycoumarin	C9H6O4	M+H	2.178359	179.03388	179.03427	13.616	POS
411	Daphnetin	C9H6O4	M+H	2.066648	179.03388	179.03425	12.99	POS
412	3,4,5-Trimethoxycinnamic acid	C12H14O5	M-H	-1.940299	237.07685	237.07639	20.398	NEG

413	2-Methoxycinnamic acid	C10H10O3	M+H	0.055844	179.07027	179.07028	21.712	POS
414	4-Methoxycinnamic acid	C10H10O3	M+H	2.401292	179.07027	179.0707	21.044	POS
415	7-Hydroxyflavanone	C15H12O3	M-H	-0.878399	239.07137	239.07116	23.908	NEG
416	Methyl beta-D-Galactopyranoside	C7H14O6	M+FA-H	-0.627412	239.07724	239.07709	0.955	NEG
417	3,4,5-Trimethoxyhydrocinnamic acid	C12H16O5	M-H	-1.672993	239.0925	239.0921	19.959	NEG
418	4,6-Dioxoheptanoic acid	C7H10O4	M+Na	-1.988433	181.04713	181.04677	7.419	POS
419	Caffeic acid	C9H8O4	M+H	2.540741	181.04953	181.04999	11.911	POS
420	Coniferyl alcohol	C10H12O3	M+H	2.043229	181.08592	181.08629	20.333	POS
421	Ethyl gallate	C9H10O5	M+FA-H	-2.345187	243.05102	243.05045	14.757	NEG
422	2,6-Dimethoxybenzoic acid	C9H10O4	M+H	1.474885	183.06518	183.06545	15.129	POS
423	Arglabin	C15H18O3	M-H	-0.897526	245.11832	245.1181	27.974	NEG
424	Zederone	C15H18O3	M-H	-0.652746	245.11832	245.11816	28.851	NEG
425	Phosphorylcholine	C5H14NO4P	M+H	0.271631	184.07332	184.07337	0.732	POS
426	Kanshone C	C15H20O3	M-H	0.040464	247.13397	247.13398	26.761	NEG
427	Bisabolangelone	C15H20O3	M-H	0.080928	247.13397	247.13399	25.353	NEG
428	Methyl 2,6-dihydroxybenzoate	C8H8O4	M+NH4	1.719727	186.07608	186.0764	21.253	POS
429	Griffonilide	C8H8O4	M+NH4	2.095917	186.07608	186.07647	5.908	POS

430	Trolox	C14H18O4	M-H	-1.966977	249.11323	249.11274	22.312	NEG
431	Nardosinone	C15H22O3	M-H	0.200683	249.14962	249.14967	27.654	NEG
432	Angelicin	C11H6O3	M+H	1.069296	187.03897	187.03917	22.437	POS
433	D-(+)-Fucose	C6H12O5	M+Na	-5.132107	187.05769	187.05673	0.605	POS
434	7,8-Dimethoxycoumarin	C11H10O4	M+FA-H	1.513606	251.05611	251.05649	20.356	NEG
435	Citropten	C11H10O4	M+H-H2O	-2.168679	189.05521	189.0548	23.223	POS
436	8-acetyl dimethoxycoumarin	C11H8O3	M+H	1.586843	189.05462	189.05492	20.624	POS
437	2-Hydroxy-3-(hydroxymethyl)anthraquinone	C15H10O4	M-H	-2.331549	253.05063	253.05004	21.554	NEG
438	Senkyunolide G	C12H16O3	M+FA-H	0.474106	253.10814	253.10826	23.211	NEG
439	Castanospermine	C8H15NO4	M+H	0.631222	190.10738	190.1075	0.89	POS
440	Pinocembrin	C15H12O4	M-H	0.744904	255.06628	255.06647	26.357	NEG
441	4-Methylherniarin	C11H10O3	M+H	3.035532	191.07027	191.07085	22.52	POS
442	2,6-Diaminoheptanedioic acid	C7H14N2O4	M+H	1.622165	191.10263	191.10294	0.834	POS
443	Cytisinicline	C11H14N2O	M+H	-0.523237	191.11789	191.11779	3.058	POS
444	2′-O-Methyluridine	C10H14N2O6	M-H	1.322556	257.07791	257.07825	6.818	NEG
445	Ononetin	C15H14O4	M-H	-1.244739	257.08193	257.08161	25.615	NEG
446	Lawsone	C10H6O3	M+NH4	2.34295	192.06552	192.06597	18.826	POS
447	Benzoin	C14H12O2	M+FA-H	-1.166943	257.08193	257.08163	23.211	NEG

448	5-Hydroxyferulic acid	C10H10O5	M+H-H2O	0	193.05012	193.05012	12.908	POS
449	Scopoletin	C10H8O4	M+H	1.087804	193.04953	193.04974	15.856	POS
450	Caffeine	C8H10N4O2	M+H	0.871403	195.08765	195.08782	11.806	POS
451	Zingerone	C11H14O3	M+H	1.691427	195.10157	195.1019	18.87	POS
452	Peucedanol	C14H16O5	M-H	-0.380094	263.0925	263.0924	17.966	NEG
453	Granilin	C15H20O4	M-H	0.494054	263.12888	263.12901	20.274	NEG
454	Tubercidin	C11H14N4O4	M-H	-3.885411	265.09423	265.0932	5.216	NEG
455	Coumestrol	C15H8O5	M-H	-0.861327	267.0299	267.02967	22.014	NEG
456	Asaraldehyde	C10H12O4	M+H	1.014812	197.08083	197.08103	19.964	POS
457	Ethyl Vanillate	C10H12O4	M+H	0.659628	197.08083	197.08096	21.877	POS
458	Ethyl ferulic acid	C12H14O4	M+FA-H	2.84551	267.08741	267.08817	23.586	NEG
459	Ftaxilide	C16H15NO3	M-H	-1.715791	268.09792	268.09746	22.227	NEG
460	Baicalein	C15H10O5	M-H	2.155769	269.04555	269.04613	22.688	NEG
461	Emodin	C15H10O5	M-H	0	269.04555	269.04555	29.21	NEG
462	Norwogonin	C15H10O5	M-H	2.750464	269.04555	269.04629	22.333	NEG
463	Alloimperatorin	C16H14O4	M-H	-1.375046	269.08193	269.08156	27.929	NEG
464	Echinatin	C16H14O4	M-H	-0.631778	269.08193	269.08176	21.891	NEG
465	Vomifoliol	C13H20O3	M+FA-H	1.077508	269.13944	269.13973	15.298	NEG
466	Harmol	C12H10N2O	M+H	1.205506	199.08659	199.08683	12.845	POS
467	Butein	C15H12O5	M-H	1.80771	271.0612	271.06169	21.175	NEG
468	Guaiazulene	C15H18	M+H	-0.150642	199.14813	199.1481	34.078	POS

469	Loureirin C	C16H16O4	M-H	0.590193	271.09758	271.09774	21.891	NEG
470	Resveratrol	C14H12O3	M+FA-H	0.878874	273.07684	273.07708	19.98	NEG
471	Ethyl coumarin-3-carboxylate	C12H10O4	M+H-H2O	-1.143964	201.05521	201.05498	22.437	POS
472	N-Formylcytisine	C12H14N2O2	M+H-H2O	-0.546984	201.10283	201.10272	9.193	POS
473	Harmalol	C12H12N2O	M+H	2.734927	201.10224	201.10279	11.512	POS
474	Camphoric acid	C10H16O4	M+H	2.038664	201.11213	201.11254	17.149	POS
475	Xanthotoxol	C11H6O4	M+H	2.561149	203.03388	203.0344	19.614	POS
476	3',4'-Dimethoxyflavone	C17H14O4	M-H	-7.969207	281.08193	281.07969	25.037	NEG
477	Allitol	C6H14O6	M+Na	0	205.06826	205.06826	0.748	POS
478	N-Methylcytisine	C12H16N2O	M+H	0.77998	205.13354	205.1337	2.711	POS
479	Bufotenin	C12H16N2O	M+H	0.828728	205.13354	205.13371	6.628	POS
480	Morin	C15H10O7	M-H2O-H	3.639264	283.02426	283.02529	20.818	NEG
481	Spinacine	C7H9N3O2	M+K	3.591664	206.03263	206.03337	0.585	POS
482	Prunetin	C16H12O5	M-H	-1.201154	283.0612	283.06086	27.167	NEG
483	α-Acetamidocinnamic acid	C11H11NO3	M+H	0.048525	206.08117	206.08118	14.532	POS
484	Datiscetin	C15H10O6	M-H	1.894468	285.04046	285.041	21.554	NEG
485	Fisetin	C15H10O6	M-H	1.964633	285.04046	285.04102	20.023	NEG
486	Kaempferol	C15H10O6	M-H	0.210496	285.04046	285.04052	22.132	NEG
487	Sakuranetin	C16H14O5	M-H	1.438209	285.07685	285.07726	26.313	NEG

488	Oxypeucedanin	C16H14O5	M-H	1.473287	285.07685	285.07727	25.684	NEG
489	7-Methoxy-4-methyl-coumarin-8-ol	C11H10O4	M+H	1.786877	207.06518	207.06555	19.78	POS
490	Isookanin	C15H12O6	M-H	1.706983	287.05611	287.0566	16.089	NEG
491	Okanin	C15H12O6	M-H	1.567638	287.05611	287.05656	20.21	NEG
492	(±)-Equol	C15H14O3	M+FA-H	-1.532607	287.09249	287.09205	21.741	NEG
493	4'-Methoxyresveratrol	C15H14O3	M+FA-H	-0.557312	287.09249	287.09233	23.336	NEG
494	Ferulic acid methyl ester	C11H12O4	M+H	2.96536	209.08083	209.08145	21.567	POS
495	Genipin	C11H14O5	M+H-H2O	-1.960959	209.08142	209.08101	17.986	POS
496	3,4-Dimethoxycinnamic acid	C11H12O4	M+H	3.539301	209.08083	209.08157	20.292	POS
497	Piceatannol	C14H12O4	M+FA-H	2.456138	289.07176	289.07247	18.112	NEG
498	Gnetol	C14H12O4	M+FA-H	2.352357	289.07176	289.07244	17.398	NEG
499	Dehydrocrenatidine	C15H14N2O2	M+Cl	1.037793	289.07493	289.07523	20.438	NEG
500	Royal Jelly acid	C10H18O3	M+Na	7.603479	209.11481	209.1164	20.589	POS
501	Megastigm-7-ene-3,5,6,9-tetraol	C13H24O4	M+FA-H	0.622481	289.16566	289.16584	12.518	NEG
502	Crenulatin	C11H20O6	M+FA-H	-1.194033	293.12419	293.12384	10.303	NEG
503	Mannoheptulose	C7H14O7	M+H	3.505759	211.08123	211.08197	0.585	POS
504	N2-Methylguanosine	C11H15N5O5	M-H	1.046943	296.10004	296.10035	7.242	NEG
505	D-Glucuronic acid	C6H10O7	M+NH4	1.273126	212.07648	212.07675	0.605	POS
506	Hedysarimcoumestan B	C16H10O6	M-H	1.279287	297.04046	297.04084	21.912	NEG

507	Pendulone	C17H16O6	M-H2O-H	1.009842	297.0763	297.0766	22.73	NEG
508	Quebrachitol	C7H14O6	M+NH4	1.650065	212.11286	212.11321	0.78	POS
509	Perseitol	C7H16O7	M+H	2.346351	213.09688	213.09738	0.585	POS
510	Dihydrosinapyl alcohol	C11H16O4	M+H	3.003114	213.11213	213.11277	15.318	POS
511	Chrysin	C15H10O4	M+FA-H	-0.601894	299.05611	299.05593	26.357	NEG
512	7,8-Dihydroxyflavone	C15H10O4	M+FA-H	-0.300947	299.05611	299.05602	20.946	NEG
513	Rhamnocitrin	C16H12O6	M-H	-0.735648	299.05611	299.05589	26.983	NEG
514	3-oxo-2-pentylcyclopentaneacetic acid	C12H20O3	M+H	0.938313	213.14852	213.14872	22.977	POS
515	Dehydroabietic acid	C20H28O2	M-H	0.501334	299.20165	299.2018	32.994	NEG
516	Ellagic acid	C14H6O8	M-H	0.598009	300.99899	300.99917	16.998	NEG
517	Quercetin	C15H10O7	M-H	0.797248	301.03538	301.03562	21.04	NEG
518	Phenyl β-D-glucopyranoside	C12H16O6	M+FA-H	1.062795	301.09289	301.09321	9.773	NEG
519	Isoalantolactone	C15H20O2	M+H-H2O	-0.976092	215.14363	215.14342	28.498	POS
520	Taxifolin	C15H12O7	M-H	-1.286912	303.05103	303.05064	16.717	NEG
521	3-Methyluridine	C10H14N2O6	M+FA-H	-0.428925	303.08339	303.08326	6.204	NEG
522	4-Hydroxymephenytoin	C12H14N2O3	M+H-H2O	-0.552746	217.09774	217.09762	16.128	POS
523	3,5-Dimethoxyphenol	C8H10O3	2M-H	-5.307394	307.11871	307.11708	18.323	NEG
524	Eicosadienoic acid	C20H36O2	M-H	1.301811	307.26425	307.26465	35.84	NEG

525	7-acetoxy-4-methylcoumarin	C12H10O4	M+H	9.449242	219.06518	219.06725	21.489	POS
526	Teucvidin	C19H20O5	M-H2O-H	1.811637	309.11268	309.11324	25.596	NEG
527	Lupulone C	C15H20O4	M+FA-H	2.42613	309.13436	309.13511	25.596	NEG
528	Abrine	C12H14N2O2	M+H	2.099375	219.1128	219.11326	9.315	POS
529	Paullinic acid	C20H38O2	M-H	-0.646663	309.2799	309.2797	36.726	NEG
530	Vasicinone	C11H10N2O2	M+NH4	1.862722	220.10805	220.10846	12.226	POS
531	trans-Zeatin	C10H13N5O	M+H	-4.99729	220.11929	220.11819	8.862	POS
532	Palmitoylglycine	C18H35NO3	M-H	0.320252	312.25442	312.25452	33.71	NEG
533	Wedelolactone	C16H10O7	M-H	-0.54307	313.03538	313.03521	21.008	NEG
534	Norbergenin	C13H14O9	M-H	-1.724928	313.05651	313.05597	7.745	NEG
535	Pratol	C16H12O4	M+FA-H	1.565136	313.07176	313.07225	22.71	NEG
536	Ermanin	C17H14O6	M-H	1.533195	313.07176	313.07224	27.952	NEG
537	Naringenin trimethyl ether	C18H18O5	M-H	-6.930513	313.10815	313.10598	28.851	NEG
538	Fraxinol	C11H10O5	M+H	2.869182	223.0601	223.06074	17.945	POS
539	7,8-Dihydroxy-3-(4-hydroxyphenyl)-4H-chromen-4-one	C15H10O5	M+FA-H	2.602753	315.05102	315.05184	17.503	NEG
540	Genistein	C15H10O5	M+FA-H	2.793198	315.05102	315.0519	22.123	NEG
541	Higenamine	C16H17NO3	M+FA-H	-6.484899	316.11904	316.11699	10.282	NEG
542	2-hydroxypinocembrin	C15H12O5	M+FA-H	-0.283852	317.06667	317.06658	22.5	NEG

543	Arbutin	C12H16O7	M+FA-H	0.31537	317.0878	317.0879	3.121	NEG
544	Helicid	C13H16O7	M+Cl	0.940265	319.059	319.0593	8.23	NEG
545	Piperine	C17H19NO3	M+Cl	-0.156198	320.1059	320.10585	28.181	NEG
546	8-Prenyldaidzein	C20H18O4	M-H	0.685117	321.11323	321.11345	28.181	NEG
547	Glabridin	C20H20O4	M-H	1.176001	323.12888	323.12926	29.688	NEG
548	Methyl beta-carboline-1-carboxylate	C13H10N2O2	M+H	2.113779	227.0815	227.08198	19.49	POS
549	N-trans-sinapoyltyramine	C19H21NO5	M-H2O-H	3.239505	324.12358	324.12463	20.61	NEG
550	Phenprocoumon	C18H16O3	M+FA-H	-1.138083	325.10814	325.10777	28.769	NEG
551	Spinacetin	C17H14O8	M-H2O-H	3.699735	327.05048	327.05169	20.992	NEG
552	Bergenin	C14H16O9	M-H	0.794932	327.07216	327.07242	11.126	NEG
553	Xylobiose	C10H18O9	M+FA-H	-0.489157	327.09328	327.09312	0.805	NEG
554	(-?)?-Maackiain	C16H12O5	M+FA-H	-1.79295	329.06667	329.06608	24.909	NEG
555	Visnagin	C13H10O4	M+H	1.514724	231.06518	231.06553	21.712	POS
556	Gastrodin	C13H18O7	M+FA-H	1.087273	331.10345	331.10381	6.352	NEG
557	(1R,9S)-11-Acetyl-7,11-diazatricyclo[7.3.1.02,7]trideca-2,4-dien-6-one	C13H16N2O2	M+H	-0.514738	233.12845	233.12833	10.413	POS
558	Glaucocalyxin A	C20H28O4	M-H	-0.090582	331.19148	331.19145	21.554	NEG
559	20-Deoxyingenol	C20H28O4	M-H	0.211358	331.19148	331.19155	25.959	NEG
560	Dehydroandrographolide	C20H28O4	M-H	-0.120776	331.19148	331.19144	24.125	NEG

561	5-Hydroxyprocurcumenol	C15H22O3	M+H-H2O	-0.643351	233.15419	233.15404	20.353	POS
562	(Rac)-Byakangelicin	C17H18O7	M-H	-1.531081	333.09798	333.09747	21.238	NEG
563	Minimolide F	C19H26O5	M-H	-5.852855	333.17075	333.1688	26.856	NEG
564	Lathyrol	C20H30O4	M-H	-1.440545	333.20713	333.20665	27.442	NEG
565	5-O-Caffeoylshikimic acid	C16H16O8	M-H	1.522037	335.07724	335.07775	14.364	NEG
566	Glabrone	C20H16O5	M-H	-0.298425	335.0925	335.0924	29.572	NEG
567	5-O-Methylvisamminol	C16H18O5	M+FA-H	0.805697	335.11362	335.11389	21.258	NEG
568	2-O-beta-D-Glucopyranosyl-L-ascorbic acid	C12H18O11	M-H	-1.661338	337.07764	337.07708	1.172	NEG
569	5-O-(E)-p-Coumaroylquinic acid	C16H18O8	M-H	2.432564	337.09289	337.09371	13.479	NEG
570	Lupiwighteone	C20H18O5	M-H	1.75018	337.10815	337.10874	29.285	NEG
571	6-Prenylapigenin	C20H18O5	M-H	1.245891	337.10815	337.10857	29.563	NEG
572	Demethoxycurcumin	C20H18O5	M-H	1.512868	337.10815	337.10866	28.707	NEG
573	Sauchinone	C20H20O6	M-H2O-H	2.818091	337.1076	337.10855	30.281	NEG
574	Kinetin	C10H9N5O	M+Na	6.594701	238.06993	238.0715	10.89	POS
575	2(S)-6-Prenylnaringenin	C20H20O5	M-H	1.326949	339.1238	339.12425	29.793	NEG
576	Prunasin	C14H17NO6	M+FA-H	-0.029403	340.10379	340.10378	13.248	NEG
577	3'-Methoxycoumestrol	C16H10O6	M+FA-H	-1.282627	343.04594	343.0455	22.5	NEG

578	3-Galloylquinic acid	C14H16O10	M-H	1.748929	343.06707	343.06767	6.075	NEG
579	Picein	C14H18O7	M+FA-H	0.145729	343.10345	343.1035	9.814	NEG
580	Diosbulbin B	C19H20O6	M-H	1.136633	343.11871	343.1191	20.946	NEG
581	Isomalt	C12H24O11	M-H	-1.457197	343.12459	343.12409	0.825	NEG
582	Dihydrocatalpol	C15H24O10	M-H2O-H	0.231804	345.11856	345.11864	2.575	NEG
583	Moracin M	C14H10O4	M+H	-6.130043	243.06518	243.06369	20.665	POS
584	Continentalic acid	C20H30O2	M+FA-H	-5.241592	347.22278	347.22096	34.743	NEG
585	Huperzine A	C15H18N2O	M+H	0.41127	243.14919	243.14929	10.62	POS
586	Fraxidin	C11H10O5	M+Na	1.346708	245.04204	245.04237	17.254	POS
587	Osthole	C15H16O3	M+H	1.305498	245.11722	245.11754	29.897	POS
588	Batatasin III	C15H16O3	M+H	0.448765	245.11722	245.11733	23.142	POS
589	Paulownin	C20H18O7	M-H2O-H	1.765945	351.08686	351.08748	25.057	NEG
590	Mollicellin I	C21H22O6	M-H2O-H	3.018883	351.12325	351.12431	29.106	NEG
591	Incensole	C20H34O2	M+FA-H	-1.110307	351.25408	351.25369	34.641	NEG
592	Narciclasine	C14H13NO7	M+FA-H	1.846237	352.0674	352.06805	13.376	NEG
593	Magnolioside	C16H18O9	M-H	0.764682	353.08781	353.08808	11.477	NEG
594	1-Caffeoylquinic acid	C16H18O9	M-H	0.566431	353.08781	353.08801	8.083	NEG
595	Gancaonin I	C21H22O5	M-H	0.679618	353.13945	353.13969	31.544	NEG
596	Tetrahydrocurcumin	C21H24O6	M-H2O-H	0.509714	353.1389	353.13908	27.592	NEG
597	Notopterol	C21H22O5	M-H	0.792888	353.13945	353.13973	29.646	NEG
598	Licochalcone D	C21H22O5	M-H	0.424761	353.13945	353.1396	26.689	NEG

599	Xanthohumol	C21H22O5	M-H	0.538031	353.13945	353.13964	30.531	NEG
600	Hypaphorine	C14H18N2O2	M+H	0.485547	247.1441	247.14422	10.476	POS
601	Sophocarpine	C15H22N2O	M+H	1.254144	247.18049	247.1808	7.731	POS
602	Linamarin	C10H17NO6	M+H	0.523955	248.11286	248.11299	5.887	POS
603	6,7-Dimethoxy-2-phenethylchromone	C19H18O4	M+FA-H	0	355.11871	355.11871	27.675	NEG
604	Moracin N	C19H18O4	M+FA-H	-1.661416	355.11871	355.11812	28.181	NEG
605	(Rac)-Myrislignan	C21H26O6	M-H2O-H	3.238027	355.15455	355.1557	29.085	NEG
606	Methyl Eugenol	C11H14O2	2M-H	0.844615	355.19148	355.19178	27.464	NEG
607	Allomatrine	C15H24N2O	M+H	0.441419	249.19614	249.19625	7.93	POS
608	Sophoridine	C15H24N2O	M+H	-0.040129	249.19614	249.19613	7.646	POS
609	Lactobionic acid	C12H22O12	M-H	0.952104	357.10385	357.10419	0.784	NEG
610	Loganic acid	C16H24O10	M-H2O-H	0.560038	357.11856	357.11876	10.77	NEG
611	Mussaenosidic acid	C16H24O10	M-H2O-H	1.17608	357.11856	357.11898	9.773	NEG
612	Scabertopin	C20H22O6	M-H	1.596038	357.13436	357.13493	27.55	NEG
613	Triptonide	C20H22O6	M-H	1.904045	357.13436	357.13504	25.394	NEG
614	Voglibose	C10H21NO7	M+H-H2O	-3.518183	250.1291	250.12822	0.871	POS
615	Glucovanillin	C14H18O8	M+FA-H	-1.448071	359.09837	359.09785	10.089	NEG
616	N-(4-aminobutyl)-3-(3,4-dihydroxyphenyl)propenamide	C13H18N2O3	M+H	0.199093	251.13902	251.13907	8.657	POS
617	Aerugidiol	C15H22O3	M+H	2.269432	251.16417	251.16474	19.8	POS

618	Nodosin	C20H26O6	M-H	-0.221505	361.16566	361.16558	21.678	NEG
619	Oxypeucedanin methanolate	C17H18O6	M+FA-H	2.974317	363.10854	363.10962	23.845	NEG
620	Isosteviol	C20H30O3	M+FA-H	-6.552544	363.21769	363.21531	31.609	NEG
621	Methyl chlorogenate	C17H20O9	M-H	0.381364	367.10346	367.1036	16.425	NEG
622	5-Feruloylquinic acid	C17H20O9	M-H	0.272403	367.10346	367.10356	11.977	NEG
623	Pteryxin	C21H22O7	M-H2O-H	1.00785	367.11816	367.11853	30.572	NEG
624	Gomisin M2	C22H26O6	M-H2O-H	0.326838	367.15455	367.15467	31.178	NEG
625	(±)-Stylopine	C19H17NO4	M+FA-H	-0.271655	368.11396	368.11386	20.377	NEG
626	Epiafzelechin	C15H14O5	M+H-H2O	-1.983807	257.08142	257.08091	15.732	POS
627	Afzelechin	C15H14O5	M+H-H2O	-2.99516	257.08142	257.08065	13.595	POS
628	Pterosin B	C14H18O2	M+K	-3.111704	257.09384	257.09304	23.122	POS
629	Uralenol	C20H18O7	M-H	-0.785699	369.09798	369.09769	28.204	NEG
630	(Rac)-Arnebin 1	C21H22O6	M-H	-0.406356	369.13436	369.13421	26.022	NEG
631	Isobavachalcone	C20H20O4	M+FA-H	-0.921074	369.13436	369.13402	30.614	NEG
632	Xanthohumol D	C21H22O6	M-H	-1.083616	369.13436	369.13396	28.56	NEG
633	5-Hydroxymethyldeoxycytidine monophosphate	C10H15N3O5	M+H	0	258.10845	258.10845	1.655	POS
634	Hirudonucleodisulfide B	C11H10N4O4S2	M+FA-H	5.255887	371.01255	371.0145	11.416	NEG
635	4-O-beta-Glucopyranosyl-cis-coumaric acid	C15H18O8	M+FA-H	1.158722	371.09837	371.0988	9.878	NEG

636	Noricaritin	C20H20O7	M-H	1.482026	371.11363	371.11418	30.718	NEG
637	Isorhapontigenin	C15H14O4	M+H	1.389444	259.09648	259.09684	20.333	POS
638	Moracin P	C19H18O5	M+FA-H	1.643701	371.11362	371.11423	22.374	NEG
639	Methylophiopogonone B	C19H18O5	M+FA-H	1.886215	371.11362	371.11432	29.963	NEG
640	Raspberry ketone glucoside	C16H22O7	M+FA-H	1.913052	371.13475	371.13546	13.018	NEG
641	Salvinorin B	C21H26O7	M-H2O-H	3.179312	371.14946	371.15064	22.584	NEG
642	Pseudolaric Acid C	C21H26O7	M-H2O-H	3.179312	371.14946	371.15064	21.932	NEG
643	Quercetin 3,4'-dimethyl ether	C17H14O7	M+FA-H	-0.266615	375.07215	375.07205	23.251	NEG
644	Bartsioside	C15H22O8	M+FA-H	-0.506492	375.12967	375.12948	11.167	NEG
645	Methyl (-)-shikimate	C8H12O5	2M-H	2.345856	375.12967	375.13055	5.782	NEG
646	Tetrahydrobiopterin	C9H15N5O3	M+Na	4.960116	264.10671	264.10802	0.78	POS
647	Kinsenoside	C10H16O8	M+H	0.94307	265.09179	265.09204	1.211	POS
648	Oxymatrine	C15H24N2O2	M+H	-0.226252	265.19105	265.19099	8.465	POS
649	Rupestonic acid	C15H20O3	M+NH4	-6.762467	266.17507	266.17327	22.549	POS
650	Licochalcone E	C21H22O4	M+FA-H	-0.861281	383.15001	383.14968	28.581	NEG
651	Bavachinin	C21H22O4	M+FA-H	-0.548088	383.15001	383.1498	30.697	NEG
652	Cichoriin	C15H16O9	M+FA-H	-0.441469	385.07763	385.07746	10.003	NEG
653	Secoxyloganin	C17H24O11	M-H2O-H	1.713781	385.11347	385.11413	15.002	NEG
654	Praeruptorin A	C21H22O7	M-H	2.700392	385.12928	385.13032	31.032	NEG
655	Britannilactone	C15H22O4	M+H	-5.764356	267.15909	267.15755	15.9	POS
656	Broussochalcone A	C20H20O5	M+FA-H	2.284947	385.12927	385.13015	27.464	NEG

657	Peucedanocoumarin II	C21H22O7	M-H	2.310912	385.12928	385.13017	30.469	NEG
658	Poriol	C16H14O5	M+H-H2O	-0.260144	269.08142	269.08135	24.244	POS
659	Oxyimperatorin	C16H14O5	M+H-H2O	-0.445962	269.08142	269.0813	24.554	POS
660	Geniposide	C17H24O10	M-H	1.679024	387.12967	387.13032	13.207	NEG
661	Verbenalin	C17H24O10	M-H	2.014829	387.12967	387.13045	13.729	NEG
662	Shanzhiside methyl ester	C17H26O11	M-H2O-H	2.970585	387.12912	387.13027	11.665	NEG
663	Medioresil	C21H24O7	M-H	-0.723243	387.14493	387.14465	21.238	NEG
664	Arisantetralone A	C20H22O5	M+FA-H	-0.697413	387.14492	387.14465	24.059	NEG
665	β-Hydroxyisovalerylshikonin	C21H24O7	M-H	-0.284131	387.14493	387.14482	31.137	NEG
666	Quassin	C22H28O6	M-H	0.129138	387.18131	387.18136	21.891	NEG
667	Nevadensin	C18H16O7	M+FA-H	0.89954	389.0878	389.08815	26.499	NEG
668	Secoxyloganic acid	C16H22O11	M-H	-0.154198	389.10894	389.10888	11.56	NEG
669	Resveratroloside	C20H22O8	M-H	-1.978803	389.12419	389.12342	15.066	NEG
670	Loganin	C17H26O10	M-H	-3.212168	389.14532	389.14407	14.196	NEG
671	Neoquassin	C22H30O6	M-H	1.053451	389.19696	389.19737	22.015	NEG
672	Ilicic acid	C15H24O3	M+NH4	2.516595	270.20637	270.20705	25.91	POS
673	Shanzhiside	C16H24O11	M-H	-2.096519	391.12459	391.12377	8.969	NEG
674	Loureirin D	C16H16O5	M+H-H2O	0.295097	271.09707	271.09715	20.917	POS
675	Xanthoangelol	C25H28O4	M-H	1.431524	391.19148	391.19204	32.907	NEG
676	Blinin	C22H32O6	M-H	-0.178931	391.21261	391.21254	22.312	NEG
677	Aloesin	C19H22O9	M-H	-1.322754	393.11911	393.11859	11.456	NEG

678	Protosappanin A	C15H12O5	M+H	1.245076	273.07575	273.07609	16.232	POS
679	Dimethylcurcumin	C23H24O6	M-H	8.452486	395.15001	395.15335	31.407	NEG
680	17-Hydroxyisolathyrol	C20H30O5	M+FA-H	-4.630479	395.20752	395.20569	22.374	NEG
681	Kaempferol 3-arabinofuranoside	C20H18O10	M-H2O-H	1.754071	399.07161	399.07231	20.189	NEG
682	Neopterin	C9H11N5O4	M+Na	2.789144	276.07032	276.07109	1.574	POS
683	Scopolin	C16H18O9	M+FA-H	1.152613	399.09328	399.09374	11.853	NEG
684	Dehydroglyasperin C	C21H22O5	M+FA-H	0.300643	399.14492	399.14504	28.518	NEG
685	1-(3,4-Dimethoxycinnamoyl)piperidine	C16H21NO3	M+H	-7.821569	276.15942	276.15726	23.454	POS
686	Proxyphylline	C10H14N4O3	M+K	-3.717475	277.06975	277.06872	12.411	POS
687	Shogaol	C17H24O3	M+H	-1.623495	277.17982	277.17937	30.473	POS
688	10-Hydroxymajoroside	C17H24O11	M-H	-1.438761	403.12459	403.12401	11.518	NEG
689	Methyl deacetylasperulosidate	C17H24O11	M-H	-1.637211	403.12459	403.12393	9.03	NEG
690	Feretoside	C17H24O11	M-H	-1.488374	403.12459	403.12399	10.199	NEG
691	Strophanthidin	C23H32O6	M-H	0.595219	403.21261	403.21285	20.418	NEG
692	Oxyresveratrol 3'-o-beta-D-glucopyranoside	C20H22O9	M-H	0.271525	405.11911	405.11922	14.633	NEG
693	Eurycomanone	C20H24O9	M-H	-5.354492	407.13476	407.13258	11.375	NEG
694	Ponicidin	C20H26O6	M+FA-H	-9.77476	407.17114	407.16716	18.218	NEG

695	Secoisolariciresinol	C20H26O6	M+FA-H	0.540313	407.17114	407.17136	20.274	NEG
696	Kushenol A	C25H28O5	M-H	0.908675	407.1864	407.18677	31.756	NEG
697	Rhodojaponin II	C22H34O7	M-H	1.295137	409.22318	409.22371	20.778	NEG
698	2'-O-Methyladenosine	C11H15N5O4	M+H	2.374879	282.11968	282.12035	7.005	POS
699	N-Nornuciferine	C18H19NO2	M+H	2.764498	282.14885	282.14963	20.208	POS
700	Anisomycin	C14H19NO4	M+NH4	-0.882877	283.16523	283.16498	13.198	POS
701	Xylotriose	C15H26O13	M-H	2.517367	413.13006	413.1311	1.337	NEG
702	Neopuerarin B	C21H20O9	M-H	0.457717	415.10346	415.10365	16.383	NEG
703	Brevianamide F	C16H17N3O2	M+H	2.357998	284.13935	284.14002	17.592	POS
704	Oroxylin A	C16H12O5	M+H	-0.21047	285.07575	285.07569	26.95	POS
705	Helicin	C13H16O7	M+H	-0.841819	285.09688	285.09664	10.683	POS
706	Oxysophocarpine	C15H22N2O2	M+Na	-5.365459	285.15735	285.15582	8.386	POS
707	Letrozole	C17H11N5	M+H	-5.277714	286.10872	286.10721	21.979	POS
708	(-)-Coclaurine	C17H19NO3	M+H	-0.629054	286.14377	286.14359	12.804	POS
709	Geniposidic acid	C16H22O10	M+FA-H	-0.930522	419.1195	419.11911	8.781	NEG
710	Luteolin	C15H10O6	M+H	1.358625	287.05501	287.0554	21.044	POS
711	Brazilin	C16H14O5	M+H	1.98543	287.0914	287.09197	12.41	POS
712	Cycloolivil	C20H24O7	M+FA-H	0.90229	421.1504	421.15078	14.757	NEG
713	Sanggenol L	C25H26O6	M-H	1.424618	421.16566	421.16626	32.28	NEG
714	7,8,3',4'-Tetrahydroxyflavanone	C15H12O6	M+H	2.940458	289.07066	289.07151	16.484	POS
715	Kuwanon E	C25H28O6	M-H	0.378089	423.18131	423.18147	31.199	NEG

716	Tectorigenin sulfonate	C16H12O9S	M+FA-H	-8.799619	425.0184	425.01466	20	NEG
717	Rosarin	C20H28O10	M-H	-1.568495	427.16097	427.1603	16.425	NEG
718	(+)-Catechin hydrate	C15H14O6	M+H	-0.652727	291.08631	291.08612	11.139	POS
719	Epicatechin	C15H14O6	M+H	-0.377895	291.08631	291.0862	13.385	POS
720	4'-Methoxypuerarin	C22H22O9	M-H	3.891693	429.11911	429.12078	19.187	NEG
721	7-deoxynarciclasine	C14H13NO6	M+H	-7.737565	292.08156	292.0793	11.202	POS
722	Piperlotine C	C16H21NO4	M+H	-0.958398	292.15433	292.15405	22.416	POS
723	Isovitexin	C21H20O10	M-H	-1.786135	431.09837	431.0976	17.357	NEG
724	Sophoricoside	C21H20O10	M-H	-2.087691	431.09837	431.09747	19.208	NEG
725	Apigenin 5-O-glucoside	C21H20O10	M-H	-1.043845	431.09837	431.09792	18.174	NEG
726	Apigenin 7-glucoside	C21H20O10	M-H	-2.018101	431.09837	431.0975	19.645	NEG
727	Asperulosidic Acid	C18H24O12	M-H	-0.278345	431.1195	431.11938	11.188	NEG
728	Avicularin	C20H18O11	M-H	-1.385433	433.07764	433.07704	18.874	NEG
729	Isohemiphloin	C21H22O10	M-H	-1.824	433.11402	433.11323	16.801	NEG
730	Isoengelitin	C21H22O10	M-H	2.49357	433.11402	433.1151	20.377	NEG
731	Hemiphloin	C21H22O10	M-H	-0.046177	433.11402	433.114	16.215	NEG
732	Swertianolin	C20H20O11	M-H	-0.988294	435.09329	435.09286	20.107	NEG
733	7-O-Methylmangiferin	C20H20O11	M-H	1.815703	435.09329	435.09408	16.592	NEG
734	[6]-Gingerol	C17H26O4	M+H	-0.47427	295.19039	295.19025	26.843	POS
735	Corylifol A	C25H26O4	M+FA-H	1.631504	435.18131	435.18202	30.885	NEG

736	Rosin	C15H20O6	M+H	-8.682973	297.13326	297.13068	17.883	POS
737	Cryptotanshinone	C19H20O3	M+H	0.134613	297.14852	297.14856	31.364	POS
738	7-Methylguanosine	C11H16N5O5+	M+	-2.247458	298.1146	298.11393	1.09	POS
739	Stepharine	C18H19NO3	M+H	0.301868	298.14377	298.14386	13.073	POS
740	Lobetyolin	C20H28O8	M+FA-H	1.337333	441.17662	441.17721	19.811	NEG
741	Cinobufagin	C26H34O6	M-H	2.107753	441.22826	441.22919	28.055	NEG
742	Regaloside A	C18H24O10	M+FA-H	0.022465	445.13515	445.13516	14.108	NEG
743	Regaloside H	C18H24O10	M+FA-H	2.156648	445.13515	445.13611	13.018	NEG
744	Petroselinic acid	C18H34O2	M+NH4	-0.999035	300.2897	300.2894	33.591	POS
745	Isoorientin	C21H20O11	M-H	1.453835	447.09329	447.09394	15.724	NEG
746	Orientin	C21H20O11	M-H	1.297268	447.09329	447.09387	16.194	NEG
747	Isosakuranin	C22H24O10	M-H	-0.04473	447.12967	447.12965	21.417	NEG
748	Dihydrotamarixetin	C16H14O7	M+H-H2O	-3.587191	301.07125	301.07017	20.144	POS
749	Peonidin	C16H13O6+	M+	-0.730725	301.07066	301.07044	18.678	POS
750	3-epi-Padmatin	C16H14O7	M+H-H2O	1.062871	301.07125	301.07157	20.937	POS
751	Astilbin	C21H22O11	M-H	2.427028	449.10894	449.11003	18.112	NEG
752	Neoisoastilbin	C21H22O11	M-H	3.13955	449.10894	449.11035	18.83	NEG
753	Flavanomarein	C21H22O11	M-H	1.091049	449.10894	449.10943	13.959	NEG
754	Isofebrifugine	C16H19N3O3	M+H	2.349827	302.14992	302.15063	11.512	POS
755	Curculigoside B	C21H24O11	M-H	1.130508	451.12459	451.1251	19.02	NEG
756	2,3,5,4'-Tetrahydroxystilbene 2-O-	C20H22O9	M+FA-H	0.798006	451.12458	451.12494	17.482	NEG

	beta-D-glucoside							
757	Catechin 7-O-β-D-glucopyranoside	C21H24O11	M-H	1.573845	451.12459	451.1253	9.156	NEG
758	Robinetin	C15H10O7	M+H	0.065996	303.04993	303.04995	17.21	POS
759	6-Hydroxyluteolin	C15H10O7	M+H	1.616895	303.04993	303.05042	18.89	POS
760	Homoeriodictyol	C16H14O6	M+H	-1.682689	303.08631	303.0858	22.043	POS
761	Nigranoic acid	C30H46O4	M-H2O-H	-0.443143	451.32122	451.32102	35.624	NEG
762	Betulonic acid	C30H46O3	M-H	0.838228	453.33742	453.3378	35.059	NEG
763	Bruceine D	C20H26O9	M+FA-H	2.043256	455.15588	455.15681	12.101	NEG
764	Ursolic acid	C30H48O3	M-H	-1.405503	455.35307	455.35243	34.437	NEG
765	Betulinic acid	C30H48O3	M-H	-1.427464	455.35307	455.35242	34.223	NEG
766	Trametenolic acid	C30H48O3	M-H	-1.163932	455.35307	455.35254	35.476	NEG
767	Bigelovin	C17H20O5	M+H	0.426036	305.13835	305.13848	22.582	POS
768	Regaloside E	C20H26O12	M-H	-9.16578	457.13515	457.13096	16.53	NEG
769	Aleuritic acid	C16H32O5	M+H	-0.753525	305.23225	305.23202	20.917	POS
770	Anisodamine	C17H23NO4	M+H	-3.200836	306.16998	306.169	11.306	POS
771	Asperuloside	C18H22O11	M+FA-H	2.918662	459.11441	459.11575	12.851	NEG
772	Lucidenic acid N	C27H40O6	M-H	0.892711	459.27521	459.27562	22.268	NEG
773	Isorhamnetin 7-O-alpha-L-rhamnoside	C22H22O11	M-H	-0.889161	461.10894	461.10853	21.469	NEG
774	Toringin	C21H20O9	M+FA-H	-0.889161	461.10893	461.10852	20.818	NEG
775	Isoscoparin	C22H22O11	M-H	3.665077	461.10894	461.11063	18.238	NEG

776	Pratensein 7-O-glucopyranoside	C22H22O11	M-H	4.74942	461.10894	461.11113	19.707	NEG
777	Forsythoside E	C20H30O12	M-H	-5.160826	461.16645	461.16407	10.621	NEG
778	Manool	C20H34O	M+NH4	1.556951	308.29479	308.29527	35.308	POS
779	Isoquercitrin	C21H20O12	M-H	2.008257	463.0882	463.08913	17.61	NEG
780	Helichrysetin	C16H14O5	M+Na	1.391256	309.07334	309.07377	23.87	POS
781	Alantolactone	C15H20O2	2M-H	3.863709	463.28538	463.28717	30.823	NEG
782	Taxifolin 7-O-beta-D-glucoside	C21H22O12	M-H	0.688018	465.10385	465.10417	11.375	NEG
783	N-Acetylneuraminic acid	C11H19NO9	M+H	-0.290217	310.11326	310.11317	0.814	POS
784	Norswertianolin	C19H18O11	M+FA-H	-3.425515	467.08311	467.08151	16.089	NEG
785	Dehydroeburicoic acid	C31H48O3	M-H	-1.048458	467.35307	467.35258	35.698	NEG
786	Gypsogenin	C30H46O4	M-H	1.853697	469.33233	469.3332	32.048	NEG
787	Eburicoic acid	C31H50O3	M-H	-0.255663	469.36872	469.3686	35.976	NEG
788	Sibirioside A	C21H28O12	M-H	-0.169797	471.1508	471.15072	17.145	NEG
789	Alisol B	C30H48O4	M-H	2.84291	471.34798	471.34932	33.44	NEG
790	Hederagenin	C30H48O4	M-H	1.103219	471.34798	471.3485	31.219	NEG
791	Chicoric acid	C22H18O12	M-H	1.923595	473.07255	473.07346	16.235	NEG
792	Aflatoxin B1	C17H12O6	M+H	-0.255533	313.07066	313.07058	21.546	POS
793	Ombuin	C17H14O7	M+H-H2O	-1.373489	313.07125	313.07082	27.675	POS
794	Lotusine	C19H24NO3+	M+	1.464152	314.17507	314.17553	10.933	POS
795	Magnocurarine	C19H24NO3+	M+	1.336834	314.17507	314.17549	11.7	POS

796	Isorhamnetin-3-O-glucoside	C22H22O12	M-H	-1.00607	477.10385	477.10337	19.709	NEG
797	Nepitrin	C22H22O12	M-H	0.964151	477.10385	477.10431	19	NEG
798	Emodin-1-O-beta-D-glucopyranoside	C21H20O10	M+FA-H	-0.775513	477.10385	477.10348	20.168	NEG
799	5,7-Dimethoxyluteolin	C17H14O6	M+H	0.571272	315.08631	315.08649	21.169	POS
800	Salvianolic acid F	C17H14O6	M+H	0.698221	315.08631	315.08653	19.346	POS
801	p-Hydroxyphenethyl trans-ferulate	C18H18O5	M+H	-0.412538	315.1227	315.12257	24.749	POS
802	Prunin	C21H22O10	M+FA-H	-0.208716	479.1195	479.1194	19.187	NEG
803	Azaleatin	C16H12O7	M+H	0.599245	317.06558	317.06577	19.718	POS
804	Hamamelitannin	C20H20O14	M-H	0	483.07803	483.07803	11.747	NEG
805	Segetalin B	C24H32N6O5	M-H	3.000603	483.23614	483.23759	21.301	NEG
806	Dehydrotumulosic acid	C31H48O4	M-H	1.799945	483.34798	483.34885	31.671	NEG
807	Alisol C	C30H46O5	M-H	3.482187	485.32725	485.32894	27.613	NEG
808	Quillaic acid	C30H46O5	M-H	3.482187	485.32725	485.32894	30.131	NEG
809	Paeoniflorigenone	C17H18O6	M+H	-0.407373	319.11761	319.11748	21.877	POS
810	Isoagarotetrol	C17H18O6	M+H	-0.219355	319.11761	319.11754	16.859	POS
811	Andrograpanin	C20H30O3	M+H	-1.628936	319.22677	319.22625	28.812	POS
812	Rotundic acid	C30H48O5	M-H	2.729085	487.3429	487.34423	28.644	NEG
813	Arjunolic acid	C30H48O5	M-H	2.257138	487.3429	487.344	28.014	NEG
814	Dahurinol	C30H48O5	M-H	2.626487	487.3429	487.34418	30.49	NEG
815	Pseudocoptisine	C19H14NO4+	M+	-0.124964	320.09173	320.09169	19.903	POS

816	Cyclogalegenin	C30H50O5	M-H	3.126542	489.35855	489.36008	29.646	NEG
817	Iristectorin A	C23H24O12	M-H	1.445677	491.1195	491.12021	19.083	NEG
818	Oroxylin A-7-O-glucoside	C22H22O10	M+FA-H	1.588208	491.1195	491.12028	20.923	NEG
819	Protosappanin B	C16H16O6	M+NH4	1.117566	322.12851	322.12887	13.198	POS
820	Rehmannioside C	C21H34O14	M-H2O-H	0.447904	491.17646	491.17668	9.072	NEG
821	Methylnissolin	C17H16O5	M+Na	2.228488	323.08899	323.08971	23.912	POS
822	Salidroside	C14H20O7	M+Na	-0.278543	323.11012	323.11003	10.082	POS
823	5-O-Cinnamoylquinic acid	C16H18O7	M+H	-5.230376	323.11253	323.11084	15.941	POS
824	2-(3,4-dihydroxyphenyl)-7-(beta-D-glucopyranosyloxy)-8-hydroxy-4H-1-benzopyran-4-one	C21H20O11	M+FA-H	0.466438	493.09876	493.09899	17.189	NEG
825	(2R)-2-[1-[[6-O-(2-Carboxyacetyl)-beta-D-glucopyranosyl]oxy]-1-methylethyl]-2,3-dihydro-7H-furo[3,2-G][1]benzopyran-7-one	C23H26O12	M-H	2.413132	493.13515	493.13634	20.418	NEG
826	Harpagoside	C24H30O11	M-H	1.216615	493.17154	493.17214	20.946	NEG
827	Kauran-16,17-Diol	C20H34O2	M+NH4	-0.154183	324.2897	324.28965	32.985	POS
828	4,4'-Dihydroxy-	C17H18O5	M+Na	0.123037	325.10464	325.10468	22.355	POS

	2,6-dimethoxydihydrochalcone							
829	Toddalolactone	C16H20O6	M+NH4	0.03066	326.15981	326.15982	20.937	POS
830	Deoxylimonin	C26H30O7	M+FA-H	-0.200322	499.19735	499.19725	23.466	NEG
831	5α-Hydroxycostic acid	C15H22O3	2M-H	3.104306	499.30651	499.30806	28.329	NEG
832	Odoroside A	C30H46O7	M-H2O-H	4.025588	499.30596	499.30797	27.102	NEG
833	Boldine	C19H21NO4	M+H	-0.944677	328.15433	328.15402	13.406	POS
834	Cucurbitacin IIb	C30H48O7	M-H2O-H	3.929613	501.32161	501.32358	21.342	NEG
835	Medicagenic acid	C30H46O6	M-H	2.932246	501.32216	501.32363	29.251	NEG
836	Kaempferol-3,7,4'-trimethyl ether	C18H16O6	M+H	1.458515	329.10196	329.10244	31.192	POS
837	Salvigenin	C18H16O6	M+H	1.7016	329.10196	329.10252	29.382	POS
838	Gallocatechin gallate	C22H18O11	M+FA-H	-7.791953	503.08311	503.07919	14.653	NEG
839	Cinobufaginol	C26H34O7	M+FA-H	-8.823027	503.22865	503.22421	21.554	NEG
840	19alpha-Hydroxyasiatic acid	C30H48O6	M-H	3.437056	503.33781	503.33954	23.146	NEG
841	Iristectorigenin A	C17H14O7	M+H	-0.271837	331.08123	331.08114	22.355	POS
842	1-O-Galloyl-2-O-cinnamoyl-glucose	C22H22O11	M+FA-H	3.687531	507.11441	507.11628	20.46	NEG
843	Swertiajaponin	C22H22O11	M+FA-H	2.977632	507.11441	507.11592	16.571	NEG
844	Methylnissolin-3-O-glucoside	C23H26O10	M+FA-H	-1.47885	507.1508	507.15005	20.715	NEG
845	picroside IV	C24H28O12	M-H	0.39436	507.1508	507.151	15.799	NEG

846	Dihydrosinomenine	C19H25NO4	M+H	-0.421451	332.18563	332.18549	10.62	POS
847	7-[(beta-D-Glucopyranosyl)oxy]-3',4',5,8-tetrahydroxyflavone	C21H20O12	M+FA-H	0.392855	509.09368	509.09388	17.272	NEG
848	Isomucronulatol 7-O-glucoside	C23H28O10	M+FA-H	0.294599	509.16645	509.1666	20.63	NEG
849	Marrubiin	C20H28O4	M+H	-7.382819	333.20604	333.20358	29.131	POS
850	Poricoic acid a 3-methyl ester	C32H48O5	M-H	-1.290719	511.3429	511.34224	33.092	NEG
851	4',5,7-Trimethoxyflavone	C18H16O5	M+Na	0.805756	335.08899	335.08926	25.889	POS
852	Ganoderic acid DM	C30H44O4	M+FA-H	-1.168857	513.32216	513.32156	32.717	NEG
853	Epiberberine	C20H18NO4+	M+	-1.4578	336.12303	336.12254	19.993	POS
854	Isochlorogenic acid C	C25H24O12	M-H	0.892997	515.1195	515.11996	19.916	NEG
855	Umbelliferone 7-O-Rutinoside	C21H26O12	M+FA-H	-0.640602	515.14063	515.1403	11.56	NEG
856	Limonin	C26H30O8	M+FA-H	2.018664	515.19227	515.19331	25.037	NEG
857	16alpha-Hydroxydehydrotrametenolic acid	C30H46O4	M+FA-H	0.426904	515.33781	515.33803	30.969	NEG
858	Anisocoumarin H	C19H22O4	M+Na	-0.978819	337.14103	337.1407	28.139	POS
859	Neocryptotanshinone	C19H22O4	M+Na	-0.800852	337.14103	337.14076	30.089	POS
860	6'-O-beta-D-Glucosylgentiopicroside	C22H30O14	M-H	-3.38389	517.15628	517.15453	12.706	NEG
861	8-Epi-Loganic acid-	C22H34O15	M-H2O-H	0.231138	519.17138	519.1715	10.877	NEG

	6'-O-beta-D-glucoside							
862	Suspenoidside B	C25H30O12	M-H	0.805885	521.16645	521.16687	20.46	NEG
863	Loureirin B	C18H20O5	M+Na	5.455291	339.12029	339.12214	27.103	POS
864	Licochalcone C	C21H22O4	M+H	-0.235878	339.15909	339.15901	29.087	POS
865	4-Hydroxyderricin	C21H22O4	M+H	0.50124	339.15909	339.15926	32.619	POS
866	Crebanine	C20H21NO4	M+H	3.233826	340.15433	340.15543	20.876	POS
867	Daphnin	C15H16O9	M+H	1.671129	341.08671	341.08728	10.893	POS
868	Erianin	C18H22O5	M+Na	6.712866	341.13594	341.13823	26.843	POS
869	Monocrotaline N-Oxide	C16H23NO7	M+H	1.227515	342.15473	342.15515	9.706	POS
870	Phellodendrine	C20H24NO4+	M+	1.285911	342.16998	342.17042	12.908	POS
871	Silydianin	C25H22O10	M+FA-H	1.878132	527.1195	527.12049	20.566	NEG
872	Pachymic acid	C33H52O5	M-H	-0.170657	527.3742	527.37411	34.35	NEG
873	Eupatilin	C18H16O7	M+H	-1.21705	345.09688	345.09646	24.981	POS
874	Eupalinolide K	C20H26O6	M+H-H2O	-1.68033	345.17024	345.16966	20.853	POS
875	Dehydroandrographolide succinic acid	C28H36O10	M-H	1.807149	531.22357	531.22453	25.641	NEG
876	Phytolaccagenin	C31H48O7	M-H	0.809286	531.33273	531.33316	27.379	NEG
877	Alisol A 24-acetic acid	C32H52O6	M-H	-1.147978	531.36911	531.3685	31.903	NEG
878	Syringetin	C17H14O8	M+H	1.094861	347.07614	347.07652	22.499	POS
879	Quinine	C20H24N2O2	M+Na	-6.077662	347.173	347.17089	13.701	POS
880	Brevilin A	C20H26O5	M+H	-5.386173	347.1853	347.18343	28.01	POS
881	Picrocrocin	C16H26O7	M+NH4	-0.315909	348.20168	348.20157	14.447	POS

882	Picroside III	C25H30O13	M-H	-0.688806	537.16137	537.161	17.292	NEG
883	Ingenol	C20H28O5	M+H	-5.326446	349.20095	349.19909	22.312	POS
884	3-O-Caffeoylquinic acid methyl ester	C17H20O9	M+H-H2O	-8.34501	351.10803	351.1051	13.01	POS
885	Epoxylathyrol	C20H30O5	M+H	-6.60561	351.2166	351.21428	23.122	POS
886	7,8-Didehydro-2-hydroxy-3,7-dimethoxy-17-methylhasubanan-6-one	C19H23NO4	M+Na	-3.549604	352.15193	352.15068	10.724	POS
887	Semilicoisoflavone B	C20H16O6	M+H	0.254884	353.10196	353.10205	27.99	POS
888	Fargesin	C21H22O6	M+H-H2O	-3.341461	353.13894	353.13776	28.792	POS
889	Xanthohumol C	C21H20O5	M+H	0.311493	353.13835	353.13846	32.117	POS
890	Chrysin 6-C-arabinoside 8-C-glucoside	C26H28O13	M-H	-0.16449	547.14571	547.14562	17.398	NEG
891	Chrysin 6-C-glucoside 8-C-arabinoside	C26H28O13	M-H	-0.584853	547.14571	547.14539	18.259	NEG
892	Bruceantin	C28H36O11	M-H	-1.059906	547.21849	547.21791	25.459	NEG
893	Polygalaxanthone III	C25H28O15	M-H2O-H	-2.221719	549.12443	549.12321	17.062	NEG
894	Gentianose	C18H32O16	M+FA-H	1.602426	549.16723	549.16811	0.869	NEG
895	Genipin 1-beta-D-gentiobioside	C23H34O15	M-H	-0.746564	549.18249	549.18208	11.956	NEG
896	Chlorogenic acid	C16H18O9	M+H	-1.041953	355.10236	355.10199	11.492	POS
897	Marmin	C19H24O5	M+Na	-2.90017	355.15159	355.15056	23.223	POS

898	(2S)-Isoxanthohumol	C21H22O5	M+H	-2.308858	355.154	355.15318	24.212	POS
899	Isoxanthohumol	C21H22O5	M+H	0.309725	355.154	355.15411	24.625	POS
900	Aviculin	C26H34O10	M+FA-H	3.356232	551.2134	551.21525	17.922	NEG
901	Dinoprost	C20H34O5	M+H	-6.361755	355.2479	355.24564	22.729	POS
902	Rotundine	C21H25NO4	M+H	1.010709	356.18563	356.18599	20.185	POS
903	Kirenol	C20H34O4	M+NH4	0.196475	356.27953	356.2796	21.233	POS
904	Chrysoobtusin	C19H18O7	M+H	1.141703	359.11253	359.11294	27.36	POS
905	5-Desmethylsinensetin	C19H18O7	M+H	1.225243	359.11253	359.11297	28.139	POS
906	Gardenin B	C19H18O7	M+H	1.475861	359.11253	359.11306	30.261	POS
907	Kushenol O	C27H30O13	M-H	0.106921	561.16137	561.16143	20	NEG
908	Vicenin-1	C26H28O14	M-H	-1.456119	563.14063	563.13981	15.423	NEG
909	Isoschaftoside	C26H28O14	M-H	-1.260786	563.14063	563.13992	16.53	NEG
910	Schaftoside	C26H28O14	M-H	-1.243029	563.14063	563.13993	15.695	NEG
911	3,5-Dimethoxy-1-[(6-O-beta-D-xylopyranosyl-beta-D-glucopyranosyl)oxy]-9H-xanthen-9-one	C26H30O14	M-H	2.441802	565.15628	565.15766	20.44	NEG
912	Pinoresinol 4-O-beta-D-glucopyranoside	C26H32O11	M+FA-H	2.193942	565.19266	565.1939	19.105	NEG
913	6-Methoxytricin	C18H16O8	M+H	1.412383	361.09179	361.0923	22.52	POS

914	Triptolide	C20H24O6	M+H	-5.676083	361.16456	361.16251	21.253	POS
915	Secoisolariciresinol monoglucoside	C26H36O11	M+FA-H	3.232471	569.22396	569.2258	18.388	NEG
916	Danmelittoside	C15H22O10	M+H	-7.793383	363.12857	363.12574	6.921	POS
917	Deacetylorientalide	C19H22O7	M+H	1.459477	363.14383	363.14436	17.948	POS
918	Methylophiopogonanone A	C19H18O6	M+Na	8.600394	365.09956	365.1027	30.628	POS
919	β-Gentiobiose	C12H22O11	M+Na	-1.287299	365.10543	365.10496	0.889	POS
920	Coniferin	C16H22O8	M+Na	1.971951	365.12069	365.12141	10.995	POS
921	Oridonin	C20H28O6	M+H	-3.751412	365.19586	365.19449	21.169	POS
922	Procyanidin B4	C30H26O12	M-H	-1.299522	577.13515	577.1344	12.268	NEG
923	Chrysin 7-O-beta-gentiobioside	C27H30O14	M-H	-5.717689	577.15628	577.15298	19.939	NEG
924	Isoviolanthin	C27H30O14	M-H	0.84899	577.15628	577.15677	17.356	NEG
925	Tetrahydrocortisone	C21H32O5	M+H	-0.191659	365.23225	365.23218	22.126	POS
926	Naringin	C27H32O14	M-H	0.08633	579.17193	579.17198	19.328	NEG
927	Episyringaresinol 4'-O-beta-D-glncopyranoside	C28H36O13	M-H	8.356234	579.20832	579.21316	19.328	NEG
928	Spiculisporic acid	C17H28O6	M+K	5.283919	367.15175	367.15369	29.417	POS
929	Bicuculline	C20H17NO6	M+H	-0.760636	368.11286	368.11258	15.836	POS
930	Usaramine N-oxide	C18H25NO7	M+H	1.195099	368.17038	368.17082	12.35	POS
931	4-Feruloylquinic acid	C17H20O9	M+H	-1.273306	369.11801	369.11754	14.676	POS

932	Curcumin	C21H20O6	M+H	0.460538	369.13326	369.13343	28.587	POS
933	Jasminoside B	C16H26O8	M+Na	-0.975208	369.15199	369.15163	10.954	POS
934	Vitexin 4'-glucoside	C27H30O15	M-H	-0.876674	593.15119	593.15067	16.821	NEG
935	Lonicerin	C27H30O15	M-H	-0.134873	593.15119	593.15111	18.094	NEG
936	Puerarin 6"-O-Xyloside	C26H28O13	M+FA-H	-0.084296	593.15119	593.15114	14.257	NEG
937	Allocryptopine	C21H23NO5	M+H	0.75642	370.1649	370.16518	19.364	POS
938	Didymin	C28H34O14	M-H	3.321041	593.18758	593.18955	20.672	NEG
939	4'-Methoxyagarotetrol	C18H20O7	M+Na	2.074856	371.11012	371.11089	16.381	POS
940	Artemitin	C20H20O8	M+H-H2O	-1.643704	371.11312	371.11251	28.792	POS
941	Cassiaside C	C27H32O15	M-H	0.386446	595.16684	595.16707	20.756	NEG
942	3,7,4'-Trihydroxy-5-methoxy-8-prenylflavanone	C21H22O6	M+H	1.724375	371.14891	371.14955	23.495	POS
943	Kouitchenside G	C27H32O15	M-H	3.394006	595.16684	595.16886	19.56	NEG
944	Tracheloside	C27H34O12	M+FA-H	0.68884	595.20323	595.20364	20.481	NEG
945	N-p-coumaroyl-Octopamine	C17H17NO4	2M-H	-6.195328	597.22424	597.22054	17.104	NEG
946	Pentamethylquercetin	C20H20O7	M+H	1.956432	373.12818	373.12891	25.128	POS
947	Isosinensetin	C20H20O7	M+H	1.983233	373.12818	373.12892	23.349	POS
948	Cis-Ferulic acid 4-O-beta-D-glucopyranoside	C16H20O9	M+NH4	0.721646	374.14456	374.14483	12.804	POS
949	Zeatinriboside	C15H21N5O5	M+Na	3.447875	374.14349	374.14478	11.41	POS

950	Aristolochic acid D	C17H11NO8	M+NH4	2.772725	375.08229	375.08333	22.956	POS
951	Neotheaflavin	C29H24O12	M+FA-H	0.246255	609.12498	609.12513	20.128	NEG
952	Kaempferol 3-O-gentiobioside	C27H30O16	M-H	-0.410411	609.14611	609.14586	17.441	NEG
953	Diffractaic acid	C20H22O7	M+H	-0.186595	375.14383	375.14376	31.427	POS
954	6'-Hydroxy-3,4,2',3',4'-pentamethoxychalcone	C20H22O7	M+H	0.159939	375.14383	375.14389	30.416	POS
955	Hexahydrocurcumin	C21H26O6	M+H	1.0395	375.18021	375.1806	21.587	POS
956	Quercetin 3-o-(6"-galloyl)-beta-d-glucopyranoside	C28H24O16	M-H	2.454889	615.09916	615.10067	16.78	NEG
957	Daucosterol	C35H60O6	M+FA-H	2.848236	621.43719	621.43896	37.871	NEG
958	Puerarin-4'-O-beta-D-glucopyranoside	C27H30O14	M+FA-H	3.626667	623.16175	623.16401	10.682	NEG
959	6”-O-xylosyl-glycitin	C27H30O14	M+FA-H	3.546431	623.16175	623.16396	15.298	NEG
960	Verbascoside	C29H36O15	M-H	-0.946729	623.19814	623.19755	18.174	NEG
961	Zeylenone	C21H18O7	M+H	-1.357304	383.11253	383.11201	26.864	POS
962	Hydrastine	C21H21NO6	M+H	0.520638	384.14416	384.14436	16.735	POS
963	Macrozamin	C13H24N2O11	M+H	4.595668	385.14529	385.14706	5.928	POS
964	Calenduloside E	C36H56O9	M-H	3.183477	631.38516	631.38717	29.919	NEG
965	Echinocystic acid 28-O-β-D-glucoside	C36H58O9	M-H	2.273442	633.40081	633.40225	28.894	NEG
966	Moluccanin	C20H18O8	M+H	1.57579	387.10744	387.10805	21.149	POS

967	(20R)-Ginsenoside Rh1	C36H62O9	M-H	2.619887	637.43211	637.43378	23.126	NEG
968	Oroxin B	C27H30O15	M+FA-H	-4.427709	639.15667	639.15384	19.539	NEG
969	Isorhamnetin 3,7-O-diglucoside	C28H32O17	M-H	2.080867	639.15667	639.158	12.851	NEG
970	Isolugrandoside	C29H36O16	M-H	-1.095131	639.19306	639.19236	15.633	NEG
971	Ethyl rosmarinate	C20H20O8	M+H	-0.745265	389.12309	389.1228	22.332	POS
972	Benzoyloxypaeoniflorin	C30H32O13	M+FA-H	3.983369	645.18249	645.18506	20.63	NEG
973	Neodiosmin	C28H32O15	M+FA-H	1.974977	653.17232	653.17361	20.023	NEG
974	Syringetin-3-O-rutinoside	C29H34O17	M-H	2.189315	653.17232	653.17375	19.288	NEG
975	Sinensetin	C20H20O7	M+Na	-1.594492	395.11012	395.10949	24.772	POS
976	Syringin	C17H24O9	M+Na	-1.493175	395.13125	395.13066	11.911	POS
977	6',7'-Dihydroxybergamottin	C21H24O6	M+Na	2.454786	395.14651	395.14748	25.274	POS
978	Deguelin	C23H22O6	M+H	-4.732393	395.14891	395.14704	29.981	POS
979	Cimicifugoside H-1	C35H52O9	M+FA-H	0.861861	661.35933	661.3599	24.889	NEG
980	Ilexsaponin A	C36H56O11	M-H	4.401734	663.37499	663.37791	24.995	NEG
981	Secologanic acid	C16H22O10	M+Na	-1.787915	397.11051	397.1098	11.785	POS
982	Picropodophyllin	C22H22O8	M+H-H2O	-2.795063	397.12877	397.12766	22.764	POS
983	Isoacteoside	C29H36O15	M+FA-H	1.00119	669.20362	669.20429	19.308	NEG
984	Isoforsythiaside	C29H36O15	M+FA-H	0.881645	669.20362	669.20421	17.082	NEG
985	Leocarpinolide F	C20H24O7	M+Na	1.904087	399.14142	399.14218	20.312	POS
986	Angelol A	C20H24O7	M+Na	2.6557	399.14142	399.14248	23.101	POS

987	4'-Demethylepipodophyllotoxin	C21H20O8	M+H	0.64818	401.12309	401.12335	21.253	POS
988	3-Methoxytangeretin	C21H22O8	M+H	0.198443	403.13874	403.13882	29.234	POS
989	Pedunculoside	C36H58O10	M+FA-H	1.020993	695.4012	695.40191	22.303	NEG
990	Albaspidin AA	C21H24O8	M+H	2.887788	405.15439	405.15556	34.737	POS
991	Trachelogenin	C21H24O7	M+NH4	-0.073858	406.18603	406.186	21.837	POS
992	26-Deoxycimicifugoside	C37H54O10	M+FA-H	5.018696	703.3699	703.37343	26.541	NEG
993	Calycanthoside	C17H20O10	M+Na	-1.031701	407.09486	407.09444	12.784	POS
994	Nystose	C24H42O21	M+FA-H	0.224966	711.22006	711.22022	2.449	NEG
995	Maltotetraose	C24H42O21	M+FA-H	0.112483	711.22006	711.22014	0.844	NEG
996	Eudesmin	C22H26O6	M+Na	-0.391043	409.16216	409.162	26.374	POS
997	1,4-beta-Xylopentaose	C25H42O21	M+FA-H	2.654794	723.22006	723.22198	2.365	NEG
998	Camelliaside B	C32H38O19	M-H	2.482096	725.19345	725.19525	16.957	NEG
999	Matairesinol 4'-O-beta-gentiobioside	C32H42O16	M+FA-H	5.321449	727.24548	727.24935	19.245	NEG
1000	Pinoresinol Diglucoside	C32H42O16	M+FA-H	1.60881	727.24548	727.24665	15.318	NEG
1001	4''-methyloxy-Daidzin	C22H22O9	M+H-H2O	-6.462956	413.12368	413.12101	17.36	POS
1002	Manghaslin	C33H40O20	M-H2O-H	2.18396	737.1929	737.19451	15.633	NEG
1003	Clitorin	C33H40O19	M-H	1.366325	739.2091	739.21011	16.738	NEG
1004	Troxerutin	C33H42O19	M-H	-0.350771	741.22475	741.22449	18.915	NEG
1005	Kaempferol 3-alpha-L-	C20H18O10	M+H	3.054184	419.09727	419.09855	19.264	POS

	arabinopyranoside							
1006	Complanatoside B	C33H40O20	M-H	1.99946	755.20402	755.20553	19.477	NEG
1007	Kaempferol 3-sophoroside-7-rhamnoside	C33H40O20	M-H	0.794487	755.20402	755.20462	14.384	NEG
1008	Kuwanon A	C25H24O6	M+H	1.163441	421.16456	421.16505	31.213	POS
1009	Morusin	C25H24O6	M+H	1.115953	421.16456	421.16503	31.796	POS
1010	Atractyloside	C30H46O16S2	M+FA-H	-7.598342	771.22093	771.21507	21.113	NEG
1011	Parishin C	C32H40O19	M+FA-H	0.775981	773.21458	773.21518	15.234	NEG
1012	Artesunate	C19H28O8	M+K	0.827146	423.14158	423.14193	28.204	POS
1013	Betulinaldehyde	C30H48O2	M+H-H2O	-2.787208	423.36273	423.36155	36.55	POS
1014	Leptophylloside	C20H24O10	M+H	-2.211014	425.14422	425.14328	16.9	POS
1015	Isorhamnetin 3-sophoroside-7-rhamnoside	C34H42O21	M-H	2.801782	785.21458	785.21678	14.53	NEG
1016	Betulin	C30H50O2	M+H-H2O	-1.528051	425.37838	425.37773	34.655	POS
1017	Gardenoside	C17H24O11	M+Na	0.070238	427.12108	427.12111	9.417	POS
1018	Desoxyrhaponticin	C21H24O8	M+Na	2.505055	427.13634	427.13741	20.563	POS
1019	Chikusetsusaponin Iva	C42H66O14	M-H	0.655376	793.43798	793.4385	24.639	NEG
1020	Tenacissoside H	C42H66O14	M-H	0.655376	793.43798	793.4385	30.087	NEG
1021	Alcesefoliside	C33H40O20	M+FA-H	1.148264	801.20949	801.21041	15.466	NEG
1022	Picfeltarraenin IA	C41H62O13	M+FA-H	2.216945	807.41724	807.41903	25.81	NEG
1023	Esculentoside A	C42H66O16	M-H2O-H	2.638043	807.4167	807.41883	22.521	NEG
1024	Nodakenin	C20H24O9	M+Na	-1.554051	431.13125	431.13058	18.471	POS

1025	Regaloside C	C18H24O11	M+NH4	0.276392	434.16568	434.1658	12.722	POS
1026	Isocoreopsin	C21H22O10	M+H	-0.459634	435.12857	435.12837	14.655	POS
1027	Pseudolaric Acid C2	C22H26O8	M+NH4	1.948663	436.19659	436.19744	21.61	POS
1028	Perisesaccharide B	C36H60O18	M+FA-H	-8.178089	825.37616	825.36941	27.401	NEG
1029	Isokurarinone	C26H30O6	M+H	0.956259	439.21151	439.21193	31.192	POS
1030	Kurarinone	C26H30O6	M+H	1.798678	439.21151	439.2123	27.905	POS
1031	Phlorigidoside C	C17H24O11	M+K	1.647502	443.09502	443.09575	9.706	POS
1032	Hispidulin 4'-O-beta-D-glucopyranoside	C22H22O11	M+H-H2O	-7.795764	445.11351	445.11004	19.82	POS
1033	Neolinustatin	C17H29NO11	M+Na	-0.20172	446.16328	446.16319	9.15	POS
1034	Baicalin	C21H18O11	M+H	2.102475	447.09219	447.09313	20.5	POS
1035	Glychionide A	C21H18O11	M+H	1.744607	447.09219	447.09297	20.833	POS
1036	Prunetrin	C22H22O10	M+H	0.424934	447.12857	447.12876	21.302	POS
1037	Calycosin-7-O-beta-D-glucoside	C22H22O10	M+H	0.402569	447.12857	447.12875	17.046	POS
1038	Quercitrin	C21H20O11	M+H	-0.667991	449.10784	449.10754	19.428	POS
1039	β-D-Glucopyranosyl abscisate	C21H30O9	M+Na	1.536139	449.1782	449.17889	17.654	POS
1040	Pennogenin 3-O-beta-chacotrioside	C45H72O17	M-H2O-H	1.409657	865.45856	865.45978	27.592	NEG
1041	Dioscin	C45H72O16	M-H	5.037611	867.47476	867.47913	29.87	NEG
1042	Isoastragaloside I	C45H72O16	M-H	4.841639	867.47476	867.47896	29.393	NEG
1043	Taxifolin 7-O-rhamnoside	C21H22O11	M+H	-0.399004	451.12349	451.12331	15.546	POS

1044	Neosmitilbin	C21H22O11	M+H	-2.039353	451.12349	451.12257	17.507	POS
1045	Schisandrin	C24H32O7	M+Na	0.219682	455.20402	455.20412	27.696	POS
1046	Saikogenin F	C30H48O4	M+H-H2O	0.680791	455.35256	455.35287	29.172	POS
1047	Pomolic acid	C30H48O4	M+H-H2O	0.505103	455.35256	455.35279	31.448	POS
1048	Ganodermanontriol	C30H48O4	M+H-H2O	0.505103	455.35256	455.35279	32.117	POS
1049	Cinobufotalin	C26H34O7	M+H	1.219412	459.23773	459.23829	23.558	POS
1050	Acetylarenobufagin	C26H34O7	M+H	1.350063	459.23773	459.23835	26.927	POS
1051	Baicalin methyl ester	C22H20O11	M+H	2.710863	461.10784	461.10909	21.276	POS
1052	Sec-O-Glucosylhamaudol	C21H26O10	M+Na	4.640655	461.14182	461.14396	20.583	POS
1053	Andrographidine C	C23H24O10	M+H	-1.192685	461.14422	461.14367	21.567	POS
1054	Picfeltarraenin IV	C47H72O18	M-H	-0.7147	923.46459	923.46393	25.184	NEG
1055	Dehydrosoyasaponin I	C48H76O18	M-H	0.606708	939.49589	939.49646	27.592	NEG
1056	7,2′-Dihydroxy-3′,4′-dimethoxyisoflavan 7-O-β-D-glucoside	C23H28O10	M+H	-3.568545	465.17552	465.17386	20.396	POS
1057	alpha-Cyclodextrin	C36H60O30	M-H2O-H	3.482645	953.29855	953.30187	6.225	NEG
1058	Kudinoside D	C47H72O17	M+FA-H	0.062928	953.47515	953.47521	23.001	NEG
1059	Ginsenoside Ro	C48H76O19	M-H	-9.241324	955.4908	955.48197	22.836	NEG
1060	Gaultherin	C19H26O12	M+Na	-0.170528	469.13164	469.13156	14.365	POS
1061	Tormentic acid	C30H48O5	M+H-H2O	-2.015498	471.34747	471.34652	29.023	POS
1062	Saikogenin D	C30H48O4	M+H	-2.55618	473.36254	473.36133	28.833	POS

1063	Neoamygdalin	C20H27NO11	M+NH4	3.514367	475.19223	475.1939	12.079	POS
1064	1,1,1,1-Kestohexaose	C36H62O31	M+FA-H	1.168714	1035.3257	1035.32691	5.844	NEG
1065	Fabiatrin	C21H26O13	M+H	0.266861	487.14462	487.14475	12.246	POS
1066	Arjungenin	C30H48O6	M+H-H2O	-2.421296	487.34239	487.34121	23.828	POS
1067	Arjunic acid	C30H48O5	M+H	1.185228	489.35745	489.35803	29.214	POS
1068	Aurantio-obtusin beta-D-glucoside	C23H24O12	M+H	0.547518	493.13405	493.13432	20.228	POS
1069	Swertiaside	C23H28O12	M+H	1.327526	497.16535	497.16601	19.49	POS
1070	Adynerin	C30H44O7	M+H-H2O	1.141585	499.306	499.30657	27.99	POS
1071	Daidzin 6″-O-malonate	C24H22O12	M+H	-1.550331	503.1184	503.11762	18.678	POS
1072	Iridin	C24H26O13	M+H-H2O	1.247192	505.13464	505.13527	19.614	POS
1073	Olean-12-ene-3beta,16beta,21beta,23,28-pentol	C30H50O5	M+Na	3.993338	513.35504	513.35709	23.684	POS
1074	Angeloylgomisin H	C28H36O8	M+Na	0.477801	523.23024	523.23049	30.197	POS
1075	Terminolic acid	C30H48O6	M+Na	1.915294	527.33431	527.33532	26.163	POS
1076	Gymnemagenin	C30H50O6	M+Na	6.38519	529.34996	529.35334	21.979	POS
1077	4,5-Di-O-caffeoylquinic acid methyl ester	C26H26O12	M+H	0.357715	531.1497	531.14989	20.5	POS
1078	Kukoamine B	C28H42N4O6	M+H	-0.432886	531.31771	531.31748	10.347	POS
1079	Schisantherin C	C28H34O9	M+NH4	2.949719	532.25411	532.25568	31.042	POS
1080	Picroside II	C23H28O13	M+Na	6.203958	535.14221	535.14553	17.507	POS
1081	Matairesinoside	C26H32O11	M+NH4	2.545388	538.22828	538.22965	20.124	POS

1082	Epipinoresinol-4-O-beta-D-glucoside	C26H32O11	M+NH4	2.954137	538.22828	538.22987	19.614	POS
1083	Maltotriose	C18H32O16	M+K	0.699646	543.13219	543.13257	0.872	POS
1084	Gomisin D	C28H34O10	M+NH4	1.64159	548.24902	548.24992	28.435	POS
1085	Arctiin	C27H34O11	M+NH4	2.788623	552.24393	552.24547	20.813	POS
1086	Gomisin G	C30H32O9	M+Na	1.430631	559.19385	559.19465	30.633	POS
1087	Oleuropein	C25H32O13	M+Na	7.368955	563.17351	563.17766	20.333	POS
1088	Cassiaside B	C26H30O14	M+H	-0.969725	567.17083	567.17028	21.065	POS
1089	6'-Feruloylnodakenin	C30H32O12	M+H	3.36639	585.19665	585.19862	21.021	POS
1090	Sibiricose A1	C23H32O15	M+K	-0.66424	587.13728	587.13689	14.573	POS
1091	Amarogentin	C29H30O13	M+H	0.289521	587.17592	587.17609	20.917	POS
1092	Linarin	C28H32O14	M+H	3.219898	593.18648	593.18839	20.396	POS
1093	Biorobin	C27H30O15	M+H	3.730053	595.16575	595.16797	18.199	POS
1094	Saponarin	C27H30O15	M+H	3.847668	595.16575	595.16804	15.193	POS
1095	Cannabisin A	C34H30N2O8	M+H	-6.098714	595.20749	595.20386	20.144	POS
1096	Eriocitrin	C27H32O15	M+H	-0.870757	597.1814	597.18088	16.859	POS
1097	Prosaikogenin F	C36H58O8	M+H-H2O	0.814751	601.41047	601.41096	27.82	POS
1098	Acanthoside B	C28H36O13	M+Na	-0.165781	603.20481	603.20471	19.592	POS
1099	Isoliensinine	C37H42N2O6	M+H	0.997855	611.31156	611.31217	16.066	POS
1100	ecliptasaponin D	C36H58O9	M+H-H2O	1.927421	617.40538	617.40657	27.145	POS
1101	6-hydroxyapigenin-6-O-beta-D-glucoside-	C27H28O17	M+H	-2.14352	625.13993	625.13859	15.129	POS

	7-O-beta-D-glucuronide							
1102	Brassidin	C28H32O16	M+H	1.663531	625.17631	625.17735	17.551	POS
1103	Dauricine	C38H44N2O6	M+H	1.998953	625.32721	625.32846	17.739	POS
1104	Pseudoginsenoside RT5	C36H62O10	M+H-H2O	0.92559	637.4316	637.43219	22.083	POS
1105	Picfeltarraenin X	C36H54O11	M+H	0.572829	663.37389	663.37427	22.126	POS
1106	Anemarrhena saponin A2	C39H64O14	M+Na	5.965984	779.41882	779.42347	27.421	POS
1107	Damulin B	C42H70O13	M+Na	6.952455	805.47086	805.47646	29.403	POS
1108	1F-Fructofuranosylnystose	C30H52O26	M+H-H2O	0.036979	811.27198	811.27201	5.765	POS
1109	Dulcoside A	C38H60O17	M+Na	-2.501934	811.37227	811.37024	21.712	POS
1110	Gypenoside L	C42H72O14	M+Na	4.687416	823.48142	823.48528	28.01	POS
1111	Maltohexaose	C36H62O31	M+H	-0.161399	991.33478	991.33462	1.877	POS
1112	Fructo-oligosaccharide DP7/GF6	C42H72O36	M+H-H2O	1.153801	1135.37763	1135.37894	5.805	POS
1113	1,3-Benzenediol	C6H6O2	M-H	-9.17183	109.0295	109.0285	6.967	NEG
1114	1-Methyl-2-pyrrolecarboxaldehyde	C6H7NO	M+H	4.724694	110.06004	110.06056	12.824	POS
1115	2-Aminopyridine	C5H6N2	M+NH4	5.263772	112.08692	112.08751	1.16	POS
1116	4-HYDROXYISONICOTINIC ACID	C6H5NO3	M-H	-7.245344	138.01967	138.01867	1.892	NEG
1117	1,2,4-Trihydroxybenzene	C6H6O3	M+H	3.14864	127.03897	127.03937	0.815	POS
1118	glutinic acid	C5H4O4	M+H	2.635287	129.01823	129.01857	1.641	POS

1119	DL-Glutamate	C5H9NO4	M+H-H2O	-1.614758	130.05046	130.05025	0.711	POS
1120	DL-Pyroglutamic acid	C5H7NO3	M+H	2.460595	130.04987	130.05019	1.944	POS
1121	Agmatine	C5H14N4	M+H	1.525214	131.12912	131.12932	0.693	POS
1122	4-Hydroxy-2-methoxybenzaldehyde	C8H8O3	M-H	-6.686967	151.04007	151.03906	14.716	NEG
1123	DL-Asparagine	C4H8N2O3	M+H	0.601229	133.06077	133.06085	0.714	POS
1124	D-Histidine	C6H9N3O2	M-H	-5.971614	154.0622	154.06128	0.674	NEG
1125	Parasorbic acid	C6H8O2	M+FA-H	-5.921657	157.05063	157.0497	11.747	NEG
1126	Octopamine	C8H11NO2	M+H-H2O	-2.572087	136.07628	136.07593	2.487	POS
1127	(1R,2R,3S)-3-methylcyclopentane-1,2-diol	C6H12O2	M+FA-H	-6.208021	161.08193	161.08093	8.503	NEG
1128	2,3-Dihydroxybenzaldehyde	C7H6O3	M+H	1.510368	139.03897	139.03918	10.393	POS
1129	threono-1,4-lactone	C4H6O4	M+FA-H	-5.704653	163.02481	163.02388	0.844	NEG
1130	2-Cyclooctenone	C8H12O	M+FA-H	-4.199022	169.08701	169.0863	22.353	NEG
1131	6-(hydroxymethyl)pyridin-3-ol	C6H7NO2	M+FA-H	-5.763151	170.04588	170.0449	2.134	NEG
1132	threo-5-n-pentyl-4-hydroxy tetrahydrofuran-2-one	C9H16O3	M-H	-4.032666	171.10267	171.10198	22.1	NEG
1133	2-Deoxy-L-ribono-1,4-lactone	C5H8O4	M+FA-H	-5.196552	177.04046	177.03954	1.822	NEG

1134	3,4-Dimethoxybenzamide	C9H11NO3	M-H	-3.55424	180.06662	180.06598	15.674	NEG
1135	p-hydroxy-coumaric acid	C9H10O4	M-H	-3.921555	181.05063	181.04992	7.515	NEG
1136	dl-Mesatone	C9H13NO2	M+H-H2O	-2.465156	150.09193	150.09156	6.05	POS
1137	4,4-dimethyl-1,7-heptanedioicacid	C9H16O4	M-H	-2.72585	187.09758	187.09707	16.278	NEG
1138	menisdaurin_qt	C8H9NO2	M+H	0.986384	152.0706	152.07075	6.07	POS
1139	(-)-Norephedrine	C9H13NO	M+H	0.460202	152.10699	152.10706	3.502	POS
1140	N-Acetyl-DL-glutamic acid	C7H11NO5	M-H	-3.296882	188.05645	188.05583	2.283	NEG
1141	Butoxysuccinic Acid	C8H14O5	M-H	-3.64931	189.07685	189.07616	13.938	NEG
1142	Verbenol	C10H16O	M+H	2.089763	153.12739	153.12771	20.542	POS
1143	p-Mentha-1,8-dien-4-ol	C10H16O	M+H	0.391831	153.12739	153.12745	23.223	POS
1144	Amphetamine	C9H13N	M+NH4	1.828409	153.13862	153.1389	9.767	POS
1145	4-hydroxy-2-β-D-glucopyranosyl oxypheylacetonitrile_qt	C8H7NO2	M+FA-H	-2.93745	194.04588	194.04531	10.024	NEG
1146	Damascenine	C10H13NO3	M-H	-3.091472	194.08227	194.08167	21.113	NEG
1147	Benzo-1,3-dioxole-5-methylamine	C8H9NO2	M+FA-H	-2.601224	196.06153	196.06102	10.409	NEG
1148	L-2-Amino-3-(1-pyrazolyl)propanoic acid	C6H9N3O2	M+H	0.576639	156.07675	156.07684	2.528	POS
1149	SCOPOLINE	C8H13NO2	M+H	2.498368	156.1019	156.10229	4.286	POS

1150	3-Hydroxybenzylhydrazine	C7H10N2O	M+NH4	1.47329	156.11314	156.11337	6.09	POS
1151	(±)-Camphoric acid	C10H16O4	M-H	-2.662011	199.09758	199.09705	20.586	NEG
1152	2-nonenic acid	C9H16O2	M+H	1.654762	157.12231	157.12257	24.834	POS
1153	6,7-dihydroxy-3,7-dimethyloct-2-enoic acid	C10H18O4	M-H	-2.585608	201.11323	201.11271	14.633	NEG
1154	cis-linalool,oxide	C9H16O2	M+FA-H	-2.883948	201.11323	201.11265	22.815	NEG
1155	6-keto-n-caprylic acid	C8H14O3	M+FA-H	-2.511171	203.09249	203.09198	18.979	NEG
1156	rengyoxide	C8H14O3	M+FA-H	-2.90508	203.09249	203.0919	12.351	NEG
1157	L-2-Amino-4-methylenepentanedioic acid	C6H9NO4	M+FA-H	-3.332494	204.05136	204.05068	3.163	NEG
1158	Allixin	C12H18O4	M-H2O-H	0.241427	207.10212	207.10217	24.32	NEG
1159	N-(3,4,5-trimethoxyphenyl)formamide	C10H13NO4	M-H	-2.76089	210.07718	210.0766	13.729	NEG
1160	Gentiatibetine	C9H11NO2	M+H	0.903145	166.08625	166.0864	2.507	POS
1161	Myrtenic acid	C10H14O2	M+FA-H	-1.94223	211.09758	211.09717	20.044	NEG
1162	Racephedrine	C10H15NO	M+H	1.565109	166.12264	166.1229	4.584	POS
1163	10-hydroxyverbenone	C10H14O2	M+H	1.855103	167.10666	167.10697	14.489	POS
1164	1,2-Cyclohexanediol, 1-methyl-4-(1-methylethenyl)-	C10H18O2	M+FA-H	-1.859351	215.12888	215.12848	21.994	NEG

1165	griffonin_qt	C8H9NO3	M+H	1.487515	168.06552	168.06577	2.304	POS
1166	5-(2-hydroxyethyl)-2-methoxyphenol	C9H12O3	M+H	1.892529	169.08592	169.08624	13.721	POS
1167	allomatatabiol	C10H16O2	M+H	0.886932	169.12231	169.12246	15.297	POS
1168	saikochromic acid	C10H6O6	M-H	-2.941055	221.00916	221.00851	12.873	NEG
1169	4-o-acetyl-caffeic acid	C11H10O5	M-H	-2.850091	221.04555	221.04492	15.109	NEG
1170	Pemoline	C9H8N2O2	M+FA-H	-0.090474	221.05678	221.05676	8.058	NEG
1171	5-(3,4-Methylenedioxyphenyl)pentanoic acid	C12H14O4	M-H	-1.628356	221.08193	221.08157	22.123	NEG
1172	(4S,5S)-4-hydroxy-5-pentyloxolan-2-one	C9H16O3	M+H	2.252809	173.11722	173.11761	25.086	POS
1173	Peperinic acid	C10H14O3	M+FA-H	-1.012803	227.09249	227.09226	17.755	NEG
1174	Bornyl formate	C11H18O2	M+FA-H	0.044028	227.12888	227.12889	24.211	NEG
1175	dimethyl camphorate	C12H20O4	M-H	0.088056	227.12888	227.1289	22.661	NEG
1176	schizonodiol	C10H16O3	M+FA-H	-0.69836	229.10814	229.10798	15.17	NEG
1177	DL-Arginine	C6H14N4O2	M+H	0.057104	175.11895	175.11896	0.7	POS
1178	Gentianine	C10H9NO2	M+H	1.022317	176.0706	176.07078	14.174	POS
1179	Calystegine B2	C7H13NO4	M+H	1.9876	176.09173	176.09208	0.748	POS
1180	cis-p-menth-2-ene-1α,7,8-triol	C10H18O3	M+FA-H	-0.951871	231.12379	231.12357	20.23	NEG
1181	beta-hydrojuglone	C10H8O3	M+H	0.395358	177.05462	177.05469	14.676	POS
1182	regaloside D_qt	C12H14O5	M-H	-2.02466	237.07685	237.07637	15.002	NEG

1183	(S)-Isosclerone	C10H10O3	M+H	0.893504	179.07027	179.07043	12.865	POS
1184	(E-p-hydroxy-cinnamic acid	C10H10O3	M+H	0.502596	179.07027	179.07036	14.833	POS
1185	(2-amylphenyl)methanol	C12H18O	M+H	0.669856	179.14304	179.14316	31.796	POS
1186	methyl 4-[2-formyl-5-(methoxymethyl)pyrrol-1-yl]butanoate	C12H17NO4	M-H	0.293984	238.10848	238.10855	20.651	NEG
1187	6-methyl-7-(3-oxobutyl)-bicyclo[4.1.0]heptan-3-one	C12H18O2	M+FA-H	-0.878188	239.12888	239.12867	23.908	NEG
1188	Ganoine	C11H17NO2	M+FA-H	-0.374806	240.12413	240.12404	20.189	NEG
1189	Jasmolone	C11H16O2	M+H	1.711551	181.12231	181.12262	20.568	POS
1190	1-O-Galloylglycerol	C10H12O7	M-H	-1.604601	243.05103	243.05064	6.31	NEG
1191	1-[(2R,3R,4S,5S)-3,4-dihydroxy-5-(hydroxymethyl)oxolan-2-yl]pyrimidine-2,4-dione	C9H12N2O6	M-H	-1.892519	243.06226	243.0618	2.595	NEG
1192	Mimosine	C8H10N2O4	M+FA-H	-0.205709	243.06226	243.06221	1.465	NEG
1193	Tridecan-2-one	C13H26O	M+FA-H	1.439165	243.19656	243.19691	32.136	NEG
1194	Genipic acid	C9H12O4	M+H	2.971675	185.08083	185.08138	11.954	POS
1195	(4r)-4-hydroxymethylboschnialactone	C10H16O3	M+H	2.755011	185.11722	185.11773	19.162	POS
1196	Hyaluronic acid_qt	C8H15NO5	M+FA-H	-1.119583	250.09322	250.09294	0.728	NEG

1197	trans-p-menth-2-ene-1α,7,8-triol	C10H18O3	M+H	1.175635	187.13287	187.13309	12.329	POS
1198	(z)-6,7-epoxy-6,7-dihydroligustilide	C12H14O3	M+FA-H	0.19913	251.09249	251.09254	24.32	NEG
1199	Propoxur	C11H15NO3	M+FA-H	1.338038	254.10339	254.10373	23.76	NEG
1200	Cinchoninic acid, 2-hydroxy-	C10H7NO3	M+H	-0.157853	190.04987	190.04984	10.353	POS
1201	(-)-Cytisine	C11H14N2O	M+H	0.366266	191.11789	191.11796	7.273	POS
1202	n-demethyldoryphornine	C10H9NO3	M+H	2.134688	192.06552	192.06593	9.746	POS
1203	trans-2-phenylcyclopropane-1-carboxylic acid	C11H13NO2	M+H	0.312334	192.1019	192.10196	10.539	POS
1204	daphnetin-8-methyl ether	C10H8O4	M+H	0.932403	193.04953	193.04971	13.742	POS
1205	S-(2-Carboxyethyl)-L-cysteine	C6H11NO4S	M+H	-0.257668	194.04816	194.04811	0.909	POS
1206	L-1,2,3,4-Tetrahydro-beta-carboline-3-carboxylic acid	C12H12N2O2	M+FA-H	-0.536218	261.08808	261.08794	9.341	NEG
1207	Austricin	C15H18O4	M-H	-1.493605	261.11323	261.11284	22.836	NEG
1208	(1S,2S)-1,2-bis(2-furyl)ethane-1,2-diol	C10H10O4	M+H	3.02463	195.06518	195.06577	11.159	POS
1209	Zizyphus saponin III	C10H10O4	M+H	2.768305	195.06518	195.06572	12.35	POS
1210	6-[(2S)-2,3-dihydroxy-3-methyl-	C14H16O5	M-H	-0.380094	263.0925	263.0924	22.079	NEG

	butyl]-7-hydroxy-coumarin							
1211	khusilic acid	C14H18O2	M+FA-H	0.646071	263.12888	263.12905	28.685	NEG
1212	Curcolonol	C15H20O4	M-H	0.418046	263.12888	263.12899	22.48	NEG
1213	wilforonide	C13H16O3	M+FA-H	1.772861	265.10814	265.10861	21.806	NEG
1214	1-Propanone, 3-hydroxy-1-(4-hydroxy-3-methoxyphenyl)-	C10H12O4	M+H	1.369996	197.08083	197.0811	12.366	POS
1215	rhizonic acid	C10H12O4	M+H	1.877402	197.08083	197.0812	20.208	POS
1216	Paeonilactone B	C10H12O4	M+H	0.761109	197.08083	197.08098	14.264	POS
1217	4α,5α-epoxy-6α-hydroxy amorphan-12-oicacid	C15H24O4	M-H	0.935768	267.16018	267.16043	21.301	NEG
1218	isololiolide	C11H16O3	M+H	1.116087	197.11722	197.11744	17.108	POS
1219	3,4,2',3 '-tetrahydroxychalcone	C15H12O5	M-H	-1.217437	271.0612	271.06087	16.875	NEG
1220	(3R,4aS,5R,6R)-6-hydroxy-3-methoxy-5-vinyl-4,4a,5,6-tetrahydro-3H-pyrano[5,4-c]pyran-1-one	C11H14O5	M+FA-H	-9.185402	271.08232	271.07983	15.799	NEG
1221	carbalexin c	C14H13NO2	M+FA-H	-0.51453	272.09283	272.09269	20.903	NEG
1222	japonicumin D	C13H24O3	M+FA-H	0.183036	273.17074	273.17079	21.008	NEG
1223	11-hydroxy-9-	C13H24O3	M+FA-H	0.585714	273.17074	273.1709	29.168	NEG

	tridecenoicacid							
1224	(1R,3R)-1-methyl-2,3,4,9-tetrahydro-1H-$b-carboline-3-carboxylic acid	C13H14N2O2	M+FA-H	0.872398	275.10373	275.10397	12.268	NEG
1225	Paniculide B	C15H20O5	M-H	1.003139	279.1238	279.12408	17.694	NEG
1226	triacanthine	C10H13N5	M+H	-5.63382	204.12437	204.12322	12.659	POS
1227	Methyl 14-methyl-8-hexadecenoate	C18H36O2	M-H	0.105908	283.26425	283.26428	36.6	NEG
1228	balata	C12H15NO2	M+H	0.630708	206.11755	206.11768	8.964	POS
1229	4,2',5'-trihydroxy-4'-methoxychalcone	C16H14O5	M-H	1.683757	285.07685	285.07733	21.847	NEG
1230	apocynol a	C13H20O3	M+H-H2O	-0.868984	207.13854	207.13836	21.317	POS
1231	norannuic acid	C13H20O3	M+H-H2O	-1.158645	207.13854	207.1383	20.981	POS
1232	4-Ethoxy-4-oxobut-2-enoic acid	C6H8O4	2M-H	1.149516	287.07724	287.07757	8.271	NEG
1233	6-(4-hydroxyphenyl)-4-hydroxyhexan-2-one	C12H16O3	M+H	0.956401	209.11722	209.11742	19.943	POS
1234	4-[(E)-3-ethoxyprop-1-enyl]-2-methoxyphenol	C12H16O3	M+H	1.004221	209.11722	209.11743	24.191	POS
1235	2-(phenylmethylene)heptanol	C16H22O2	M+FA-H	0.034345	291.16018	291.16019	30.949	NEG

1236	Sedanonic acid	C12H18O3	M+H	0.66309	211.13287	211.13301	24.688	POS
1237	1,5-di-isobutyl-3,3-dimethyl[3,1,0]-cyclohexadione	C16H26O2	M+FA-H	0.271011	295.19148	295.19156	29.438	NEG
1238	Melevodopa	C10H13NO4	M+H	1.414482	212.09173	212.09203	6.881	POS
1239	(6S)-6-[(1R,4R,5R)-4,5-dihydroxy-4-methyl-1-cyclohex-2-enyl]-2-methylhept-2-en-4-one	C15H24O3	M+FA-H	-1.783486	297.17074	297.17021	23.718	NEG
1240	geniposidie acid_qt	C10H12O5	M+H	0.84477	213.07575	213.07593	8.902	POS
1241	cucurbic acid	C12H20O3	M+H	1.032144	213.14852	213.14874	25.976	POS
1242	3-isopropyl-5-acetoxycyclohexene-2-one-1	C11H16O3	M+NH4	2.848554	214.14377	214.14438	20.897	POS
1243	Phenylglucoside	C12H16O6	M+FA-H	0.697459	301.09289	301.0931	10.729	NEG
1244	cadinanetriol	C15H28O3	M+FA-H	-1.626815	301.20204	301.20155	23.146	NEG
1245	teuclatriol	C15H28O3	M+FA-H	-0.531205	301.20204	301.20188	21.994	NEG
1246	2-(2-hydroxy-4-methylphenyl)propane-1,2,3-triol	C10H14O4	M+NH4	1.156749	216.12303	216.12328	8.697	POS
1247	Norathyriol	C13H8O6	M+FA-H	-7.441884	305.03029	305.02802	16.696	NEG
1248	3-Hexenyl-beta-	C12H22O6	M+FA-H	0.716286	307.13984	307.14006	15.423	NEG

	glucopyranoside							
1249	4β-methoxycostuslactone	C16H22O3	M+FA-H	-0.455796	307.15509	307.15495	28.435	NEG
1250	cyclic peptide	C10H10N6O6	M-H	1.261895	309.05891	309.0593	10.176	NEG
1251	4-methoxy-5-hydroxybisabola-2,10-diene-9-one	C16H26O3	M+FA-H	-0.12854	311.18639	311.18635	26.856	NEG
1252	Bungeiside B	C14H18O8	M-H	0.54297	313.09289	313.09306	9.217	NEG
1253	Perilloside A	C16H26O6	M-H	-8.238451	313.16566	313.16308	18.218	NEG
1254	Benzoic acid + 2O, O-Hex	C13H16O9	M-H	1.333028	315.07216	315.07258	7.515	NEG
1255	Hexyl salicylic acid	C13H18O3	M+H	1.478939	223.13287	223.1332	29.045	POS
1256	radicamine b	C11H15NO4	M+H	0.442268	226.10738	226.10748	1.636	POS
1257	2-methylidene-4-[(2R,3R,4S,5S,6R)-3,4,5-trihydroxy-6-(hydroxymethyl)oxan-2-yl]oxybutanoic acid	C11H18O8	M+FA-H	-0.495205	323.09837	323.09821	7.2	NEG
1258	methyl-(3s,5s)-5-hydroxy-3-(β-d-glucopyra-nosyloxy)hexanoate	C13H24O9	M-H	-1.547342	323.13476	323.13426	7.827	NEG
1259	FA 12:3+2O	C12H18O4	M+H	1.540983	227.12779	227.12814	20.52	POS
1260	Coumaroyl Hexoside (isomer of 691, 692)	C15H18O8	M-H	-2.09171	325.09289	325.09221	11.497	NEG

1261	3α,7α-dihydroxy amorph-4-ene 3-acetate	C17H28O3	M+FA-H	-0.215251	325.20204	325.20197	30.426	NEG
1262	5'-o-methyladenosine	C11H15N5O4	M+FA-H	0.27598	326.1106	326.11069	7.494	NEG
1263	Dihydromelilotoside	C15H20O8	M-H	1.222836	327.10854	327.10894	10.687	NEG
1264	Paeonoside	C15H20O8	M-H	-0.764272	327.10854	327.10829	14.816	NEG
1265	4-[1-(4-Hydroxyphenyl)penta-1,4-dien-3-yl]-2-methoxyphenol	C18H18O3	M+FA-H	-1.100501	327.12379	327.12343	27.654	NEG
1266	(12Z,15Z)-9,10,11-Trihydroxy-12,15-Octadecadienoic acid	C18H32O5	M-H	-0.641775	327.2177	327.21749	23.198	NEG
1267	Auxin a	C18H32O5	M-H	0.213925	327.2177	327.21777	27.952	NEG
1268	melazolide a	C11H16O4	M+NH4	1.47737	230.13868	230.13902	16.358	POS
1269	woodorien	C14H18O9	M-H	2.005544	329.08781	329.08847	13.269	NEG
1270	beta-Glucogallin	C13H16O10	M-H	-0.120821	331.06707	331.06703	5.087	NEG
1271	gallic acid 3-O-β-D-glucoside	C13H16O10	M-H	-0.151027	331.06707	331.06702	5.613	NEG
1272	6-Galloylglucose	C13H16O10	M-H	-0.060411	331.06707	331.06705	2.824	NEG
1273	gamma-l-glutamyl-l-beta-aminoisibutyric acid	C9H16N2O5	M+H	1.286928	233.1132	233.1135	2.181	POS

1274	2,2'bioxazolidine-3,3'-diethanol	C10H20N2O4	M+H	-0.600473	233.14958	233.14944	2.304	POS
1275	3-O-Caffeoylshikimic acid	C16H16O8	M-H	0.089532	335.07724	335.07727	14.91	NEG
1276	(1alpha,4beta,5beta)-4-Hydroxy-7(11),10(14)-guaiadien-8-one	C15H22O2	M+H	1.020542	235.16926	235.1695	22.605	POS
1277	cnidimol B	C15H16O6	M+FA-H	2.046914	337.09289	337.09358	16.215	NEG
1278	cotarnine	C12H15NO4	M+H	-0.461976	238.10738	238.10727	9.213	POS
1279	(S)-Tetrahydrocolumbamine	C20H23NO4	M-H	0.264585	340.15543	340.15552	13.812	NEG
1280	Glucosyringic acid	C15H20O10	M-H2O-H	0.850222	341.08726	341.08755	11.106	NEG
1281	3-phenyl-2-propenol-O-β-D-glucopyranoside	C15H20O6	M+FA-H	-0.439723	341.12419	341.12404	15.045	NEG
1282	oxyphyllenodiol a	C14H22O3	M+H	0.961683	239.16417	239.1644	22.271	POS
1283	Theogallin	C14H16O10	M-H	-0.408083	343.06707	343.06693	2.511	NEG
1284	Rivularin (flavone)	C18H16O7	M-H	0.728688	343.08233	343.08258	25.959	NEG
1285	PCG	C10H12N5O7P	M-H	0.465062	344.04016	344.04032	4.317	NEG
1286	Methyl 6-O-galloyl-beta-D-glucopyranoside	C14H18O10	M-H	-0.60855	345.08272	345.08251	8.822	NEG
1287	picrocrocinicacido-β-d-glucopyrinoside	C16H26O8	M-H	-0.724311	345.15549	345.15524	16.404	NEG

1288	villoside	C16H26O8	M-H	-0.608421	345.15549	345.15528	16.978	NEG
1289	3-[[(2S)-2,4-Dihydroxy-3,3-dimethylbutanoyl]amino]propanoic acid	C9H17NO5	M+Na	0.660884	242.09989	242.10005	6.753	POS
1290	deacetyl asperuloside acid_qt	C11H14O6	M+H	0.658202	243.08631	243.08647	7.91	POS
1291	3,4-Dihydroxybenzyl alcohol-4-glucoside	C13H18O8	M+FA-H	-0.547395	347.09837	347.09818	6.626	NEG
1292	columbianetin propionate	C17H18O5	M+FA-H	-6.424409	347.11362	347.11139	25.522	NEG
1293	3-n-butyl-3-hydroxy-4,5,6,7-tetrahydro-6,7-dihydroxyphthalide	C12H18O5	M+H	0.781498	243.1227	243.12289	13.887	POS
1294	3,5,7,2'6'-pentahydroxyflavanone	C15H12O7	M+FA-H	-6.388651	349.0565	349.05427	14.341	NEG
1295	9-ribosyl-trans-zeatin	C15H21N5O5	M-H	-3.112978	350.14699	350.1459	11.23	NEG
1296	1-[(2R,3S,4S,5S)-3,4-dihydroxy-5-(hydroxymethyl)oxolan-2-yl]pyrimidine-2,4-dione	C9H12N2O6	M+H	0.571249	245.07681	245.07695	2.813	POS
1297	Licoisoflavone B	C20H16O6	M-H	0.199381	351.08741	351.08748	30.531	NEG
1298	γ-glutamyl-valine	C10H18N2O5	M+H	-0.283253	247.12885	247.12878	1.736	POS
1299	przewaquinone f	C18H16O5	M+FA-H	-1.316165	357.09797	357.0975	23.167	NEG
1300	miltionone i	C19H20O4	M+FA-H	-5.684135	357.13436	357.13233	31.053	NEG

1301	(10s)-15-methoxypinusolidicacid	C21H30O5	M-H	-5.952347	361.20205	361.1999	32.136	NEG
1302	tuberostemospironine	C13H19NO4	M+H	0.236092	254.13868	254.13874	7.89	POS
1303	4-hydroxy-4-[2-[(2R,3R,4S,5S,6R)-3,4,5-trihydroxy-6-(hydroxymethyl)oxan-2-yl]oxyethyl]cyclohexan-1-one	C14H24O8	M+FA-H	0.492954	365.14532	365.1455	10.219	NEG
1304	1-o-caffeoylglycerol	C12H14O6	M+H	2.58736	255.08631	255.08697	11.159	POS
1305	Poncitrin	C20H22O4	M+FA-H	1.104675	371.15001	371.15042	29.983	NEG
1306	methylrosmarinate	C19H18O8	M-H	0.375242	373.09289	373.09303	18.174	NEG
1307	Capillarol	C15H16O4	M+H	0.919145	261.11213	261.11237	28.498	POS
1308	(S)-Auraptenol	C15H16O4	M+H	1.034039	261.11213	261.1124	20.624	POS
1309	homovanillyl alcohol-4-o-glucoside	C15H22O8	M+FA-H	1.759392	375.12967	375.13033	14.021	NEG
1310	16-hydroxytriptolide	C20H24O7	M-H	-0.106625	375.14493	375.14489	17.292	NEG
1311	lamiol	C16H26O10	M-H	0.636359	377.14532	377.14556	12.331	NEG
1312	chinensiolide a	C15H18O4	M+H	0.760087	263.12779	263.12799	20.396	POS
1313	shizukanolide f	C15H18O4	M+H	0.570065	263.12779	263.12794	20.853	POS
1314	(1S,2S,4R)-1,8-Epoxy-p-menthan-2-ol glucoside	C16H28O7	M+FA-H	0.583273	377.1817	377.18192	20.251	NEG
1315	hautriwaic acid	C20H28O4	M+FA-H	-6.548303	377.19696	377.19449	27.908	NEG

1316	mamanine	C15H22N2O2	M+H	1.557896	263.1754	263.17581	12.162	POS
1317	divaricataester,a	C14H16O5	M+H	1.735148	265.10705	265.10751	16.504	POS
1318	hypodematine	C17H13NO	M+NH4	0.528036	265.13354	265.13368	24.276	POS
1319	Des-O-methyllasiodiplodin	C15H20O4	M+H	0.678878	265.14344	265.14362	23.849	POS
1320	Glyceollin I	C20H18O5	M+FA-H	2.088153	383.11362	383.11442	23.084	NEG
1321	magnolone	C21H22O7	M-H	2.388808	385.12928	385.1302	22.773	NEG
1322	(?)-caaverine	C17H17NO2	M+H	1.044257	268.1332	268.13348	16.148	POS
1323	codonopyrrolidium b	C14H22NO4+	M+	0.335628	268.15433	268.15442	7.461	POS
1324	lithospermidin A	C21H24O7	M-H	-0.749073	387.14493	387.14464	27.908	NEG
1325	12:4+3O fatty acyl hexoside	C18H28O9	M-H	1.291436	387.16606	387.16656	13.479	NEG
1326	9'-o-methylamericanol a	C19H20O6	M+FA-H	2.621271	389.12419	389.12521	17.694	NEG
1327	Fagaramide	C14H17NO3	M+Na	6.034577	270.11006	270.11169	22.226	POS
1328	3,4-Dihydroverbenalin	C17H26O10	M-H	-0.334065	389.14532	389.14519	10.661	NEG
1329	(2S,3S,4S,5R)-2,3,4,5,6-pentahydroxyhexanoic acid	C6H12O7	2M-H	-1.252847	391.10933	391.10884	0.747	NEG
1330	pedicularis-lactone-1-o-beta-d-glucoside	C15H22O9	M+FA-H	-6.979873	391.12458	391.12185	11.601	NEG
1331	3-(2-hydroxy-4-methoxyphenyl)-2H-chromen-7-ol	C16H14O4	M+H	1.991911	271.09648	271.09702	21.169	POS
1332	Jasminoside	C16H26O8	M+FA-H	0.076695	391.16097	391.161	11.477	NEG

1333	chrysanthemumin D	C15H26O4	M+H	-0.958736	271.19039	271.19013	17.759	POS
1334	Aposiopolamine	C16H17NO3	M+H	0	272.12812	272.12812	19.863	POS
1335	(e)-2-hexenyl-α-l-arabinopyranosyl-(1→2)-β-d-glucopyranoside	C17H30O10	M-H	0.381508	393.17662	393.17677	12.768	NEG
1336	Microminutin	C15H12O5	M+H	0.805637	273.07575	273.07597	25.555	POS
1337	1β,4β,6α,15-tetrahydroxyeudesmane	C15H28O4	M+H	-0.109807	273.20604	273.20601	19.057	POS
1338	(3s,6r)-6,7-dihydroxy-6,7-dihydrolinalool-3-o-β-d-glucopyranoside	C16H30O8	M+FA-H	-0.455474	395.19227	395.19209	12.123	NEG
1339	norpluviine	C16H19NO3	M+H	0.875453	274.14377	274.14401	9.52	POS
1340	polyacetylene PQ-1	C18H28O3	M+H-H2O	-3.015976	275.20114	275.20031	27.885	POS
1341	6β-hydroxyhuperzine a	C15H18N2O2	M+NH4	0.615561	276.17065	276.17082	7.992	POS
1342	(2S,3R,4S,5S,6R)-2-[4-(2-hydroxyethyl)-2,6-dimethoxy-phenoxy]-6-methylol-tetrahydropyran-3,4,5-triol	C16H24O9	M+FA-H	-5.010611	405.14023	405.1382	9.507	NEG
1343	alpha-Eleostearic acid	C18H30O2	M+H	0.752063	279.23186	279.23207	31.427	POS
1344	khellol glucoside	C19H20O10	M-H	0.221077	407.09837	407.09846	14.426	NEG

1345	1-o-β-d-glucopyranosylamplexin	C16H26O9	M+FA-H	0.515773	407.15588	407.15609	11.353	NEG
1346	glyinflanin A	C25H28O5	M-H	1.596321	407.1864	407.18705	32.906	NEG
1347	Dehydrojuncusol	C18H16O2	M+NH4	1.842999	282.14885	282.14937	27.166	POS
1348	sonchuside a	C21H32O8	M-H	-2.042789	411.20244	411.2016	20.545	NEG
1349	qinghaosu iv	C15H22O5	M+H	2.613419	283.154	283.15474	20.853	POS
1350	celephtalide a	C18H24O8	M+FA-H	-0.79875	413.14532	413.14499	17.609	NEG
1351	7-hydroxy-2-[4-[(2S,3R,4S,5S,6R)-3,4,5-trihydroxy-6-methylol-tetrahydropyran-2-yl]oxyphenyl]chromone	C21H20O9	M-H	0.337265	415.10346	415.1036	16.111	NEG
1352	Eleganin	C22H26O9	M-H2O-H	1.469386	415.13929	415.1399	22.71	NEG
1353	Rubrofusarin	C15H16N4O2	M+H	-3.787685	285.1346	285.13352	15.4	POS
1354	Tangshenoside II	C17H24O9	M+FA-H	0.863019	417.14023	417.14059	13.667	NEG
1355	(2,2')-Binaphthalene-1,1',4,4'-tetrone, 8,8'-dihydroxy-6,6'-dimethyl-	C22H14O6	M+FA-H	-4.390599	419.07724	419.0754	30.172	NEG
1356	isozeylanone	C22H14O6	M+FA-H	-4.676942	419.07724	419.07528	28.014	NEG
1357	Neodiospyrin	C22H14O6	M+FA-H	0.763582	419.07724	419.07756	25.768	NEG
1358	Dihydrosyringin	C17H26O9	M+FA-H	0.310147	419.15588	419.15601	12.395	NEG
1359	7,8-Dimethyl-10-[(2R,3R,4S)-	C17H20N4O6	M+FA-H	1.495952	421.13648	421.13711	13.98	NEG

	2,3,4,5-tetrahydroxypentyl]benzo[g]pteridine-2,4-dione							
1360	pluviine	C17H21NO3	M+H	-0.173515	288.15942	288.15937	10.496	POS
1361	(2S)-3',5,5',7-tetrahydroxyflavanone	C15H12O6	M+H	1.522119	289.07066	289.0711	17.507	POS
1362	2-methyl-3-[(2S,3R,4S,5S,6R)-3,4,5-trihydroxy-6-(hydroxymethyl)oxan-2-yl]oxypyran-4-one	C12H16O8	M+H	-0.691822	289.09179	289.09159	7.583	POS
1363	alginic acid	C14H22O12	M+FA-H	-2.411561	427.10933	427.1083	2.283	NEG
1364	15,16-bisnor-13-oxo-8(17),11e-labdadien-19-oicacid	C18H26O3	M+H	0.377753	291.19547	291.19558	26.291	POS
1365	Homonataloin	C22H24O9	M-H	-2.342655	431.13476	431.13375	14.848	NEG
1366	majoroside	C17H24O10	M+FA-H	-0.300137	433.13515	433.13502	11.064	NEG
1367	Rehmaionoside C	C19H32O8	M+FA-H	0.923344	433.20792	433.20832	13.626	NEG
1368	cis-Piceid	C20H22O8	M+FA-H	-1.332936	435.12967	435.12909	15.149	NEG
1369	Semiglabrin	C23H20O6	M+FA-H	-5.719198	437.12419	437.12169	28.56	NEG
1370	bis(4-hydroxybenzyl)ether mono-beta-d-glucopyranoside	C20H24O8	M+FA-H	-0.137254	437.14532	437.14526	14.405	NEG
1371	Mulberrofuran B	C25H28O4	M+FA-H	0.480333	437.19696	437.19717	33.205	NEG

1372	(+)-purpurin 2	C23H22O6	M+FA-H	-6.148383	439.13984	439.13714	29.983	NEG
1373	Psilostachyin	C15H20O5	M+NH4	-0.402462	298.1649	298.16478	21.61	POS
1374	(2S,3S)-pterosin C 3-O- β-glucopyranoside	C20H28O8	M+FA-H	-0.748	441.17662	441.17629	16.895	NEG
1375	acetylbullatantriol	C17H30O4	M+H	0.768661	299.22169	299.22192	29.553	POS
1376	Kaempferol 5-methyl ether	C16H12O6	M+H	0.166074	301.07066	301.07071	25.107	POS
1377	benzyl alcoholβ-d-(2'-o-β-xylopyranosyl)glucopyranoside	C18H26O10	M+FA-H	-0.581459	447.1508	447.15054	12.893	NEG
1378	3'-o-methyl brazilin	C17H16O5	M+H	-3.022181	301.10705	301.10614	14.01	POS
1379	Coelogin	C17H16O5	M+H	-0.464951	301.10705	301.10691	21.877	POS
1380	4',5,7-trihydroxy-6-methyl-homoisoflavanone	C17H16O5	M+H	-0.43174	301.10705	301.10692	26.101	POS
1381	Neocarthamin	C21H22O11	M-H	0.578924	449.10894	449.1092	15.527	NEG
1382	Prupersin	C22H26O10	M-H	1.781161	449.14532	449.14612	14.857	NEG
1383	Alysifolinone	C16H14O6	M+H	-0.857841	303.08631	303.08605	20.144	POS
1384	sappanone b	C16H14O6	M+H	-0.857841	303.08631	303.08605	17.779	POS
1385	marioside	C22H34O10	M-H	-0.524925	457.20792	457.20768	18.894	NEG
1386	Pinnatifinoside A	C21H18O9	M+FA-H	2.134642	459.09328	459.09426	19.126	NEG
1387	anticancer glycerol ester	C22H22O8	M+FA-H	-0.980115	459.12967	459.12922	20.44	NEG

	pmv70p691-118							
1388	Glutamyl histidine	C11H16N4O5	M+Na	1.563002	307.10129	307.10177	4.11	POS
1389	2-Phenylethyl beta-D-glucopyranoside	C14H20O6	M+Na	-0.032561	307.11521	307.1152	15.4	POS
1390	Sayaendoside	C19H28O10	M+FA-H	0.195157	461.16645	461.16654	13.895	NEG
1391	3'-o-acetylloganic acid	C18H26O11	M+FA-H	-0.690927	463.14571	463.14539	9.321	NEG
1392	Dihydrocumambrin A	C17H24O5	M+H	-7.730384	309.16965	309.16726	20.479	POS
1393	Silymonin	C25H22O9	M-H	1.805989	465.11911	465.11995	20.023	NEG
1394	Tripteroside	C19H18O11	M+FA-H	1.648529	467.08311	467.08388	13.626	NEG
1395	chloranoside a	C21H28O9	M+FA-H	-4.326776	469.17153	469.1695	16.069	NEG
1396	cassipourol	C20H38O	M+NH4	2.305283	312.32609	312.32681	36.118	POS
1397	(2s)-5,7-dimethoxy-8-formylflavanone	C18H16O5	M+H	0.255504	313.10705	313.10713	31.172	POS
1398	Scutellarein 4'-methyl ether 7-glucuronide	C22H20O12	M-H	2.841578	475.0882	475.08955	16.801	NEG
1399	hispidulin-7-o-glucuronide	C22H20O12	M-H	0.757754	475.0882	475.08856	19.707	NEG
1400	5,4'-dihydroxyl-6,7-dimethoxyl-8-c-[β-d-xylo-copyranosyl-(1→2)]-β-d-glucopyranosylflavone	C10H17ClFN3O5	M+H	1.655569	314.09135	314.09187	5.724	POS
1401	isocephalotaxinone	C18H19NO4	M+H	-0.063666	314.13868	314.13866	12.639	POS

1402	p-Hydroxy-cinnamic acid	C9H16N4O6	M+K	3.459547	315.07014	315.07123	4.854	POS
1403	Muningin	C17H14O6	M+H	0.507797	315.08631	315.08647	25.065	POS
1404	4-hydroxyphenyl-β-gentiobioside	C18H26O12	M+FA-H	0.29219	479.14063	479.14077	7.87	NEG
1405	11-methyl-6-methylidene-16-oxo-15-oxapentacyclo[9.3.2.15,8.01,10.02,8]heptadec-13-ene-9-carboxylic acid	C19H22O4	M+H	1.68169	315.15909	315.15962	21.526	POS
1406	1-(2'-γ-pyranone)-6-caffeoyl-α-d-pyranoglucose	C20H20O11	M+FA-H	1.288717	481.09876	481.09938	13.458	NEG
1407	Gallic acid 4-O-(6-galloylglucoside)	C20H20O14	M-H	0.103503	483.07803	483.07808	8.037	NEG
1408	Scullcapflavone	C17H16O6	M+H	0.977604	317.10196	317.10227	29.981	POS
1409	Gallic acid 3-O-(6-galloylglucoside)	C20H20O14	M-H	-0.041401	483.07803	483.07801	12.643	NEG
1410	3,5-dimethoxy-4-hydroxyphenol1-o-β-d-(6'-o-galloyl)glucopyranoside	C21H24O13	M-H	0.745165	483.11441	483.11477	15.066	NEG
1411	cangoronine	C30H44O5	M-H	1.820772	483.3116	483.31248	31.715	NEG
1412	Pongamoside A	C23H20O9	M+FA-H	-6.534615	485.10893	485.10576	20.46	NEG

1413	Pfaffic acid	C29H44O3	M+FA-H	0.721163	485.32724	485.32759	32.592	NEG
1414	4-hydroxysphinganine	C18H39NO3	M+H	0.094251	318.30027	318.3003	28.553	POS
1415	Dihydroisorhamnetin	C16H14O7	M+H	-0.75216	319.08123	319.08099	18.134	POS
1416	1,6-dihydroxy-3,5,7-trimethoxyxanthone	C16H14O7	M+H	-0.03134	319.08123	319.08122	26.8	POS
1417	(3R,4S)-3-(4-hydroxy-3-methoxy-benzyl)chroman-3,4,7-triol	C17H18O6	M+H	-0.125346	319.11761	319.11757	14.093	POS
1418	nilgirine	C17H23NO5	M+H	0.1552	322.1649	322.16495	20.102	POS
1419	Bungeiside D	C19H26O12	M+FA-H	0.346133	491.14063	491.1408	11.167	NEG
1420	2-O-alpha-D-Galactopyranosyl-1-deoxynojirimycin	C12H23NO9	M+H	-0.153306	326.14456	326.14451	0.732	POS
1421	N,N'-Dinitro-1,2-Cyclohexanediamine	C20H18N6O10	M-H	-0.498901	501.10116	501.10091	16.78	NEG
1422	cynanchol	C18H16O6	M+H	-0.243086	329.10196	329.10188	22.21	POS
1423	1-[3-methoxy-4-[(2R,3R,4S,5S,6R)-3,4,5-trihydroxy-6-(hydroxymethyl)oxan-2-yl]oxyphenyl]ethanone	C15H20O8	M+H	0.638059	329.12309	329.1233	13.992	POS
1424	rhododendrin	C16H24O7	M+H	1.45826	329.15948	329.15996	15.982	POS

1425	beta-D-Galactopyranosyl-(1->4)-beta-D-galactopyranosyl-(1->4)-D-galactose	C18H32O16	M-H	-0.834722	503.16176	503.16134	2.449	NEG
1426	Panose	C18H32O16	M-H	-1.33158	503.16176	503.16109	2.785	NEG
1427	Loniceracetalide A	C21H32O11	M+FA-H	1.821087	505.19266	505.19358	20.398	NEG
1428	2',3'-dihydroxy-1'-propoxypseudolarate b	C26H34O10	M-H	1.642888	505.20792	505.20875	21.595	NEG
1429	phenylpropanoids	C17H14O7	M+H	0.030204	331.08123	331.08124	17.841	POS
1430	Rhamnocitrin 3-glucoside	C22H22O11	M+FA-H	0.552144	507.11441	507.11469	19.581	NEG
1431	cucurbitoside e	C24H28O12	M-H	1.18308	507.1508	507.1514	18.723	NEG
1432	Gibberellin A95	C19H22O5	M+H	-0.332172	331.154	331.15389	20.624	POS
1433	(2R,5S)-5-methyl-2-[(E)-1-methyl-2-[(2R,3R,4S,5S,6R)-3,4,5-trihydroxy-6-methylol-tetrahydropyran-2-yl]oxy-vinyl]cyclohexan-1-one	C16H26O7	M+H	2.6874	331.17513	331.17602	12.784	POS
1434	Patrinoside	C21H34O11	M+FA-H	-0.197158	507.20831	507.20821	16.937	NEG
1435	(1r,4s,6s)-6-hydroxycamphorβ-d-apiofura-nosyl-(1→6)-β-d-	C21H34O11	M+FA-H	-0.906925	507.20831	507.20785	14.729	NEG

	glucopyranoside							
1436	norrufescine	C18H15NO4	M+Na	0.903371	332.08933	332.08963	23.586	POS
1437	6-dimethoxy-isoflavane	C23H28O10	M+FA-H	0.883797	509.16645	509.1669	19.939	NEG
1438	glucogallin	C13H16O10	M+H	-1.411066	333.08162	333.08115	7.646	POS
1439	6-Methylgingediol	C18H30O4	M+Na	-0.210082	333.20363	333.20356	27.905	POS
1440	erlangerin D	C25H24O9	M+FA-H	0.72105	513.14023	513.1406	20.923	NEG
1441	Psoralenol	C20H18O5	M+H	-0.884636	339.1227	339.1224	24.96	POS
1442	Cistanoside E	C21H32O12	M+FA-H	-0.460487	521.18758	521.18734	10.536	NEG
1443	(+) isolariciresinol 9-o-β-d-glucopyranoside	C26H34O11	M-H	1.59247	521.20284	521.20367	17.333	NEG
1444	Sulfoorientalol D	C15H26O5S	M+Na	-3.165862	341.13931	341.13823	19.141	POS
1445	piperkadsin C	C20H20O5	M+H	-0.469018	341.13835	341.13819	20.624	POS
1446	(1R,5R,6R,7R)-3-allyl-6-(1,3-benzodioxol-5-yl)-1-methoxy-7-methylbicyclo[3.2.1]oct-2-ene-4,8-dione	C20H20O5	M+H	1.934699	341.13835	341.13901	22.646	POS
1447	lycoposerramine n	C18H27NO4	M+Na	-4.53247	344.18323	344.18167	11.849	POS
1448	Penduletin	C18H16O7	M+H	1.622733	345.09688	345.09744	24.126	POS
1449	6'-O-malonylglycitin	C25H24O13	M-H	-2.654795	531.11441	531.113	20.46	NEG
1450	Gypsogenic acid	C30H46O5	M+FA-H	1.373904	531.33272	531.33345	31.903	NEG

1451	thymuside a	C16H26O7	M+NH4	-0.344628	348.20168	348.20156	16.963	POS
1452	Leonuridine	C15H24O9	M+H	0.171846	349.14931	349.14937	12.141	POS
1453	galloylhamame lose	C13H16O10	M+NH4	-2.456384	350.10817	350.10731	4.069	POS
1454	Silyamandin	C25H22O11	M+FA-H	-5.210688	543.11441	543.11158	20.481	NEG
1455	Gancaonin G	C21H20O5	M+H	-0.028318	353.13835	353.13834	31.838	POS
1456	5-methylcoumarin-4-gentiobioside	C22H28O13	M+FA-H	-4.787663	545.15119	545.14858	11.914	NEG
1457	poricoic acid E	C30H44O6	M+FA-H	1.430374	545.31199	545.31277	28.975	NEG
1458	ganolucidic acid D	C30H44O6	M+FA-H	0.770201	545.31199	545.31241	24.761	NEG
1459	6,7,10-Trihydroxy-8-octadecenoic acid	C18H34O5	M+Na	0.764375	353.22984	353.23011	24.502	POS
1460	Eriojaposide A	C24H38O11	M+FA-H	-0.182735	547.23961	547.23951	18.049	NEG
1461	Leonuriside A	C14H20O9	M+Na	3.210364	355.09995	355.10109	8.123	POS
1462	3'-Hydroxy-4'-methoxyglabridin	C21H22O5	M+H	0.394195	355.154	355.15414	28.519	POS
1463	yemuoside ym1	C25H30O11	M+FA-H	0.36286	551.17701	551.17721	14.236	NEG
1464	1-Methoxyphaseollidin	C21H22O5	M+H	0.563136	355.154	355.1542	29.171	POS
1465	(1r,2r)-p-menth-4(5)-ene-1,2-diol 1-o-β-d-glu-copyranoside	C16H28O7	M+Na	-0.957281	355.17272	355.17238	14.258	POS
1466	amomumoside	C16H28O7	M+Na	-0.90097	355.17272	355.1724	15.894	POS
1467	(1r,2r)-p-menth-3-ene-	C16H28O7	M+Na	-0.844659	355.17272	355.17242	15.297	POS

	1,2-diol 2-o-β-d-gluco-pyranoside							
1468	thalictircine	C20H21NO5	M+H	1.319671	356.14925	356.14972	20.981	POS
1469	2-(4-hydroxy-3-methoxyphenyl)-5-(3-hydroxypropyl)-7-methoxy-3-benzofurancarboxaldehyde	C20H20O6	M+H	0	357.13326	357.13326	23.039	POS
1470	Leachianone G	C20H20O6	M+H	0.308008	357.13326	357.13337	25.107	POS
1471	poricoic acid AM	C32H48O5	M+FA-H	0.538263	557.34837	557.34867	31.974	NEG
1472	dihydroisocucurbitacin-beta-25-acetate	C32H48O8	M-H	1.716346	559.32764	559.3286	30.656	NEG
1473	poricoic acid F	C31H46O6	M+FA-H	1.841497	559.32764	559.32867	29.251	NEG
1474	Quercetin 5,7,3',4'-tetramethyl ether	C19H18O7	M+H	-0.055693	359.11253	359.11251	28.75	POS
1475	Isochlorogenic acid b	C25H24O12	M+FA-H	0.908888	561.12498	561.12549	18.83	NEG
1476	2'-O-p-Coumaroylaloesin	C25H24O12	M+FA-H	1.158387	561.12498	561.12563	20.251	NEG
1477	3-o-angeloylhamaudol	C20H22O6	M+H	1.503555	359.14891	359.14945	28.962	POS
1478	MGMG 18:2	C27H48O9	M+FA-H	1.621155	561.32803	561.32894	31.567	NEG
1479	(+)-Thaliporphine	C20H23NO4	M+NH4	0.946557	359.19653	359.19687	20.583	POS
1480	Veranisatin A	C16H22O8	M+NH4	0.805186	360.16529	360.16558	15.046	POS
1481	Ganoderic acid C2	C30H46O7	M+FA-H	0.763328	563.32255	563.32298	23.528	NEG

1482	javanicolide c	C26H36O11	M+FA-H	3.373013	569.22396	569.22588	21.028	NEG
1483	Gibberellin A87	C19H22O7	M+H	0.771044	363.14383	363.14411	12.493	POS
1484	bidensyneoside c	C16H22O8	M+Na	-0.219106	365.12069	365.12061	9.233	POS
1485	3,5-di-O-caffeoylquinic acid methyl ester	C26H26O12	M+FA-H	-1.634383	575.14063	575.13969	18.723	NEG
1486	vitexin-2"-o-p-coumarate	C30H26O12	M-H	-3.396085	577.13515	577.13319	16.875	NEG
1487	(2S)-Naringenin 8-C-alpha-L-rhamnopyranosyl-(1->2)-beta-D-glucopyranoside	C27H32O14	M-H	0.120862	579.17193	579.172	13.039	NEG
1488	phellavin	C26H32O12	M+FA-H	-1.1184	581.18758	581.18693	20.128	NEG
1489	Citrusin A	C26H34O12	M+FA-H	0.703014	583.20323	583.20364	15.654	NEG
1490	Ugonin C	C21H20O6	M+H	-0.406357	369.13326	369.13311	29.659	POS
1491	Isoglycycoumarin	C21H20O6	M+H	-1.11071	369.13326	369.13285	30.847	POS
1492	Glyasperins M	C21H20O6	M+H	1.219072	369.13326	369.13371	26.864	POS
1493	Glycyrrhisoflavanone	C21H20O6	M+H	0.623081	369.13326	369.13349	26.461	POS
1494	Glyasperins K	C22H24O5	M+H	1.273127	369.16965	369.17012	31.644	POS
1495	6-c-arabinopyranosyl-8-c-glucopyranosyl-5,7-dihydroxyflavone	C26H28O13	M+FA-H	-2.731176	593.15119	593.14957	19.904	NEG
1496	Puerarin xyloside	C26H28O13	M+FA-H	-5.563506	593.15119	593.14789	14.088	NEG

1497	Schizonepetoside E	C16H28O8	M+Na	-0.107768	371.16764	371.1676	11.87	POS
1498	wallichinine	C22H26O5	M+H	1.912791	371.1853	371.18601	30.87	POS
1499	DIISOOCTYL ADIPATE	C22H42O4	M+H	1.211907	371.31559	371.31604	37.063	POS
1500	Xanthoxylol	C20H20O6	M+NH4	1.175968	374.15981	374.16025	22.21	POS
1501	Physcion-8-O-beta-D-gentiobioside	C28H32O15	M-H	2.799889	607.16684	607.16854	20.778	NEG
1502	(E)-3-[4-[(1R,2R)-2-hydroxy-2-(4-hydroxy-3-methoxy-phenyl)-1-methylol-ethoxy]-3-methoxy-phenyl]acrolein	C20H22O7	M+H	-0.186595	375.14383	375.14376	20.165	POS
1503	8-hydroxypinoresinal	C20H22O7	M+H	-0.213252	375.14383	375.14375	21.672	POS
1504	albibrissinoside b	C27H34O16	M-H	4.044506	613.17741	613.17989	15.923	NEG
1505	JusticidinosideA	C28H28O13	M+FA-H	-3.516156	617.15119	617.14902	20.315	NEG
1506	3-o-(cis-p-coumaroyl)-alphitolicacid	C39H54O6	M-H	-0.453526	617.38476	617.38448	34.723	NEG
1507	rubicoumaric acid	C39H54O6	M-H	2.996511	617.38476	617.38661	33.744	NEG
1508	3-({1-[(4-carbamimidamidobutyl)carbamoyl]-3-methylbutyl}carbamoyl)oxirane-2-carboxylic acid	C15H27N5O5	M+Na	3.366734	380.19044	380.19172	2.588	POS

1509	6,8-di-C-beta-D-glucopyranosyl-chrysoeriol	C28H32O16	M-H	3.594572	623.16176	623.164	12.018	NEG
1510	3,5-dimethoxy-4-[(2S,3R,4S,5S,6R)-3,4,5-trihydroxy-6-(hydroxymethyl)oxan-2-yl]oxybenzoic acid	C15H20O10	M+Na	0.234929	383.09486	383.09495	9.417	POS
1511	Prebetanin	C24H26N2O16S	M-H	2.734095	629.09303	629.09475	7.305	NEG
1512	Gancaonin D	C21H20O7	M+H	-2.129161	385.12818	385.12736	21.877	POS
1513	Villosolside	C16H26O9	M+Na	-0.363498	385.1469	385.14676	10.373	POS
1514	trans-hydroxyxanthohumol	C22H24O6	M+H	0.908703	385.16456	385.16491	32.221	POS
1515	murrayatin	C20H26O6	M+Na	7.607186	385.16216	385.16509	29.617	POS
1516	1,2,6-Trigalloyl-beta-D-glucopyranose	C27H24O18	M-H	-0.251933	635.08899	635.08883	14.078	NEG
1517	Multiflorin A	C29H32O16	M-H	-1.180802	635.16176	635.16101	20.798	NEG
1518	Porson	C22H26O6	M+H	1.058938	387.18021	387.18062	22.834	POS
1519	Viscumneoside I	C27H32O15	M+FA-H	-0.670647	641.17232	641.17189	14.869	NEG
1520	E-6-O-p-methoxycinnamoyl scandoside methyl ester_qt	C20H20O8	M+H	0.128494	389.12309	389.12314	20.333	POS
1521	xanthohumol g	C21H24O7	M+H	0.976463	389.15948	389.15986	21.253	POS

1522	(2R,6x)-7-Methyl-3-methylene-1,2,6,7-octanetetrol 2-glucoside	C16H30O9	M+Na	0.462513	389.1782	389.17838	9.541	POS
1523	(1r,2r)-p-menth-4(5)-ene-1,2-diol1-o-β-d-(2-o-acetyl)glucopyranoside	C18H30O8	M+NH4	1.32576	392.22789	392.22841	20.185	POS
1524	5,7-dihydroxy-2-methyl-8-[(2S,3R,4S,5S,6R)-3,4,5-trihydroxy-6-(hydroxymethyl)oxan-2-yl]oxychromen-4-one	C16H18O10	M+Na	-0.101761	393.07921	393.07917	10.788	POS
1525	wikstroemin	C28H32O15	M+FA-H	1.913737	653.17232	653.17357	16.256	NEG
1526	Zivulgarin	C28H32O15	M+FA-H	1.546299	653.17232	653.17333	17.082	NEG
1527	(6R,9R)-3-Oxo-alpha-ionol glucoside	C19H30O7	M+Na	0.508662	393.18837	393.18857	19.326	POS
1528	(1x,2x)-Guaiacylglycerol 2-glucoside	C16H24O10	M+NH4	0.076109	394.17077	394.1708	5.805	POS
1529	n-methylatalaphylline	C24H27NO4	M+H	7.7879	394.20128	394.20435	34.737	POS
1530	Linarin isovalerate	C33H40O14	M-H	1.562418	659.23453	659.23556	21.678	NEG
1531	Syringin;Syringoside	C17H24O9	M+Na	0.936398	395.13125	395.13162	16.358	POS
1532	25-o-acetylcimigenol-3-o-beta-d-	C37H58O10	M-H	1.3154	661.39572	661.39659	32.094	NEG

	xylopyranoside							
1533	astragalosideII_qt	C37H60O10	M-H	1.944495	663.41137	663.41266	29.021	NEG
1534	6-O-methylcatalpol	C16H24O10	M+Na	0.325712	399.12616	399.12629	4.151	POS
1535	(3E,4R)-4-(1,3-benzodioxol-5-ylmethyl)-3-[(3,4,5-trimethoxyphenyl)methylidene]oxolan-2-one	C22H22O7	M+H	0.851823	399.14383	399.14417	24.813	POS
1536	actein	C37H56O11	M-H	1.406626	675.37499	675.37594	31.032	NEG
1537	coniselin	C21H20O8	M+H	0.67311	401.12309	401.12336	23.64	POS
1538	ligusinenoside B	C32H44O16	M-H	-0.204901	683.25566	683.25552	15.799	NEG
1539	7-o-(4-β-d-glucopyranosyloxy-3-methoxy-benzoyl) secologanolic acid	C30H40O18	M-H	2.41555	687.21419	687.21585	17.881	NEG
1540	apocynoside ii	C19H30O9	M+H	0.744055	403.19626	403.19656	16.087	POS
1541	Licorice glycoside E	C35H35NO14	M-H	0.650103	692.19848	692.19893	21.7	NEG
1542	ketologanin	C17H24O10	M+NH4	0.861707	406.17077	406.17112	11.369	POS
1543	dehydrologanin	C17H24O10	M+NH4	-0.960187	406.17077	406.17038	10.435	POS
1544	fissistigmoside	C17H24O10	M+Na	0.437822	411.12616	411.12634	11.056	POS
1545	staphylionoside h	C19H32O8	M+Na	0.29183	411.19894	411.19906	13.908	POS
1546	Isoliquiritigenin 4-O-(5'''-O-feruloyl)-	C36H38O16	M-H	3.143923	725.20871	725.21099	20.736	NEG

	apiofuranosyl-(1'''->2'')-glucoside							
1547	harpagide acetate	C17H26O10	M+Na	-1.234443	413.14182	413.14131	11.369	POS
1548	Neoisostegane	C23H26O7	M+H	0.746673	415.17513	415.17544	31.277	POS
1549	Bayin	C21H20O9	M+H	-3.068676	417.11801	417.11673	14.115	POS
1550	eupalinilide f	C20H26O8	M+Na	5.273857	417.15199	417.15419	20.333	POS
1551	phyllaemblicin B	C33H44O19	M-H	3.33674	743.2404	743.24288	16.466	NEG
1552	(-)-olivil 4',4''-di-o-beta-d-glucopyranoside	C32H44O17	M+FA-H	0.576983	745.25605	745.25648	14.173	NEG
1553	1,3-dihydroxy-6,7-dimethylxanthone-1-o-beta-d-glucoside	C21H22O9	M+H	-1.574677	419.13366	419.133	18.678	POS
1554	Aloin A	C21H22O9	M+H	-2.027993	419.13366	419.13281	13.032	POS
1555	glomeratose c	C33H40O17	M+FA-H	0.796575	753.22475	753.22535	20.651	NEG
1556	spionoside b	C19H30O9	M+NH4	-0.571126	420.22281	420.22257	16.941	POS
1557	yadanzioside G	C36H48O18	M-H	4.874382	767.27679	767.28053	19.308	NEG
1558	Glyasperin A	C25H26O6	M+H	1.157899	423.18021	423.1807	32.285	POS
1559	Yinyanghuo B	C25H26O6	M+H	0.567134	423.18021	423.18045	28.265	POS
1560	Acetylcatalpol	C18H26O10	M+Na	0.493953	425.14182	425.14203	12.205	POS
1561	insularoside-3'-o-beta-glucoside	C38H46O18	M-H	-1.165647	789.26114	789.26022	17.125	NEG
1562	licoricesaponine c2	C42H62O15	M-H	1.576853	805.4016	805.40287	29.479	NEG

1563	3-O-beta-D-Glucuronopyranosyl gypsogenin	C42H64O15	M-H	2.018783	807.41725	807.41888	24.953	NEG
1564	Abrusoside C	C42H64O15	M-H	1.956857	807.41725	807.41883	23.487	NEG
1565	Azukisaponin III	C42H66O15	M-H	3.273897	809.4329	809.43555	26.899	NEG
1566	caesaldekarine	C24H30O6	M+NH4	0.832874	432.23806	432.23842	29.321	POS
1567	2-o-caffeoyl arbutin	C21H22O10	M+H	0.13789	435.12857	435.12863	18.678	POS
1568	Kaempferol 7-arabinoside	C20H18O10	M+NH4	1.192322	436.12382	436.12434	20.77	POS
1569	Yiamoloside B	C43H68O15	M-H	2.319513	823.44855	823.45046	27.146	NEG
1570	Kaempferol-3-O-arabinoside	C20H18O10	M+NH4	1.26111	436.12382	436.12437	18.111	POS
1571	7-o-acetylloganic acid	C18H26O11	M+NH4	-1.10046	436.18133	436.18085	10.413	POS
1572	(+)-Syringaresinol-di-O-β-D-glucosid _qt	C22H26O8	M+NH4	1.215048	436.19659	436.19712	19.285	POS
1573	2,6,2',6'-tetramethoxy-4,4'-bis(2,3-epoxy-1-hydroxypropyl)biphenyl	C22H26O8	M+NH4	2.246693	436.19659	436.19757	16.525	POS
1574	Gitoxin	C41H64O14	M+FA-H	5.621327	825.42781	825.43245	20.651	NEG
1575	Saikosaponin A 柴胡皂苷	C42H68O13	M+FA-H	1.732359	825.46419	825.46562	23.167	NEG
1576	Verbascose	C30H52O26	M-H	2.52639	827.26741	827.2695	1.465	NEG
1577	Vinaginsenoside R2	C43H72O15	M-H	1.474356	827.47985	827.48107	23.57	NEG
1578	caesalmin a	C22H28O8	M+NH4	1.20946	438.21224	438.21277	17.339	POS
1579	astragalosideI	C43H68O16	M-H	3.716748	839.44346	839.44658	26.749	NEG

1580	icariside c3	C21H38O8	M+Na	3.263486	441.24589	441.24733	20.417	POS
1581	siraitic acid C	C28H40O4	M+H	0.113302	441.29994	441.29999	35.632	POS
1582	6-Methoxyl-2-acetyl-3-methyl-1,4-naphthoquinone-8-O-beta-D-glucopyranoside	C20H22O10	M+Na	4.201204	445.11051	445.11238	20.292	POS
1583	p-β-rutinosyloxystyrene	C20H28O10	M+NH4	1.053334	446.20207	446.20254	18.805	POS
1584	[2-[(2S,3R,4S,5S,6R)-3,4,5-trihydroxy-6-(hydroxymethyl)oxan-2-yl]oxyphenyl]methyl (E)-3-(3,4-dihydroxyphenyl)prop-2-enoate	C22H24O10	M+H	-1.224551	449.14422	449.14367	20.144	POS
1585	2,3,5,4'-tetrahydroxystilbene-2-o-(6''-o-acetyl)-beta-d-glucopyranoside	C22H24O10	M+H	-1.580784	449.14422	449.14351	19.863	POS
1586	dichotomoside,c_qt	C23H28O9	M+H	1.424817	449.18061	449.18125	20.96	POS
1587	8-o-acetylshanzhiside	C18H26O12	M+NH4	0.42019	452.17625	452.17644	8.324	POS
1588	secologanin dimethyl acetal	C19H30O11	M+NH4	0.50861	452.21263	452.21286	15.546	POS
1589	JHDRQQVCLPTAQB-	C42H67O17S-	M-	-4.112357	875.41045	875.40685	24.953	NEG

	UHFFFAOYSA-M							
1590	aloe-emodin dianthrone diglucoside	C42H42O18	M+FA-H	2.26333	879.23531	879.2373	19.351	NEG
1591	Osladin	C45H74O17	M-H	5.014199	885.48532	885.48976	22.416	NEG
1592	Embigenin	C23H24O10	M+H	4.749057	461.14422	461.14641	20.833	POS
1593	Mudanoside B	C18H24O14	M+H	-1.676973	465.12388	465.1231	6.723	POS
1594	hydrocotyloside i	C47H74O19	M-H	2.676651	941.47515	941.47767	22.563	NEG
1595	(1's)-1'-(4-hydroxyphenyl)ethane-1',2'-diol2'-o-β-d-apiofuranosyl-(1→6)-β-d-glucopyranoside	C19H28O12	M+NH4	-0.171603	466.1919	466.19182	9.279	POS
1596	Licoricesaponin F3	C48H72O19	M-H	0.073571	951.4595	951.45957	27.696	NEG
1597	borneol-2-o-α-l-arabinofuranosyl(1→6)-β-d-glucopyranoside	C21H36O10	M+NH4	-1.072352	466.26467	466.26417	21.317	POS
1598	Abrisaponin 1	C48H74O20	M-H	-0.010315	969.47007	969.47006	22.059	NEG
1599	Catechin 7-glucoside	C21H24O11	M+NH4	-3.700823	470.16568	470.16394	10.165	POS
1600	10,12-dihy-droxypicrotoxane	C20H23N7O7	M+H	-0.611591	474.17317	474.17288	9.808	POS
1601	Licoricesaponin D3	C50H76O21	M-H	-0.652509	1011.48063	1011.47997	23.634	NEG
1602	stachysterone D	C27H42O6	M+NH4	1.394869	480.33196	480.33263	25.699	POS
1603	3,4-dihydroxyally	C21H30O11	M+Na	5.341169	481.16803	481.1706	14.093	POS

	lbenzene 4-o-[alpha-l-rhamnopyranosyl-(1-6)]-beta-d-glucopyranoside							
1604	(2 e)-2-decene-4,6-diyne-1,8-diol 8-o-β-d-apio-furanosyl-(1→6)-β-d-glucopyranoside	C21H30O11	M+Na	5.278821	481.16803	481.17057	16.859	POS
1605	pennogenin rhamnosyl chacotrioside	C51H82O21	M-H	2.641988	1029.52758	1029.5303	23.908	NEG
1606	3β,5α,9α,14β-tetrahydroxy-(22e)-ergosta-7,22-dien-6-one	C28H44O5	M+Na	7.117613	483.30809	483.31153	29.767	POS
1607	Marchantin G 1	C28H22O6	M+K	5.678306	493.1048	493.1076	26.65	POS
1608	Saikosaponin V	C53H86O24	M+FA-H	-0.191047	1151.5491	1151.54888	19.832	NEG
1609	2-(3-Hydroxy-4-methoxyphenyl)ethyl 3-O-(6-deoxyhexopyranosyl)hexopyranoside	C21H32O12	M+NH4	1.355663	494.2232	494.22387	11.87	POS
1610	2α,3α,19α-trihydroxy-24-norurs-4(23),12-dien-28-oicacid	C29H44O5	M+Na	0.787389	495.30809	495.30848	28.581	POS
1611	Kelampayoside A	C20H30O13	M+Na	2.174963	501.15786	501.15895	12.887	POS

1612	paniculatonoid a	C54H66O31	M+FA-H	-7.997725	1255.357	1255.34696	20.128	NEG
1613	Ganolucidic acid A	C30H44O6	M+H	2.134361	501.32107	501.32214	28.391	POS
1614	tenuifoliose k	C57H70O32	M-H	-2.765972	1265.37774	1265.37424	20.128	NEG
1615	sophoraflavoside	C59H96O27	M+FA-H	-0.444752	1281.6121	1281.61153	22.836	NEG
1616	shionoside C	C24H40O10	M+NH4	-0.967813	506.29597	506.29548	24.665	POS
1617	Theasapogenol A	C30H50O6	M+H	-0.216805	507.36802	507.36791	22.977	POS
1618	4-O-methylpaeoniflorin	C24H30O11	M+NH4	0.722356	512.21263	512.213	17.654	POS
1619	(6s,9r)-vomifoliol-9-o-β-apiofuranosyl-(1"→6')-o-β-glucopyranoside	C24H38O12	M+H	0.269623	519.2436	519.24374	13.846	POS
1620	gibberellin glucoside III	C25H32O11	M+NH4	0.380063	526.22828	526.22848	15.129	POS
1621	belamcandal	C32H48O6	M+H	1.851319	529.35237	529.35335	30.675	POS
1622	tuberculatin	C26H24O11	M+NH4	0.150896	530.16568	530.16576	21.212	POS
1623	divostroside	C30H46O8	M+H	3.100911	535.32654	535.3282	21.36	POS
1624	dihydrodehydrodiconiferyl alcohol 4'-o-β-d-glucoside	C26H34O11	M+NH4	-0.629345	540.24393	540.24359	21.085	POS
1625	p-methoxycinnamoylcatalpol	C25H30O12	M+Na	2.036089	545.16294	545.16405	18.87	POS
1626	lactucain b	C30H32O9	M+NH4	1.876449	554.23846	554.2395	23.162	POS
1627	(?)-epicatechin-5-o-β-d-glucopyranosyl-3-benzoate	C28H28O12	M+H	1.07688	557.16535	557.16595	21.692	POS

1628	sesquipinsapol B	C30H36O9	M+NH4	-0.609025	558.26976	558.26942	21.233	POS
1629	picrasidine u	C30H24N4O5	M+K	0	559.13783	559.13783	23.586	POS
1630	cucurbitoside a	C26H32O12	M+Na	-1.162419	559.17859	559.17794	20.144	POS
1631	(2S,3R,4S,5S,6R)-2-(4-((2S,3R,4S)-4-Hydroxy-4-(4-hydroxy-3-methoxybenzyl)-3-(hydroxymethyl)tetrahydrofuran-2-yl)-2-methoxyphenoxy)-6-(hydroxymethyl)tetrahydro-2H-pyran-3,4,5-triol	C26H34O12	M+Na	1.051329	561.19424	561.19483	15.254	POS
1632	10-o-e-feruloylmonotropein	C26H30O14	M+H	0.12342	567.17083	567.1709	19.223	POS
1633	lactucaside	C26H32O13	M+NH4	0.508577	570.21811	570.2184	14.238	POS
1634	Lappaol E	C30H34O10	M+NH4	0.104849	572.24902	572.24908	21.712	POS
1635	isolappaol C	C30H34O10	M+Na	2.252235	577.20442	577.20572	21.712	POS
1636	Caesappanin	C32H32O10	M+H	-7.709542	577.20682	577.20237	12.969	POS
1637	Procyanidin B-23,3'-di-O-gallate_qt	C30H26O12	M+H	0.777001	579.1497	579.15015	10.58	POS
1638	Daidzein 4',7-diglucoside	C27H30O14	M+H	3.107891	579.17083	579.17263	12.659	POS
1639	macrophyllosìde d	C25H34O14	M+Na	5.953363	581.18407	581.18753	21.692	POS
1640	5-o-p-methoxy cinnamoyl	C27H32O13	M+NH4	1.958029	582.21811	582.21925	20.917	POS

	scandoside methylester							
1641	(2R)-8-[(2S,3R,4S,5S,6R)-4,5-dihydroxy-6-methylol-3-[(2S,3R,4R,5R,6S)-3,4,5-trihydroxy-6-methyl-tetrahydropyran-2-yl]oxy-tetrahydropyran-2-yl]-5,7-dihydroxy-2-(4-hydroxyphenyl)chroman-4-one	C27H32O14	M+Na	0.480794	603.16842	603.16871	16.589	POS
1642	abrusin-2''-o-apioside	C28H34O15	M+H	1.325268	611.19705	611.19786	17.945	POS
1643	Butrin	C27H32O15	M+NH4	-0.700089	614.20794	614.20751	14.874	POS
1644	3,8-Di-C-glucopyranosyldiosmetin	C28H32O16	M+H	-1.98344	625.17631	625.17507	15.337	POS
1645	Isorhamnetin-3-O-neohesperidoside	C28H32O16	M+H	1.519571	625.17631	625.17726	19.841	POS
1646	Leiocarposide	C27H34O16	M+NH4	0.980674	632.21851	632.21913	15.941	POS
1647	amphibine D	C36H49N5O5	M+H	1.280874	632.38065	632.38146	21.401	POS
1648	24-hydroxy-12beta-acetoxy-25,26,27-trinorcycloartan-16,23-dione,3beta-o-alpha-l-	C34H52O10	M+NH4	1.832736	638.38987	638.39104	23.599	POS

	arabinopyranoside							
1649	5'-hydroxyiso-muronulatol-2',5'-di-O-glucoside	C29H38O16	M+NH4	1.272246	660.24981	660.25065	14.053	POS
1650	Cristatain	C36H54O11	M+H	0.618053	663.37389	663.3743	23.306	POS
1651	yadanzioside I	C29H38O16	M+Na	3.202019	665.2052	665.20733	19.49	POS
1652	oleracein c	C30H35NO16	M+H	1.185825	666.20286	666.20365	14.942	POS
1653	cistanoside D	C31H40O15	M+NH4	1.566532	670.27054	670.27159	18.536	POS
1654	Fetidine	C40H46N2O8	M+H	-1.360977	683.33269	683.33176	13.802	POS
1655	Isonuezhenide	C31H42O17	M+Na	5.555309	709.23142	709.23536	19.346	POS
1656	6'-p-hydroxybenzoylspinosin	C35H36O17	M+H	2.002187	729.20253	729.20399	19.162	POS
1657	(+)-Medioresinol di-O-beta-D-glucopyranoside	C33H44O17	M+NH4	0.082159	730.29167	730.29173	16.484	POS
1658	Narirutin 4'-glucoside	C33H42O19	M+H	2.260376	743.23931	743.24099	16.755	POS
1659	Naringin 4'-glucoside	C33H42O19	M+NH4	0.07892	760.26585	760.26591	14.982	POS
1660	(3R,8S,9R,10R,13R,14S,17R)-3-hydroxy-4,4,9,13,14-pentamethyl-17-[(E,2R)-6-methyl-7-[(2R,3R,4S,5S,6R)-3,4,5-trihydroxy-6-[[(2R,3R,4S,5S,6R)-3,4,5-	C42H68O13	M+H	-1.074893	781.47327	781.47243	22.457	POS

	trihydroxy-6-(hydroxymethyl)oxan-2-yl]oxymethyl]oxan-2-yl]oxyhept-5-en-2-yl]-1,2,3,7,8,10,12,15,16,17-decahydr							
1661	(4aS,6aR,6aS,6bR,8aR,9R,10S,12aR,14bS)-10-[[(2R,3R,4S,5S,6S)-4,5-dihydroxy-6-methyl-3-[[(2S,3R,4R,5R,6S)-3,4,5-trihydroxy-6-methyl-2-tetrahydropyranyl]oxy]-2-tetrahydropyranyl]oxy]-9-(hydroxymethyl)-2,2,6a,6b,9,12a-hexamethyl-1,3,4,5,6,6a,7,8,8a,10,11,12,	C42H68O12	M+Na	5.016126	787.4603	787.46425	27.738	POS
1662	taxezopidine l	C39H46O15	M+K	-0.088245	793.24683	793.24676	29.234	POS
1663	Spinosin 6'''-(E)-sinapoyl ester	C39H42O19	M+H	1.140769	815.23931	815.24024	19.943	POS
1664	licoricesaponin e2	C42H60O16	M+H	2.300962	821.39541	821.3973	25.489	POS
1665	Licoricesaponin K2	C42H62O16	M+H	3.145452	823.41106	823.41365	26.95	POS

1666	Diammonium glycyrrhizinate	C42H62O16	M+H	3.242609	823.41106	823.41373	21.421	POS
1667	Licoricesaponin J2	C42H64O16	M+H	1.235724	825.42671	825.42773	28.897	POS
1668	(2S,3S,4S,5R,6R)-6-[[(3S,4aR,6aR,6bS,8aS,11R,12R,12aS,14aR,14bR)-12-hydroxy-4,4,6a,6b,11,12,14b-heptamethyl-8a-[oxo-[[(2S,3R,4S,5S,6R)-3,4,5-trihydroxy-6-(hydroxymethyl)-2-tetrahydropyranyl]oxy]methyl]-1,2,3,4a,5,6,7,8,9,10,11,12a,14,14a-tetradecahydropic	C42H66O15	M+Na	4.031535	833.42939	833.43275	27.124	POS
1669	thevetin B	C42H66O18	M+Na	2.0989	881.41413	881.41598	21.979	POS
1670	syringylglycerol-β-syringaresinol ether-4",4'"-di-o-β-d-glucopyranoside	C45H60O23	M+NH4	-1.257114	986.38636	986.38512	19.387	POS
1671	glycoside h1(periplocae)	C56H92O24	M+Na	-1.877795	1171.58707	1171.58487	27.675	POS
